# Spatial analyses implicate high stromal tumour-infiltrating CD8^+^ lymphocytes as a negative predictive marker for chemotherapy in estrogen receptor-positive breast cancer

**DOI:** 10.1038/s41467-026-73432-2

**Published:** 2026-06-23

**Authors:** Zak Kinsella, Chowdhury Arif Jahangir, Hannah Nyarkoah Nyarko, Daria Kalinska-Lysiak, Claudia Aura Gonzalez, Verena Murphy, Tony O’Grady, Joanna Fay, Katherine Sheehan, Arman Rahman, John P. Crown, Catherine M. Kelly, Simon S. McDade, Ina Woods, Niamh M. C. Connolly, Jochen H. M. Prehn, William M. Gallagher, Darran P. O’Connor

**Affiliations:** 1 School of Pharmacy & Biomolecular Sciences, Royal College of Surgeons in Ireland, Dublin, Ireland; 2 UCD Conway Institute, University College Dublin, Dublin, Ireland; 3 Cancer Trials Ireland, Dublin, Ireland; 4 Department of Pathology, Royal College of Surgeons in Ireland, Dublin, Ireland; 5 St. Vincent’s University Hospital, Dublin, Ireland; 6 Mater Hospital, Dublin, Ireland; 7 Johnston Cancer Research Centre (JCRC), Queens University Belfast, Belfast, UK; 8 Department of Physiology & Medical Physics, Centre for Systems Medicine, Royal College of Surgeons in Ireland, Dublin, Ireland

**Keywords:** Breast cancer, Cancer microenvironment, Systems analysis

## Abstract

Female patients with ER^+^HER2^-^ breast cancer have a favourable prognosis for 5-10 years. Later relapses are, however, common, yet predictions of late recurrence risk are suboptimal, particularly for patients with intermediate risk determined by the Oncotype Dx Recurrence Score (RS, 16-25). Here, we analyse tissue samples from patients with ER^+^HER2^-^ breast cancer using spatial proteomics (multiplex immunofluorescence with 5 markers, *n* = 440) and spatial transcriptomics (*n* = 359), and find decoupled immune states between stroma and epithelia. Moreover, inflamed stroma express genes linked to tissue remodelling, immune exhaustion, and inhibitory/checkpoint receptors (*CTLA4, TIGIT, CD96*); inflamed epithelia similarly express genes associated with checkpoints (*CTLA4*) and exhaustion (*CXCL13*), but also genes attributed to antigen presentation. In our randomised, Intermediate RS cohort treated with chemotherapy we observe an association between higher stromal tumour-infiltrating CD8^+^ lymphocyte (sTIL CD8^+^) density and poor outcome (ΔLR-χ^2^: 6.79, *p* = 0.009), which we validate using data from whole-resection specimens (ΔLR-χ^2^: 8.90, *p* = 0.003). Our data thus provide insights into the immune states in ER^+^HER2^-^ breast cancer, and propose sTIL CD8^+^ density as candidate biomarker for treatment decisions.

## Abstract (web summary)

Predicting the relapse of ER^+^HER2^-^ breast cancer remains challenging. Here, the authors use spatial transcriptomics and multiplex immunofluorescence to analyze ER^+^ breast cancers samples to find distinct stromal and epithelial immune states, and to propose high stromal CD8^+^ tumor-infiltrating lymphocyte density as a negative predictive biomarker for chemotherapy.

## Introduction

Estrogen receptor-positive (ER^+^) disease is conventionally viewed as an ‘immunologically cold’ subtype of breast cancer, with lower infiltration of tumour-infiltrating lymphocytes (TILs) in comparison to more clinically aggressive subtypes such as human epidermal growth factor receptor 2-positive (HER2+) and triple-negative breast cancer (TNBC)^1,2^, as defined by an average infiltration ≤10% TILs^3^. Despite this being an accepted threshold for the categorical definition of immune infiltration, i.e. hot or not, there is a mounting body of evidence to suggest that the immune microenvironments of early-stage disease, including the ER^+^HER2^−^ subtype, is non-homogenous^4^. This is largely due to underlying genomic instabilities^5^, their phenotypes^6^, and resulting spatial structures^7^. Consequently, these data may be useful prognostic indicators or predictive measures of treatment benefit, such as immunotherapies^8–10^ or chemotherapy.

The immune microenvironment in ER^+^ disease is infiltrated by cells of both myeloid and lymphoid lineages, though the prognostic efficacy of their scoring in large pilot studies has been disappointing. Major effectors of the adaptive immune response (CD8^+^ T-cells) are not considered to stratify for improved outcome^11^. Helper phenotypes such as CD4^+^ T-helper and regulatory CD4^+^FOXP3^+^ T-reg cells confer marginal prognostic information^12–14^ or none at all, though CD20^+^ B-cells, particularly those enriched in tertiary lymphoid structures, show an association with Immune checkpoint inhibitor (ICI) response^7^. Despite lymphoid lineages commanding adaptive immune responses, the tumour microenvironment of ER^+^ disease is dominated by myeloid lineages, mostly macrophage^15,16^, but also containing large frequencies of stromal fibroblast subpopulations^17^ that appear to condition the acquiescence of immune responses to genomic events, or chemokine gradients, that would otherwise favour tumour killing.

Management of early-stage ER^+^HER2^−^ breast cancer also remains the subject of much debate, in which 3 ongoing prospective clinical trials (MINDACT, TAILORx and OPTIMA)^18–20^ aim to accurately determine the requirement of chemotherapy. While current chemotherapeutic regimens offer benefit to some, others who have an inherently reduced risk of recurrence still receive chemotherapy, where the majority would remain cancer-free without it^21,22^. The most widely used test to decide whether chemotherapy is required for ER^+^HER2^−^ disease is Oncotype Dx. However, it has several shortcomings with room for improvement, such as the uncertainty around the necessity of chemotherapy for managing pre-menopausal patients^23,24^.

**Fig. 1:**
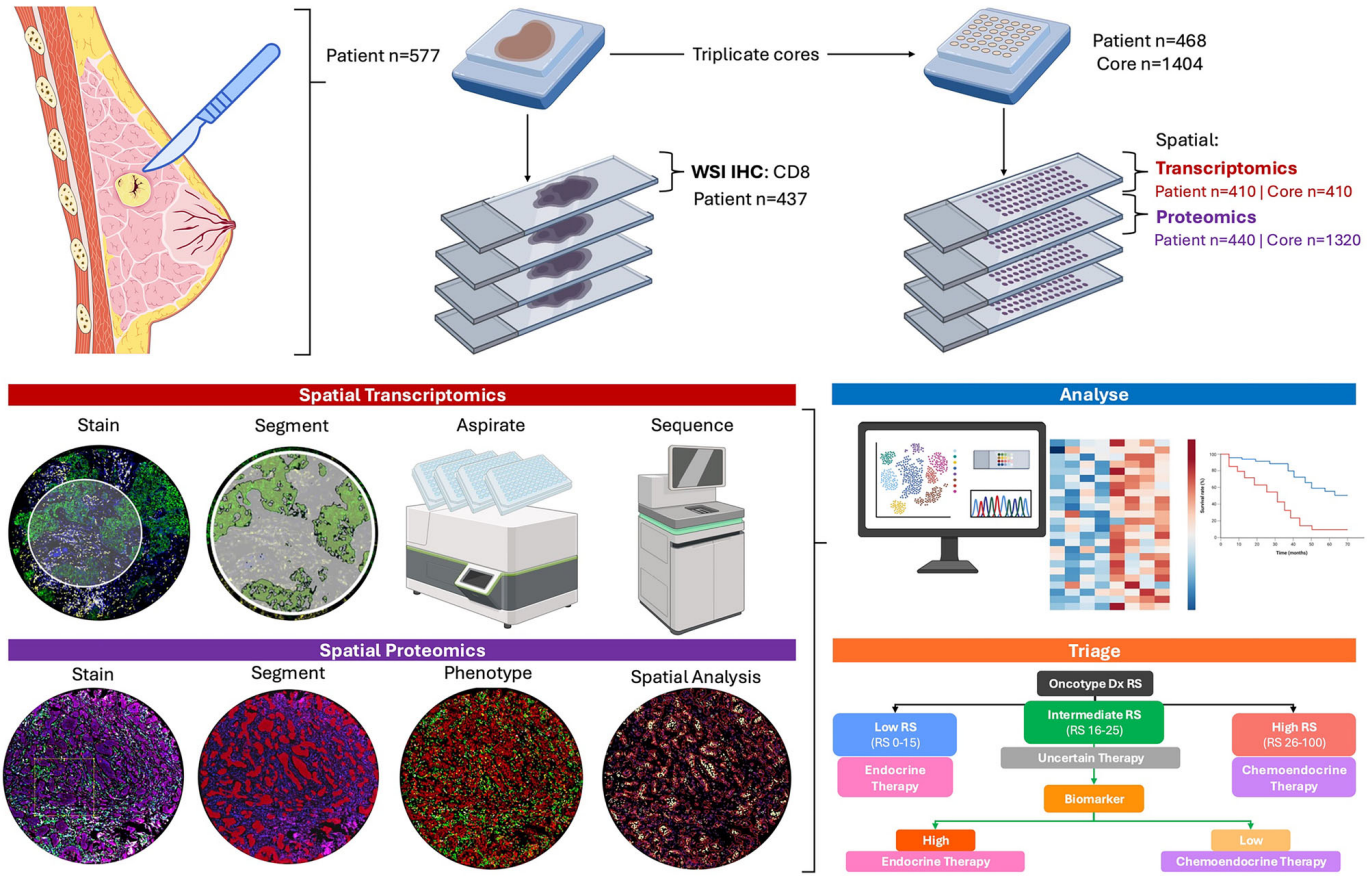
An overview of the workflow using whole resection specimen FFPE (formalin-fixed paraffin-embedded tissue) blocks for orthogonal validation, and TMA (tissue microarray) blocks for spatial-omics samples. Further information on patient sample size after dropout can be seen in CONSORT diagrams, Supplementary Fig. 1. Created in BioRender. Kinsella, Z. (2026) https://BioRender.com/330lqob.

In this study, we seek to provide more granular information on recurrence risk across Oncotype Dx RS categories using region of interest-based spatial transcriptomics and 5-marker multiplex immunofluorescence-based spatial proteomics (Fig. 1), in a cohort of Irish patients previously enrolled on the TAILORx clinical trial (clinical characteristics in Table 1. CONSORT diagram can be found in Supplementary Fig. 1). We discover a spectrum of immune subtypes across the epithelia and tumour microenvironment that are not significantly coupled nor captured by Oncotype Dx RS-derived genomic risk. Using spatial transcriptomic data, we find that inflamed immune subtypes express extracellular matrix-remodelling, exhaustion-related, and checkpoint transcripts, and determine stromal cytotoxic T-cell density as a predictive biomarker for chemotherapy in patients with an Intermediate RS (RS 16–25). This discovery requires validation in the larger TAILORx trial before clinical adoption. Thus, our study shows that immune profiling can improve the Oncotype Dx RS by further refining the selection of patients requiring de-escalation from, or escalation to, adjuvant chemotherapy.

**Table 1:** Patient cohort characteristics of Irish patients previously enrolled in TAILORx (CTRIAL-IE 12-30, NCT02050750)

*N* = 577 Characteristic	Oncotype Dx RS		
	Low (0–15) 224 (38.8%)	Intermediate (16–25) 240 (41.6%)	High (26–100) 113 (19.6%)
Median age			
(Range)	53 (25–79)	54 (34–75)	55 (28–74)
Menopausal status			
Pre/perimenopausal	104 (46.4%)	95 (39.6%)	45 (39.8%)
Postmenopausal	120 (53.6%)	145 (60.4%)	70 (60.2%)
Tumour size			
T1	146 (65.2%)	159 (66.3%)	64 (56.6%)
T2	78 (34.8%)	79 (32.9%)	49 (43.4%)
Unknown	–	2 (0.8%)	–
Histological grade			
Grade I (low)	56 (25.0%)	31 (12.9%)	3 (2.6%)
Grade II (intermediate)	141 (62.9%)	166 (69.2%)	49 (43.4%)
Grade III (high)	22 (9.8%)	43 (17.9%)	61 (54.0%)
Unknown	5 (2.2%)	–	–
Histological subtype			
Invasive ductal carcinoma (IDC)	173 (77.2%)	189 (78.8%)	103 (91.2%)
Invasive lobular carcinoma (ILC)	31 (13.8%)	40 (16.7%)	6 (5.3%)
Other	20 (9.0%)	6 (2.5%)	4 (3.5%)
Adjuvant treatment			
Hormone therapy alone	161 (71.9%)	123 (51.2%)	1 (0.9%)
Hormone therapy + chemotherapy	63 (28.1%)	117 (48.8%)	112 (99.1%)
Luminal subtype			
Luminal A (Ki67 < 14%)	143 (64%)	115 (48%)	24 (21%)
Luminal B (Ki67 ≥ 14%)	16 (7%)	58 (24%)	59 (52%)
No Ki67 data	65 (29%)	68 (28%)	30 (27%)

‘Other’ histology includes: adenoid cystic, collision tumour, *IDC-ILC, DCIS,* invasive cribriform carcinoma, invasive mucinous carcinoma, invasive tubular carcinoma.

## Results

### Outlining the tumour-immune microenvironments of the Oncotype Dx RS

**Fig. 2:**
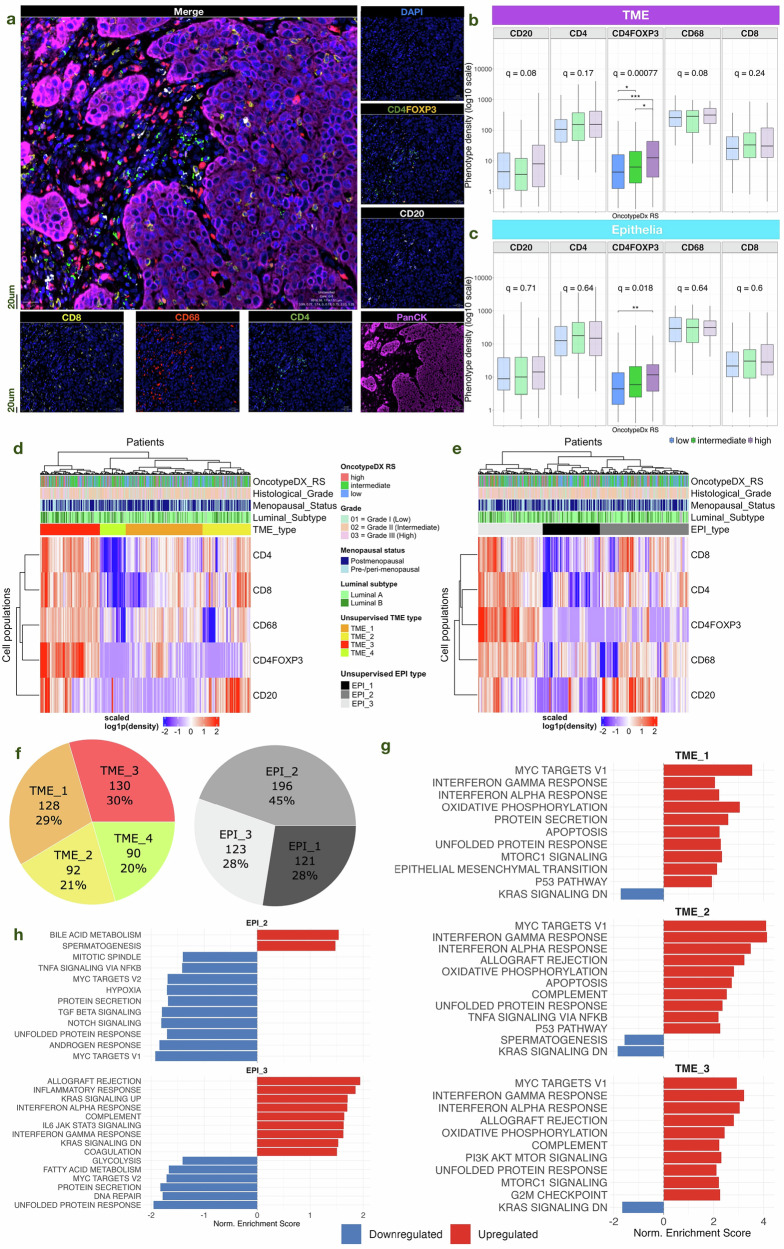
Outlining the immune milieu. **a** Representative ROIs (regions of interest) showing proteomic 7plex data across the tumour microenvironment (TME) and epithelia (EPI). Boxplots of log-transformed immune phenotype density across **b**. The TME (Low RS [recurrence score] *n* = 167, Int RS *n* = 183, High RS *n* = 90), **c***.* Epithelia (Low RS *n* = 167, Int RS *n* = 183, High RS *n* = 90). Box plots show the median (centre line), with the box spanning the interquartile range (25th to 75th percentiles). Whiskers extend to the most extreme values within 1.5 × the interquartile range from the box. Outliers beyond the whiskers were not displayed. Opacity encodes significance on the Kruskal–Wallis test, and *q* shows the Benjamini–Hochberg-adjusted *p*-value from the Kruskal–Wallis test. Asterisks denote significant pairwise comparisons of two-sided Dunn’s post hoc test, adjusted for multiple comparisons, with values as follows: *p* < 0.05 (*), 0.01 ≥ *p* > 0.001 (**), *p* ≤ 0.001 (***). Exact adjusted *p*-values: **b** CD4^+^FOXP3^+^ Low RS vs. High RS *p* = 0.0000873; Low RS vs. Int RS *p* = 0.0424; Int RS vs. High RS *p* = 0.0155. **c** CD4^+^FOXP3^+^ Low RS vs. High RS *p* = 0.00253. **d**–**f** Heatmaps of unsupervised clustering on immune phenotypes from spatial proteomic data, within the **d** TME, and **e** epithelia. **f** Pie charts of the frequency of patients with each TME and EPI state. Bar charts of gene set enrichment analysis (GSEA) for each unsupervised cluster versus the baseline, immune-cold state, for **g** TME States versus TME-4, **h** EPI states versus EPI-1.

Considering the consensus view of estrogen receptor-positive, human epidermal growth factor receptor 2-negative (ER^+^HER2^−^) breast cancer being an immune-cold subtype, we firstly investigated the immune composition of the tumour microenvironment (TME) and epithelia across the Oncotype Dx recurrence score (RS) (Fig. 2a). Using 5-marker multiplex immunofluorescence-based spatial proteomic data (*n* = 440), we discovered that within TME/stroma (i.e. stromal tumour-infiltrating lymphocytes: sTIL) no sTIL phenotype density was significantly different across the RS except for sTIL T-regulatory cells, that significantly increased with risk (Fig. 2b. Kruskal–Wallis *p* < 0.001. Dunn’s test High RS vs. Low RS *p* < 0.0001, High RS vs. Int RS *p* = 0.016, Int RS vs. Low RS *p* = 0.042. FDR < 0.05). Within epithelia (i.e. intraepithelial tumour-infiltrating lymphocytes: iTIL) (Fig. 2c), likewise only iTIL T-regulatory cells significantly increased (Kruskal–Wallis *q* = 0.018), with High RS disease contributing the majority of this significant difference (Dunn’s test High RS vs. Low RS *p* < 0.001, FDR < 0.05; High RS vs. Int RS *p* = 0.049, FDR > 0.05). We next explored with hierarchical clustering whether any immune pattern would emerge in both the stroma (TME, Fig. 2d) and epithelia (EPI, Fig. 2e). We found four TME and three EPI states. One state each was truly immune cold (TME-4: *n* = 90, 20%. EPI-1: *n* = 121, 28%. Fig. 2f) and one pan-immune hot (TME-3: *n* = 130, 30%. EPI-3: *n* = 123, 28%. Fig. 2f). Two TME and one EPI state were variably immune-infiltrated, with one more enriched in T-helper/cytotoxic T-cells (TME-2: *n* = 92, 21%. Fig. 2f) and the other more T-helper scarce (TME-1: *n* = 128, 29%, Fig. 2f), whereas EPI-2 appeared as an intermediary between hot and cold states (*n* = 196, 45%. Fig. 2f).

We next performed differential gene expression for each cluster relative to the immune-cold state (TME-4 or EPI-1), and gene-set enrichment analysis (GSEA) was subsequently performed to identify functionally enriched terms and pathways (Fig. 2g, h). We performed this in the matched spatial proteomic-transcriptomic dataset (*n* = 359). All TME states were dominated by immune activation and cytokine response programmes (e.g. enriched interferon-α and interferon-γ hallmarks), though differing in the type and intensity of inflammation. TME-1 (T-cell low) was enriched in epithelial-to-mesenchymal transition and P53 pathway, TGF-β signalling, apoptosis and oxidative phosphorylation. TME-2 (T-cell infiltrating) was enriched for allograft rejection, TNF signalling via NFκB, P53 pathway and complement hallmarks, whereas TME-3 (immune-hot) was uniquely enriched in PI3K-AKT-mTOR, mTORC1 signalling, and DNA damage checkpoint G2M, among enriched inflammatory pathways. Altogether, compared with immune cold TME-4, GSEA suggests TME-1 is a more activated/stressed stromal state with interferon-driven immune signalling; TME-2 more classically inflammatory; TME-3 is immune-hot but exhibits strong growth, metabolic and DNA damage responses indicative of a milieu with marked cell-cycle pressure and survival pathways (Fig. 2g). Comparatively, EPI-2 (immune-intermediary) was also independently associated with downregulation of TNF signalling, hypoxia, TGF-β signalling, Notch signalling and androgen response hallmarks, indicative of a less inflamed, less proliferative, and transcriptionally quiet epithelium. On the contrary, EPI-3 (immune-hot) was associated with downregulation of glycolysis, fatty acid metabolism, MYC targets and DNA repair, with enrichment of interferon-α/ interferon-γ hallmarks, inflammatory response pathways, IL6-JAK-STAT3 signalling and complement pathways, suggestive of an inflamed, immune-engaged state versus an immunologically cold epithelium (Fig. 2h).

### Immune-hot epithelia co-occur with immunoregulatory stroma

**Fig. 3:**
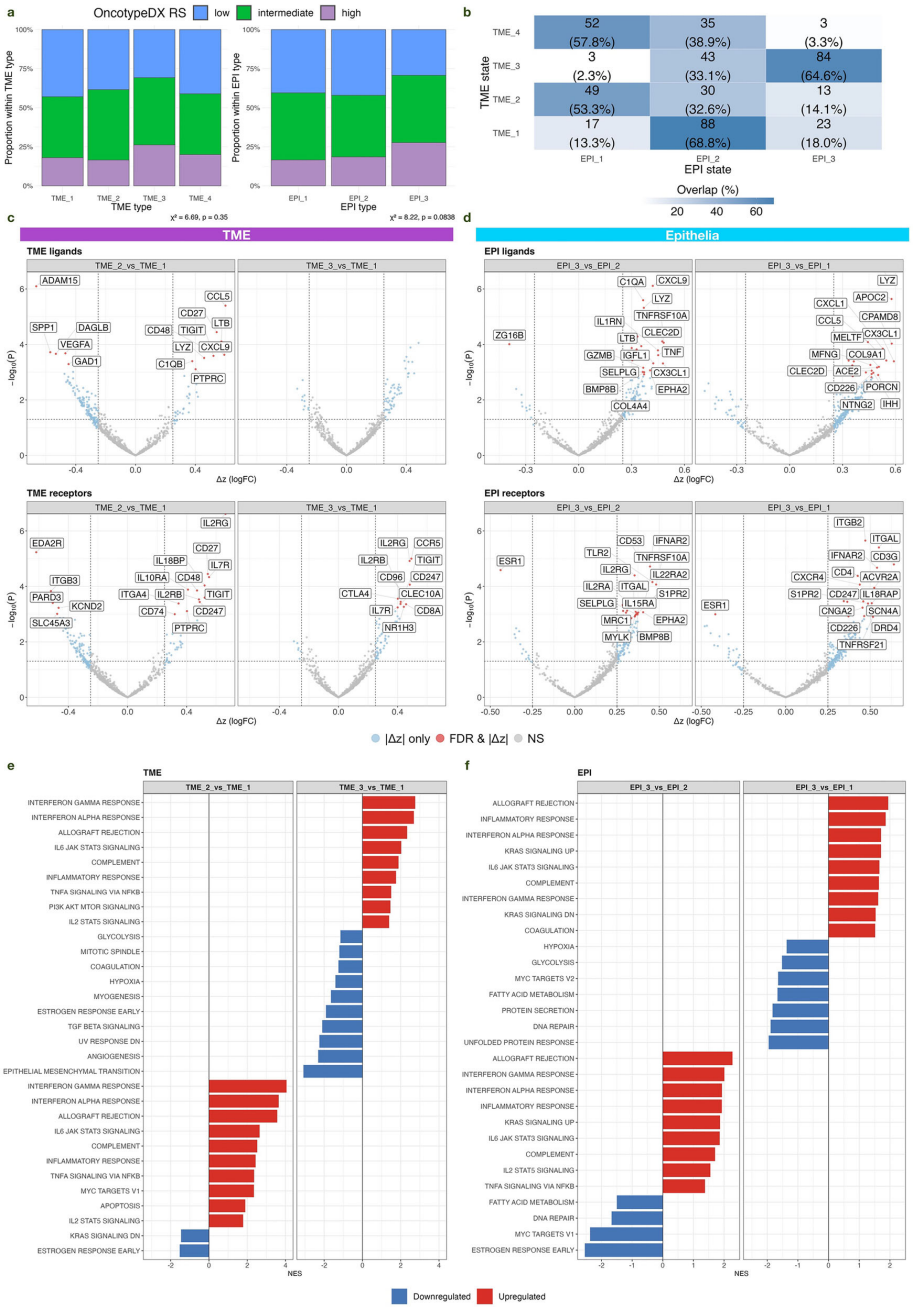
Outlining TME and EPI immune states. **a** Stacked barcharts of the proportion of each TME (tumour microenvironment) and EPI (epithelial) state across the Oncotype Dx RS. Bottommost text outlines the result of an omnibus Chi-squared test for the association of Oncotype Dx RS (recurrence score) categories across TME and EPI states. **b** Confusion matrix encoding the frequency and percentage of patient-level overlap between TME and EPI states. **c**, **d** Volcano plots of differentially expressed ligand-receptor genes derived from the OmniPath database. Δ-*z* is the difference in mean z-scored expression between clusters. Dashed lines denote Δ-*z* thresholds and p-thresholds derived from two-sided empirical Bayes moderated t-tests from Limma, encoded by blue point colour. Those passing adjustments for multiple comparisons with the Benjamini–Hochberg method are in red (FDR [false discovery rate] <0.05), for **c** TME, and **d** EPI states. Bar charts showing significantly up- and down-regulated hallmark pathways across **e** TME and **f** EPI states, adjusted for multiple comparisons with the Benjamini–Hochberg method FDR < 0.05. NES normalised enrichment score, FC fold change, NS non-significant.

We next investigated the prevalence of TME and EPI states across the cohort (proteomic data, *n* = 440). We found no significant enrichment of any state across categories of the Oncotype Dx RS (Fig. 3a, TME *χ*^2^ = 6.5, *p* = 0.37. EPI *χ*^2^ = 8.22, *p* = 0.084), implying that immune composition is not captured wholly by the genomic context. Subsequently, we explored the relationship between TME and EPI states. Immune-hot states in both TME (TME-3) and epithelia (EPI-3) tended to co-occur (Fig. 3b, *n* = 84, 64.6%). However, immune-cold epithelia (EPI-1, Fig. 3b) were observed in milieu exhibiting both a cold TME (Fig. 3b, TME-4: *n* = 52, 57.8%), and in milieu defined by T-cell infiltration/T-reg sparsity (Fig. 3b, TME-2: *n* = 49, 53.3%). These data suggest the presence of distinct immune-epithelial programmes that are partly, though not reliably, coupled. To investigate this further, using the matched spatial transcriptomic-proteomic dataset (*n* = 359), we subsequently employed cluster-wise differential expression (DEG) analysis of an OmniPath-curated^25^ ligand-receptor gene set, treating each spatial compartment independently. Comparing DEGs across each immune state (Fig .3c, d) revealed a significantly divergent transcriptomic landscape between states. While TME-2 (T-cell infiltrating) and TME-3 (immune-hot) both contain relatively higher densities of T-cells, TME-3 surprisingly exhibited no significant DEGs of ligands versus TME-1 (T-cell low), suggesting that the differences may be driven more by receptor programmes (Fig. 3c). TME-2, however, diverged from TME-1 in that ligand genes relating to angiogenesis (VEGFA), M2-like macrophage (SPP1), and extracellular matrix (ECM) remodelling (ADAM15) were significantly under-expressed, whereas ligand and receptor genes related to immune recruitment (CCL5, CXCL9), costimulation (CD27, CD48), activation (CD247, IL7R and IL2RB/G) and inhibition (TIGIT) were significantly overexpressed. These data are indicative of TME-2 being an immune-recruiting and activation-competent niche with concurrent inhibitory signalling. Comparatively to TME-1 (T-cell low), only receptor genes were overexpressed in TME-3 (immune-hot). These were related to immune trafficking (CCR5), effector presence (CD8A, CD247), cytokine response (IL7R, IL2RB/G) and checkpoints (CTLA4, TIGIT and CD96). Versus TME-2 (T-cell infiltrating, Supplementary Fig. 5), receptors relating to cell polarity/junction formation (PARD3), and ligands to ECM remodelling (ADAM15, SPINT1) and Wnt-antagonism (DKK1), were significantly overexpressed in TME-3 (immune-hot). Taken together, the TME of immune-warm states appear transcriptionally wired toward more adaptive lymphoid responses (TME-2) or immunoregulatory, stromal-remodelling niches (TME-3).

We also recognised patterns in the transcriptomic landscape of epithelia (Fig. 3d). EPI-3 (immune-hot) states significantly upregulated ligand genes related to chemokines and recruitment (CXCL9, CCL5 and CX3CL1), inflammatory regulation (TNF, IL1RN and C1QA) and cytotoxicity effectors (GZMB), indicating a more chemokine-active, inflamed interface versus both EPI-1 and EPI-2 states. In EPI-3, overexpressed receptor genes were likewise distinguished by immune trafficking (ITGAL, ITGB2 and CXCR4), interferon response (IFNAR2) and T-cell signalling (CD3G, CD247, IL2RA, IL2RG and IL15RA). Notably, in EPI-3 we also saw a consistent and significant underexpression of ESR1 (encoding ERα), suggesting that relatively immune-inflamed epithelia may be marked by both immune signalling, chemotaxis and de-differentiation from luminal-like biology. These data were further confirmed in GSEA analysis, wherein TME-3 (Fig. 3e) and EPI-3 (Fig. 3f) exhibited significant upregulation of inflammation-related, and downregulation of estrogen response early hallmarks. Immune-hot epithelia therefore appear as chemokine-active and IFN-responsive, with evidence of immune trafficking and signalling transcripts. Simultaneously, the inflamed stromal compartment is enriched for trafficking yet also checkpoints (CTLA4, TIGIT and CD96) and overexpresses additional remodelling-associated programmes. As TME-3 and EPI-3 are moderately coupled, this reveals a greater immune milieu where pathways related to immune activity and engagement within epithelia occur predominantly within a constrained, suppressive stromal environment.

We hypothesised that the presence of constrained stroma and chemokine-upregulated epithelia may provide useful prognostic information across the cohort. We subsequently explored the prognostic potential of immune states across genomic risk categories, using the proteomic data set (*n* = 440). We found no TME type or EPI type to be independently prognostic across any Oncotype Dx RS category overall (Supplementary Fig. 6). However, across randomised treatment arms of the Intermediate RS we found EPI-3 (immune-hot) to be an indicator of significantly poorer outcome on chemoendocrine therapy (Supplementary Fig. 6 HR: 4.09, 95% CI: 1.45–11.53, *p* = 0.008, FDR < 0.10). Interestingly, EPI-3 patients with an Intermediate RS trended to improved outcome when receiving randomised endocrine therapy alone (Supplementary Fig. 7 HR:0.092, 95% CI: 0.01–0.83, *p* = 0.032, FDR > 0.10).

### Cytotoxic T-cell density is a predictive marker in the Intermediate RS

Immune-hot states appear to circumscribe a potentially constrained, T-cell-dysfunctional stromal-immune hub (TME-3). This hub is moderately coupled to epithelia primed for immune chemotaxis and T-cell signalling (EPI-3), though we saw Intermediate RS patients with this EPI state had significantly poorer outcomes on chemotherapy. We next investigated whether any individual immune variable could recapitulate the finding of poorer outcome on chemotherapy seen in patients with an EPI-3 state. We therefore modelled continuous immune counts and densities across the Oncotype Dx RS, using the proteomic data set (*n* = 440). We found that an accumulation of CD8^+^ cells in epithelia trended toward poorer iDFS in the High RS (Supplementary Fig. 8. iTIL CD8% HR:1.53, 95% CI: 1.02–2.3, *p* = 0.041, FDR > 0.10). sTIL density of CD4^+^ and CD8^+^ cells, however, were both significantly prognostic (Supplementary Fig. 8. sTIL CD4^+^ density HR:1.04, 95% CI: 1.01–1.08, *p* = 0.010, FDR < 0.10. sTIL CD8^+^ density HR: 1.15, 95% CI: 1.02–1.29, *p* = 0.016, FDR < 0.10), suggesting that a greater T-cell infiltrate may parse tumours at greater risk of recurrence on chemotherapy. We also recognised trends to poorer prognosis in Low RS patients with high macrophage (CD68^+^) infiltrates (Supplementary Fig. 8. TME CD68% HR:1.08, 95% CI: 1.06–1.16, *p* = 0.032, FDR > 0.10), though we saw no prognostic trends in the Intermediate RS overall.

**Fig. 4:**
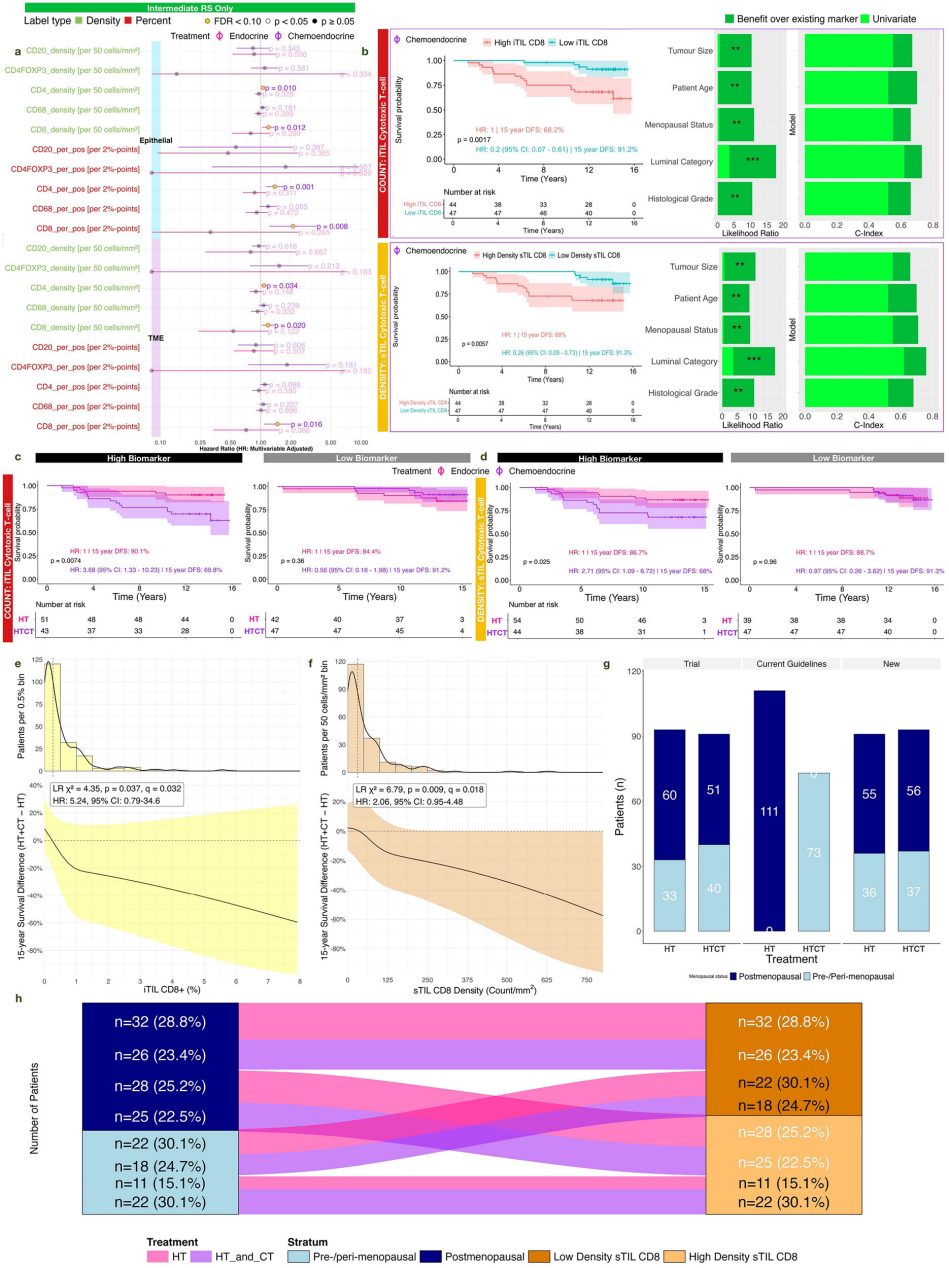
Immune phenotyping in the Intermediate RS*.* **a** Forest plot of multivariable Cox models across randomised treatment arms (HT *n* = 123, HT + CT *n* = 118), for immune densities/proportions (per step in the label) with clinical covariates (age, menopausal status, histological grade, tumour size and luminal status). Each row centre point encodes the hazard ratio (HR), and bars represent the 95% CI of the immune variable taken from multivariable models, independently of the other variables shown. White points denote *p* < 0.05, gold points denote variables passing adjustment for multiple comparison with a Bejamini–Hochberg FDR [false discovery rate] <0.10. **b** Kaplan–Meier curves for two exemplar biomarkers, dichotomised at the cohort median (left). Right panels show paired bar plots of model performance (ΔLR *χ*² [likelihood ratio-chi squared] and Δ*C*-index). Light green denotes the baseline clinical variable, dark green denotes bivariable models. Shaded regions show 95% CI (confidence interval). **c**, **d** Inverse-analysis Kaplan–Meier curves for the same biomarkers, stratified by received adjuvant treatment (HT = endocrine therapy, HT + CT = chemoendocrine). Annotations report the unadjusted Cox model HR with shaded regions representing the 95% CI and estimated 15-year invasive disease-free survival (iDFS) for each strata. **e**, **f** Predicted difference in 15-year iDFS (HT + CT − HT) from a spline-interaction Cox model as a function of the continuous biomarker (bottom), with a histogram and density curve of the biomarker distribution (percentage: bins 0.5%. Density: bins 50 cells/mm^2^) (top). Shaded regions show 95% CI. Curves below the horizontal line indicate poorer outcomes on chemoendocrine therapy. Adjusted interaction HR and 95% CI come from the rescaled Wald test values. *P*-values are derived from the likelihood-ratio test of interaction, *q*-values show FDR adjustment using the Benjamini–Hochberg method. **g** Stacked bar plots showing distributions of menopausal status and received treatment under trial criteria, current guidelines and the proposed new biomarker. Labels indicate patient counts. **h** Sankey diagram illustrating potential treatment reclassification from trial criteria to proposed biomarker, with flows coloured by treatment and biomarker strata.

However, for patients with an Intermediate RS, the underlying association of immune density with outcome could be modified and disguised by the type of treatment regimen administered. We therefore investigated by randomised treatment arm (Fig. 4). This analysis (Fig. 4a) revealed that patients receiving chemotherapy had significantly poorer 15-year iDFS if their tumours contained e.g. greater iTIL cytotoxic T-cell % (HR: 2.10, 95% CI: 1.22–1.3.64, *p* = 0.008, FDR < 0.10) or higher sTIL cytotoxic T-cell density (HR: 1.18, 95% CI: 1.03–1.37, *p* = 0.020, FDR < 0.10). This was likewise observed for sTIL T-helper cell density (HR: 1.08, 95% CI: 1.00–1.15, *p* = 0.034, FDR < 0.10) and iTIL T-helper cell density (HR: 1.19, 95% CI: 1.04–1.37, *p* = 0.012, FDR < 0.10). Using a median cutoff as an illustration for survival analysis, high sTIL CD8^+^ density (>median, ~30 cells/mm^2^) had significantly poorer survival than low density in the chemoendocrine arm (Fig. 4b. HR:0.26, *p* = 0.0057, 15-year iDFS high: 68% vs. 91.3% low), likewise for high iTIL CD8^+^ % (>median, 0.2%) (Fig. 4b HR:0.20 *p* = 0.0017, 15-year iDFS high: 68.2% vs. 91.2% low). Moreover, both high stromal density and epithelial count of cytotoxic T-cells provided significant additional prognostic information to nested models of clinical covariates (Fig. 4b). Investigating the prognostic power versus EPI states revealed that neither sTIL CD8^+^ density nor iTIL CD8% provided additional prognostic information to EPI states and vice versa (Supplementary Table 9). This importantly highlights substantial overlap in the prognostic signal found in EPI-3 and CD8^+^ density/percentage within the Intermediate RS. Together, these data underscore the prognostic utility of immune phenotyping above canonical risk variables in the intermediate RS and show that these data cannot be fully captured by spatially agnostic bulk phenotype scoring.

To further explore the possible differences in outcome by received treatment when scoring cytotoxic T-cells, we subsequently performed an inverse analysis (Fig. 4c, d). We divided the Intermediate RS into high vs low by median iTIL CD8% (Fig. 4c) and sTIL CD8^+^ density (Fig. 4d), and investigated the 15-year iDFS difference by received treatment. Low density CD8^+^ patient survival was not significantly different when receiving either endocrine therapy alone or additional chemotherapy (Fig. 4c, iTIL% *p* = 0.36. Figure 4d, sTIL density *p* = 0.96). Those with high iTIL CD8% (Fig. 4c) and high sTIL CD8^+^ density (Fig. 4d), however, had significantly poorer outcomes when receiving additional chemotherapy (Fig. 4c, iTIL CD8% *p* = 0.0074, 15-year iDFS HTCT: 69.8% vs. 90.1% HT-only. Figure 4D, sTIL CD8^+^ density *p* = 0.025, 15-year iDFS HTCT: 68% vs. 86.7% HT-only). Interaction plots (Fig. 4e, f), adjusted for clinical covariates, demonstrated that additional chemotherapy did not confer a significant benefit over endocrine therapy alone in our cohort when iTIL CD8% increased continuously (Fig. 4e, ΔLR *χ*²: 4.59, *p* = 0.032, *q* = 0.032. HR:4.35, 95% CI: 0.79–0.037). We saw this likewise with sTIL CD8^+^ density (Fig. 4f, ΔLR *χ*²: 7.03, *p* = 0.008, *q* = 0.016. HR:2.06, 95% CI: 0.95–4.48). Interaction plot curves shifted toward a poorer outcome on chemoendocrine therapy above median sTIL CD8^+^ density, though 95%CI excluding 1 was only seen beyond the ~70th percentile (~200 cells/mm^2^). The confidence intervals being wide across much of both biomarker ranges is likely due to the relatively small size of the Intermediate RS in our cohort. Thus, these results should be interpreted with caution and require external validation in a larger cohort of chemotherapy-randomised patients with an Intermediate RS before optimum thresholds can be generalised to practice.

As patients dropped out of analyses due to insufficient tissue, presence of artefactual cores, or withdrawal from the study, we next examined the proportion of clinical variables across dropout subsets, finding no significant difference in any strata (Supplementary Fig. 10). Secondly, as menopausal status is the current treatment stratifier for patients with an Intermediate RS (RS 16–25), we next wanted to examine the potential treatment change when using sTIL CD8^+^ density over menopausal status. Current clinical guidelines suggest that pre-/peri-menopausal patients may benefit from chemoendocrine therapy (RS 16–25), whereas postmenopausal women would not significantly benefit (RS 0–25) (Fig. 4g: current guidelines). Since our cohort was derived from patients previously enrolled in TAILORx, before these guidelines were updated, a large number of pre- and post-menopausal patients received either therapy (Fig. 4g: trial). Using high sTIL CD8^+^ density (>median) as a predictor of response, we see a potential treatment change of 50% (*n* = 56/111) of postmenopausal women (RS 16–25) from endocrine therapy alone to chemoendocrine therapy. Of pre-/peri-menopausal women (RS 16–25), 49% (*n* = 36/73) would have a change in treatment from chemoendocrine therapy to endocrine therapy alone. While the distribution is similar to the working trial cohort (Fig. 4g: trial, new), the Sankey diagram (Fig. 4h) demonstrates that a significant proportion of those patients would experience a change in treatment despite a similar split in menopausal status within treatment strata to the trial distribution. Moreover, testing this effect across subsets of menopausal status and randomised treatment groups, we found high sTIL CD8^+^ density was only statistically significantly prognostic in postmenopausal patients (Supplementary Fig. 11, premenopausal: low sTIL CD8 density HR:0.27, 95% CI: 0.05–1.41, *p* = 0.097, postmenopausal: low sTIL CD8 density HR:0.27, 95% CI: 0.07–1.0, *p* = 0.035), likely due to few reduced events after stratification in the premenopausal group, as the directionality of the effect was consistent across menopausal statuses.

### Internal orthogonal validation on whole tissue resections

**Fig. 5:**
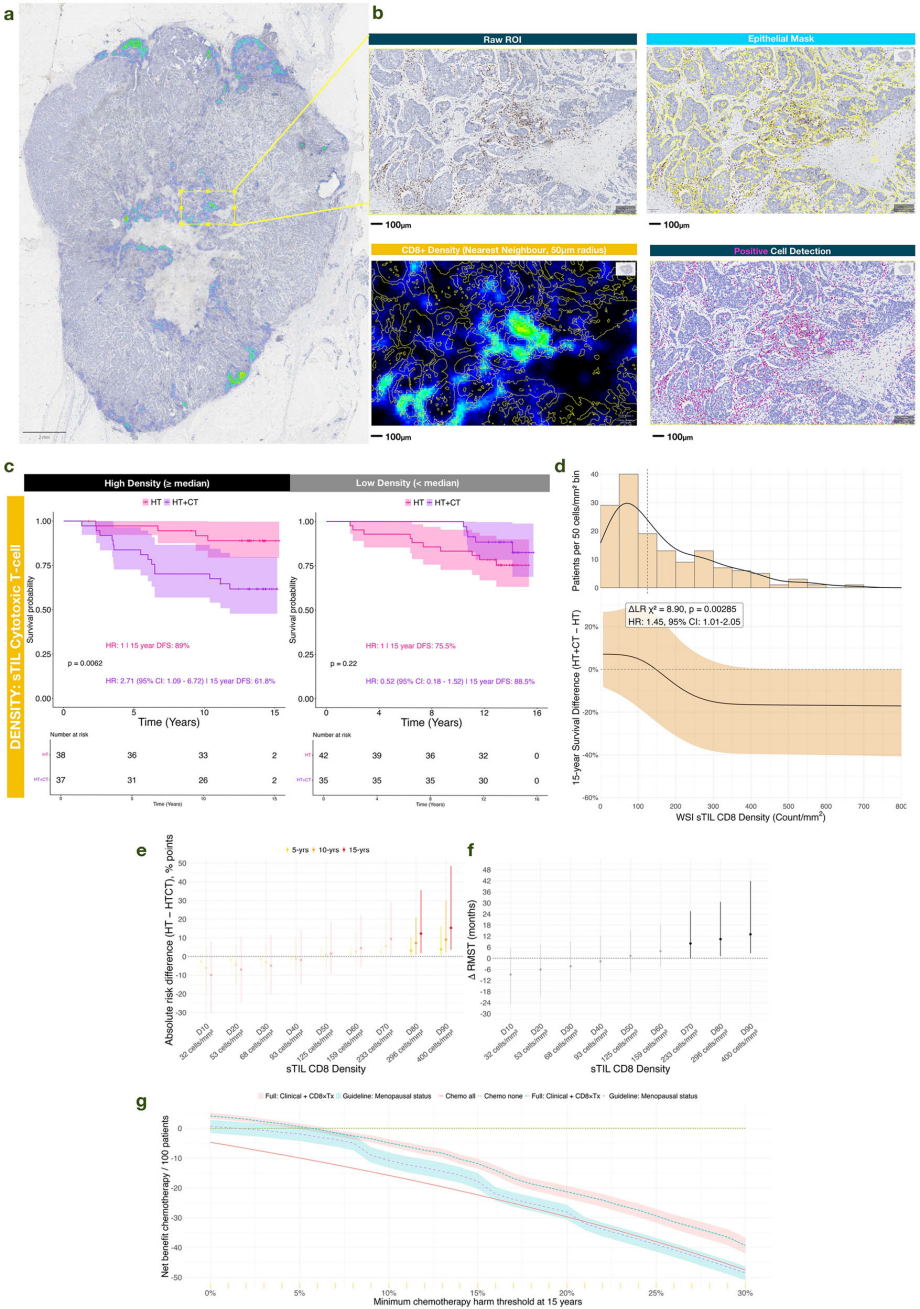
Orthogonal validation of the proposed biomarker. **a** Example CD8-IHC WSI (whole-slide image), from the CD8 WSI dataset (*n* = 437). Scale bar shows 2 mm. Yellow inset shows a region of interest (ROI). **b** ROI panels: raw image (top left), positive cell detection mask (top right), epithelia segmentation mask (bottom left), and CD8 density by nearest-neighbour (NN) of 50 µm (bottom right). Scale bars show 100 µm. **c** Kaplan–Meier curves of stromal CD8 density stratified by received adjuvant therapy (HT = endocrine therapy, HT + CT = chemoendocrine therapy) and dichotomised at the cohort median. Shaded regions show 95% CI. Survival curves are unadjusted. **d** Predicted difference in 15-year invasive disease-free survival (iDFS) (HT + CT − HT) from the spline-interaction Cox model as a function of continuous stromal CD8 density (bottom), adjusted for all clinical covariates (age, menopausal status, histological grade, tumour size, luminal status), with a histogram and density curve of the biomarker distribution (top). Shaded areas correspond to 95% confidence interval (CI). *P*-values are taken from the likelihood ratio test of interaction (ΔLR *χ*²). Adjusted interaction hazard ratio (HR) and 95% CI come from the rescaled Wald test values. **e** Absolute survival difference (HT − HT + CT) for biomarker deciles (**d**) across 5, 10 and 15 years (point colour encodes the time horizon). Points encode the survival difference, error bars show 95% CI (bootstrap, 1000 resamples). Colour opacity shows significance. HT *n* = 80, HT + CT *n* = 72. **f** Difference in restricted mean survival time at 15 years (Δ-RMST, HT − HT + CT) at the same biomarker percentiles. Points encode mean survival difference, error bars show 95% CI (bootstrap, 1000 resamples). Colour opacity shows significance. HT *n* = 80, HT + CT *n* = 72. **g** Decision curve analysis comparing treatment policies across minimum chemotherapy harm thresholds. Curves show standardised benefit (higher is better). Teal lines show the proposed biomarker policy, purple lines show the menopausal status policy. Red and green lines show treat-all (red) versus treat-none (green) policies. Shaded areas show 95% CI (bootstrap, 1000 iterations). For interpretation, at a chosen threshold (e.g. 5%), the vertical distance between curves is the gain in net benefit, approximating the additional correct treatment decisions per 100 patients compared with the alternative policies.

To mitigate potential TMA sampling bias and in lieu of an accessible validation cohort with similar 1:1 randomisation of the Intermediate RS, we next performed an internal orthogonal validation of cytotoxic T-cell density on whole resection specimens (WSIs) only within the randomised Intermediate RS group (*n* = 151) (Fig. 5a, b). Using an illustrative cut-off of median sTIL CD8^+^ density (median ~125 cells/mm^2^) showed significantly poorer iDFS of patients receiving additional chemoendocrine therapy (*p* = 0.0062, 15-year iDFS HTCT: 61.8% vs. 89% HT-only, Fig. 5c). We crucially discovered a treatment-biomarker interaction, indicating poorer outcomes of Intermediate RS patients prescribed additional chemotherapy as sTIL CD8^+^ density increased (adjusted for clinical covariates. Up to −18% survival difference at 15-years. ΔLR-*χ*^2^: 8.90, *p* = 0.0029. HR:1.45, 95% CI: 1.01–2.05) (Fig. 5d). Absolute survival differences (ASDs) across prespecified percentiles (10th–90th, Fig. 5e) showed a monotonic trend toward no benefit from chemotherapy with high (>50th percentile) WSI sTIL CD8^+^ density. At the 90th percentile of density, a 3.9% (95 CI: 0.6–15.8%, *p* = 0.008, FDR < 0.10), 9.0% (95% CI: 2.23–29.9%, *p* = 0.006, FDR < 0.10) and 15.3% (95% CI: 3.66–48.5%, *p* = 0.006, FDR < 0.10) higher iDFS was observed at 5 years, 10 years and 15 years, respectively, for patients receiving endocrine therapy only (Fig. 5e).

Over a 15-year horizon, the delta in restricted mean survival time was up to 12 months in favour of endocrine therapy-only (90th percentile, 95% CI: 2.16–37.4, *p* = 0.008, FDR < 0.10. Fig. 5F). Decision curve analysis demonstrated that sTIL CD8^+^ density provided higher net benefit than menopausal status across most thresholds. Particularly 0–3% and 8–30% (bootstrap *p* = 0.004, FDR < 0.10, *p* = 0.006, FDR < 0.10, respectively). To contextualise, a harm threshold of 15% indicates an intention to treat when the probability of recurrence is at least 15%, otherwise spare chemotherapy. Using sTIL CD8^+^ density at this threshold yielded a net benefit corresponding to 6 more patients per 100 correctly managed over the 15-year period compared with menopausal status (95% CI: 2.65–8.62, *p* = 0.002, FDR < 0.10). Compared with a chemo-for-all policy, sTIL CD8^+^ density yielded 11 more per 100 correctly managed (95% CI: 7.25–14.19, *p* = 0.002, FDR < 0.10). While these results require external validation to increase discriminatory power at lower percentiles, our data suggest that sTIL CD8^+^ density is a clinically translatable predictive biomarker that may help identify Intermediate RS patients (RS 16–25) more likely to be harmed by, or less likely to benefit from, additive chemotherapy regimens. Crucially, this data is independent of menopausal status. Though these data require extensive external validation, our data suggest this could result in up to 50% of all Intermediate RS patients (RS 16–25) experiencing a change in adjuvant treatment from the existing paradigm (49% pre-/peri-menopausal de-escalated from, and 50% postmenopausal escalated to, chemotherapy).

### Spatial and transcriptomic signatures link immune states to exclusion, checkpoint activity and T-cell exhaustion

**Fig. 6:**
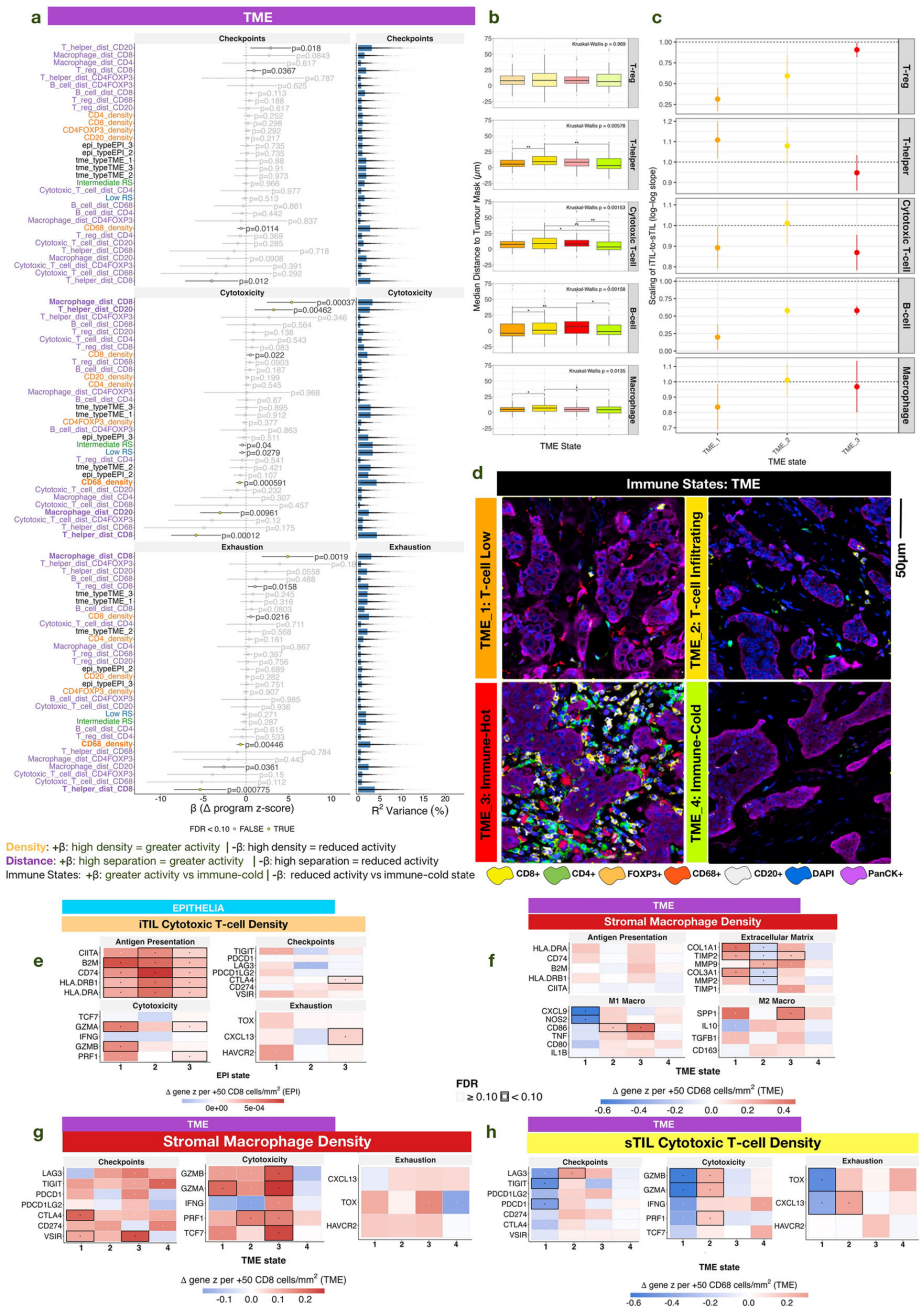
Spatial-immune context and gene readouts. **a** Forest plot of regression coefficients for predicting gene module scores within TME ([tumour microenvironment] left, patient *n* = 359), with bar charts of partial *R*^2^ variance (i.e. proportion of variance explained, right. Error bars show 95% confidence interval [CI] from 5000 bootstrapped resamples. Gene modules are defined as the mean expression of constituent genes in the TME/stroma: cytotoxic = GZMA, GZMB, PRF1, IFNG. Checkpoints = TIGIT, LAG3, CTLA4, CD274, PDCD1, PDCD1LG2, VSIR. Exhaustion = TOX, CXCL13, HAVCR2. All predictors were z-scored. Models include stromal immune cell densities and proximity metrics, with TME/EPI (epithelial) state and Oncotype Dx RS as covariates, and were adjusted for variables except the predictor of interest. Points show the estimated regression coefficient with error bars denoting the 95% CI. White points indicate *p* < 0.05, gold points and bold labels denote FDR (false discovery rate) <0.10. Raw *p*-values correspond to type II ANOVA tests for each term in the model. **b** Boxplots showing phenotype distances to a tumour mask (µm), stratified by TME states 1–4. Patient TME-1 *n* = 161, TME-2 *n* = 100, TME-3 *n* = 125, TME-4 *n* = 51. Box plots show the median (centre line), with the box spanning the interquartile range (25th–75th percentiles). Whiskers extend to the most extreme values within 1.5 × the interquartile range from the box. Outliers beyond the whiskers were not displayed. Asterisks denote *p*-values of two-sided Dunn’s test for pairwise comparisons. Opacity encodes significant differences. Significance is as follows: *p* < 0.05 (*), 0.01 ≥ *p* > 0.001 (**), *p* ≤ 0.001 (***). Exact *p*-values are: T-helper cell TME-1 vs. TME-2 *p* = 0.00747, TME-2 vs. TME-4 *p* = 0.00747. Cytotoxic T-cell TME-1 vs. TME-4 *p* = 0.031, TME-2 vs. TME-4 *p* = 0.00162, TME-3 vs. TME-4 *p* = 0.00162. Macrophage TME-1 vs. TME-2 *p* = 0.00747, TME-2 vs. TME-4 *p* = 0.0747. B-cells TME-1 vs. TME-2 *p* = 0.0398, TME-1 vs. TME-3 *p* = 0.00131, TME-3 vs. TME-4 *p* = 0.0398. **c** Dot and whisker plots showing stromal-to-epithelial coupling of immune infiltration across TME states. Dotted line indicates proportional coupling. TME-4 was dropped, as the relative lack of immune cellularity resulted in unstable slope estimates. **d** Illustrative examples of immune infiltrate in TME states, from spatial proteomics analyses, showing PanCK+ tumour epithelium (magenta), CD68+ macrophages (red), CD4 + FOXP3+ T-regulatory cells (green-orange), CD8^+^ cytotoxic T-cells (yellow), CD4+ T-helper cells (green), CD20^+^ B-cells (white). **e–h** Heatmaps of regression slopes for immune module genes by cell phenotype density (Δ-gene z-score, i.e. for each gene, how much does its expression change as the predictor variable density increases by 50 cells/mm^2^, adjusting for other stromal/epithelial-immune densities). Black outlines show genes passing a discovery FDR < 0.10. **e** Increasing epithelial (iTIL) cytotoxic T-cell density. **f**, **g** Increasing TME (stromal) macrophage density. **h** Increasing TME (sTIL) cytotoxic T-cell density.

Having previously defined discrete immune ecologies in the TME and epithelium, we next wanted to better understand the transcriptomic and spatial landscape accompanying EPI and TME states, and whether these are associated with features that could plausibly influence adjuvant chemotherapy benefit. Using the matched proteomic-transcriptomic dataset (*n* = 359), we firstly modelled transcriptional immune programmes as a function of immune densities, spatial proximities and immune states, by fitting regression models with module scores as outcomes. This was performed for cytotoxicity (GZMA, GZMB, IFNG, PRF1 and TCF7), checkpoints (TIGIT, PDCD1, LAG3, PDCD1LG2, CTLA4, CD274 and VSIR) and exhaustion (TOX, CXCL13 and HAVCR2) modules, to find which variable most significantly contributed to each gene programme (Fig. 6a).

We found TME and EPI states to not be significantly associated with any immune programme, though trends of increased cytotoxicity and exhaustion in TME, and checkpoints in EPI states, were seen in all but the immune-cold (i.e. TME-4, EPI-1) state (Fig. 6a). On the contrary, we found that increasing stromal macrophage density (+1 SD, ~220 cells/mm^2^) showed a significant negative trend for exhaustion module (*β* = −0.65, 95% CI: −1.08 to −0.21, *p* = 0.0045, FDR < 0.10), and cytotoxicity module genes (*β* = −0.74, 95% CI: −1.16 to −0.33, *p* < 0.0001, FDR < 0.10), with trends observed in the checkpoint module (*β* = −0.58, 95% CI: −1.02 to −0.13, *p* = 0.011, FDR > 0.10). This negative association is consistent with an immunomodulatory macrophage context. Moreover, spatial proximity of cell phenotypes revealed that T-helper and macrophage distances, particularly from B-cells (CD20^+^) and cytotoxic T-cells (CD8^+^), provided additional information on gene module activity to densities and TME/EPI states. As example, increasing macrophage separation from cytotoxic T-cells was associated with increased cytotoxicity (Fig. 6a. *β* = 5.34, 95% CI: 2.46–8.22, *p* < 0.001, FDR < 0.10) and exhaustion module activity (Fig. 6a, *β* = 4.90, 95% CI: 1.85–7.93, *p* < 0.001, FDR < 0.10), again consistent with an immunomodulatory role of macrophages across our cohort. Similarly, increasing T-helper cell separation from cytotoxic T-cells was associated with a marked decrease in cytotoxicity module activity (Fig.6A, *β* = −5.87, 95% CI: −8.78 to −2.96, *p* < 0.001, FDR < 0.10), but also in the exhaustion module (Fig. 6A, *β* = −5.37, 95% CI: −8.44 to −2.30, *p* < 0.001, FDR < 0.10), a finding that is compatible with an immune-engaged niche wherein activation and chronic stimulation co-occur. These data suggest that immune density and spatial organisation provide granularity beyond TME/EPI states in explaining the immune transcriptional programmes active within stromal regions.

To explore spatial organisation further, we computed at the patient level the signed distance of each cell from an epithelial (PanCK^+^) tumour mask in samples stratified by their assigned TME state (negative distances indicate cells within a tumour mask). We observed systematic differences across TME states for all phenotypes except T-regs (Fig. 6b, T-reg Kruskal–Wallis *p* = 0.989, other *p* < 0.01). For macrophages, cytotoxic T-cells and T-helper cells, TME-2 (T-cell infiltrating) tended to exhibit the largest median distance from a tumour boundary than other TME states (Fig. 6b), whereas in TME-3 (immune-hot) and TME-1 (T-cell low), the distances of T-helper cells and macrophages were not significantly different from immune-cold (TME-4) tumours (Fig. 6b). We next investigated the relationship between iTILs and sTILs across all phenotypes and TME states, by modelling iTIL densities as a function of sTIL densities. Across cores, increases in sTIL densities were expectedly strongly associated with increases in iTIL densities (logStroma *χ*^2^ = 3063.97, *p* < 0.0001). However, this relationship differed by TME state (2-way interaction TME state:logStroma: *χ*^2^ = 14.59, *p* = 0.0007), and was further dependent on cell phenotype (3-way interaction TME state:phenotype:logStroma: *χ*^2^ = 69.45, *p* < 0.0001). For each phenotype and TME state, we subsequently plotted the log-log slope (Fig. 6c). This plot shows the relationship in density between iTILs and sTILs as sTIL densities increase: values < 1 indicate that an accrual of sTILs does not translate proportionally to an accrual of iTILs, therefore suggestive of restricted epithelial entry. Patients with TME-2 (T-cell infiltrating) exhibited the highest scaling across all phenotypes, suggesting a greater ability of accruing sTILs to penetrate the epithelia (Fig. 6c). This was most pronounced in T-helper cells (*β* = 1.08, 95% CI: 0.99–1.17) and macrophages (*β*:1.01, 95% CI: 0.90–1.12). In immune-hot TME-3, however, stromal-epithelial scaling was consistently sub-proportional (*β* < 1), the most strongly coupled phenotype being T-regulatory cells (*β* = 0.91, 95% CI: 0.82–0.99). We also observed the lowest scaling relationship in TME-3 of T-helper cells (*β* = 0.95, 95% CI: 0.86–1.03), and cytotoxic T-cells (*β* = 0.87, 95% CI: 0.78–0.95) were similarly coupled to TME-1 (T-cell low), suggesting greater similarities in the spatial organisation of TILs between T-cell low (TME-1) and immune-hot (TME-3) states than between immune-hot and T-cell infiltrating milieu (TME-2). Taken together, our data suggest an enrichment of peri-tumoural TILs across TME states, wherein immune-hot and T-cell low environments exhibit more pronounced restriction in epithelial entry of immune infiltrates than those with intermediary infiltration of TILs (Fig. 6d).

To understand whether an increasing density of immune infiltrates could be associated with increasing or decreasing immune responses and exclusionary mechanisms, we next performed a regression analysis for macrophage and cytotoxic T-cell density (steps of 50 cells/mm^2^), against individual genes used in previous gene modules (Fig. 6e–h), as these density variables were most frequently associated with gene module activity outlined previously (Fig. 6a). In EPI-1 (immune-cold) states, increasing iTIL CD8^+^ density was associated with significantly upregulated antigen presentation (B2M, CD74, HLA.DRB1 and HLA.DRA), and cytotoxic effector genes (GZMA, GZMB), indicating that, if CD8^+^ cells infiltrate, they are more likely to reflect active effector engagement in this state. EPI-2 (intermediate infiltrate) was associated with the most significant upregulation of antigen presentation genes, yet cytotoxicity genes were not significantly coupled, implying that immune pressure is not consistently translated to effector killing in this state. EPI-3 (immune-hot) states were likewise associated with strong antigen presentation gene upregulation (CIITA, B2M, CD74, HLA.DRB1 and HLA.DRA), though weaker cytotoxicity (GZMA, PRF1), and significant upregulation of CTLA4 and CXCL13. EPI-3, therefore, likely reflects an environment with adaptive inhibition and chronic stimulation^26^, further implicating the potential presence of a dysfunctional and constrained anti-tumour immune response.

Within the stroma, increasing macrophage density (Fig. 6f, g) was not coupled to antigen presentation across any TME state (Fig. 6f). In TME-1 (T-cell low), macrophage density was markedly associated with extracellular matrix and fibrosis-associated gene upregulation (COL1A1, COL3A1 and TIMP2), reduced M1-like features (CXCL9, NOS2), and global trends to downregulation in checkpoints (significantly TIGIT and PDCD1 – encoding PD-1), cytotoxicity (significantly GZMA, GZMB), and exhaustion genes (significantly TOX, CXCL13). Increasing macrophage density in state TME-2 (T-cell infiltrating) indicated a more permissive stroma. ECM/fibrosis genes were frequently significantly downregulated (COL1A1, COL3A1, TIMP2 and MMP2), coupled to significant upregulation of cytotoxicity module genes (GZMA, GZMB and PRF1), indicative of more canonical adaptive inflammation. Increasing macrophage density in TME-3 (immune-hot) was, however, associated with M2-like (SPP1) and ECM regulation features (TIMP2; trends in MMP9), and a trend of downregulated cytotoxicity genes. TIMP2 is an inhibitor of matrix metalloproteinases in the TME^27^, implying that TME-3 stroma exhibits strict control over ECM degradation, and may be more functionally constrained in its immune response by SPP1^+^ macrophages^28^. These data mirror our spatial findings that state TME-1 and TME-3 have significantly reduced sTIL-iTIL coupling compared with TME-2.

We subsequently investigated changes in cytotoxicity, checkpoints and exhaustion module genes as sTIL CD8^+^ density increased across TME states. TME-3 (immune-hot) exhibited significant upregulation of cytotoxicity genes (GZMA, GZMB and PRF1), indicating enhanced killing potential; however, it also significantly upregulated TCF7 (encoding TCF-1, a CD8^+^ cell transcriptional regulator that maintains a progenitor-exhaustion state^29^). This suggests the presence of T-exhausted cells with progenitor-like capacity, which are known to be crucial in an optimal response to ICB^30^. In this vein, TME-3 milieu were also coupled to trending increases in most checkpoints (LAG3, TIGIT, PDCD1, PDCD1LG2 and CTLA4). We also recognised significant upregulation of VSIR (encoding VISTA), an emerging immune checkpoint shown to suppress T-cell immune responses^31,32^, the expression of which strongly associates with SPP1^+^ macrophages, and ICB resistance, in colorectal cancer^33^.

Altogether, our analyses support a model in early-stage ER^+^HER2^−^ disease in which inflamed microenvironments exhibit peri-tumoural immune enrichment with relatively excluded epithelia. This arrangement coincides with transcriptional features consistent with adaptive inhibitory signalling, chronic stimulation and ECM remodelling. While these observational data cannot establish causality, we hypothesise that in immune-dense tumours with engaged stromal remodelling and immunoregulatory programmes, the addition of chemotherapy could amplify existing wound-healing and suppressive pathways rather than enhance effective tumour cell killing. This may help to explain the observed pattern in which patients with higher densities of sTIL cytotoxic T-cells have poorer outcomes on chemoendocrine therapy: the existing infiltrate may not be fully capable of responding to and clearing the residual cancer, as the microenvironment may be promoting a dysfunctional/exhausted milieu.

## Discussion

Here, we have demonstrated that immune phenotyping is significantly prognostic in early-stage ER^+^HER2^−^ disease, but heavily dependent on the underlying genomic risk of the tumour. In whole-cohort assessments agnostic of genomic risk, TIL analysis produced results in agreement with the consensus view of mostly low immunogenicity, poor prognostic dichotomisation, and weak, if no, ability to predict outcome in ER^+^HER2^−^ breast cancer overall. However, using multiplex immunofluorescence-based spatial proteomic data, we have detailed how epithelial and TME states exist on a spectrum of immune-hot to immune cold. While these states were not prognostic overall, we showed that immune-hot epithelia confer adverse outcome in Intermediate RS patients receiving chemoendocrine therapy, with trends to improved outcome on endocrine therapy alone. These epithelia are associated with increased chemokine-cytokine signalling, T-cell signalling and downregulated ESR1 (encoding ERα). They are furthermore partially coupled to stromal states (TME-3), indicative of fibrosis/wound-healing and repair, chronic immune stimulation and expression of checkpoint programmes. Similarly, high densities of T-cells in stroma (T-helper and T-cytotoxic) were independently prognostic across the Intermediate RS receiving chemoendocrine therapy, and the High RS. Crucially, using randomised treatment in the Intermediate RS, we discovered that sTIL CD8^+^ density is a predictive marker of poorer outcomes on chemotherapy across TMA sections and demonstrated potential generalisability of the biomarker via orthogonal, whole-resection specimen validation. The predictive information was independent of all clinical covariates, and suggests potential treatment changes for up to 50% of all patients with an Intermediate RS (RS 16–25) that requires comprehensive external validation.

We first outlined how the immune milieu of our cohort differs. We found only one truly immune cold TME and epithelia, whereas the others ranged from immune-infiltrated to immune-hot. Cluster-wise analysis of a curated ligand-receptor gene set^25^ highlighted a range of transcriptionally active stromal environments, with TME-2 (T-cell infiltrating) indicating a more classical inflammatory response than TME-3 (immune-hot). TME-3 was dominated by chronic inflammation, ECM remodelling and checkpoint signalling (CTLA4, TIGIT). In this vein, relatively inflamed EPI states (i.e. EPI-3) were primed for immune chemotaxis, contact and T-cell signalling, and were largely coupled to immune-hot stroma (TME-3), whereas cold epithelia (EPI-1) existed within both cold and T-cell infiltrating stroma (TME-4, TME-2, respectively). We outlined how increasing densities of iTIL cytotoxic T-cells were significantly positively associated with antigen presentation (MHC-I and MHC-II) across EPI states, with increasing checkpoint expression (CTLA4), and exhaustion genes (CXCL13) in immune-hot epithelia. These epithelial profiles parallel exhaustion-associated luminal breast cancer immune environments derived from single-cell spatial data recently^26^. Moreover, while enhanced antigen presentation by epithelia can also promote T-cell recruitment, paracrine IFN-γ secretion by T-cells in neighbouring immune-hot but suppressed peri-tumoural niches can induce MHC-II-like responses in epithelial cells^34^, sustaining chronic stimulation without effective cytotoxic programme activity. This is known to promote T-cell exhaustion-like phenotypes rather than those capable of effective tumour cell clearance^4^, and mirrors our data as increasing iTIL CD8^+^ density associated with both increased cytotoxicity-related but also exhaustion-related gene expression.

Across TME states, we recognised that macrophages were the most frequent phenotype and often associated with decreased immune activity, independently of TME and EPI states. Increasing macrophage density in TME states was not significantly associated with MHC-I-like nor MHC-II-like antigen presentation genes, though M2-like gene SPP1 was significantly upregulated in immune-hot stroma. Single-cell omics of luminal breast cancers has recently shown that suppressive myeloid niches can attract T-cells, denoting an exhausted cluster with altered but not abolished cytotoxicity, that corresponds with spatial hubs of macrophage/T-helper/T-reg/cytotoxic T-cells, and enhanced matrix metalloproteinase expression^26^. Furthermore, a recent study by Cha et al. has shown in HR^+^ breast cancer an increased propensity of SPP1^+^ macrophage to interact with T-helper and cytotoxic T-cells, most notable in TIL-high tumours, in which SPP1^+^ macrophage inhibits T-cell responses to tumour through regulatory signalling and ECM formation^28^. A principal immunosuppressive role of SPP1^+^ macrophage has also been observed in ovarian cancer^35^, and fibrosis promotion via ECM remodelling by macrophage has been linked in breast cancer cell lines to dysfunctional CD8^+^ T-cell responses, independent of checkpoint signalling, by both physical exclusion and metabolic reprogramming that impedes antitumour immunity^36^. Indeed, we further recognised that increasing macrophage density in T-cell low (TME-1) and immune-hot (TME-3) states is accompanied by a downregulation in checkpoints, cytotoxicity and exhaustion genes, underscoring a potentially more suppressive or exclusionary macrophage plasticity that may promote immune exclusion and T-cell dysfunction.

A recent study suggests Intermediate RS tumours contain exhausted T-cells with a negative prognostic influence^37^, and that checkpoint proteins, such as PD-L1, are positively associated with Oncotype Dx RS^38^. More recently, larger studies have begun to demonstrate PD-L2 may actually be a more granular predictor of early relapse risk in ER^+^HER2^−^ breast cancer^39^, an independent variable in multivariable analysis of those patients treated with chemoendocrine therapy. Our data suggest that, independently of the Oncotype Dx RS, ostensibly exhausted T-cells and immunosuppressive environments are associated largely with inflamed TME and epithelia. Subsequent modelling across the Oncotype Dx RS demonstrated that patients with T-cell-high tumour-immune milieu have significantly poorer outcomes on chemotherapy (Intermediate and High RS). While paradoxical, it is a rationale for continued exploration of immune checkpoint blockade (ICB) in early-stage ER^+^HER2^-^ breast cancer. This is of particular import in Intermediate RS and High RS patients, where reliance on ERα and estrogen-mediated signalling is decreasing, particularly as we recognised significant underexpression of ESR1 in the immune-hot EPI-3 states. The successes of recent trials with immune checkpoint blockade in the arena of high-risk, later stage ER^+^ disease (Checkmate 7FL:^8^, KEYNOTE 756:^10,40^, I-SPY2^41,42^:) show that there is a benefit to ICB in less ER-reliant environments (e.g. Checkmate ER <50%). Our data from GSEA analyses furthermore show that those patients for whom we hypothesise ICB may be most beneficial upregulate interferon (alpha and gamma) responses, allograft rejection and IL6-JAK-STAT3 signalling pathways in the stroma and epithelium, and downregulate early estrogen responses and DNA repair. These pathways have very recently been shown to be upregulated in patients who significantly benefitted from pembrolizumab + taxane chemotherapy in a pilot study of early-stage ER^+^HER2^−^ breast cancer^43^. Further work is required to investigate, with proteomics analyses, the potential presence and spatial colocalisation of checkpoints beyond the transcriptome, and their relationship to the Oncotype Dx RS, for potential patient selection to immunotherapies.

We lastly, and most crucially, show that cytotoxic T-cell density is a predictive marker in patients with an Intermediate RS. While our data provide strong evidence of poorer outcomes of patients on additive chemotherapy, it is not yet known whether patients require de-escalation or combination treatment with ICB. On the one hand, chemotherapy administration in early-stage ER^+^HER2^-^ disease may ablate existing adaptive responses by eradicating the high-avidity/affinity T-cells that are responsible for the proliferative burst in these populations post-treatment^44,45^. On the other, the administration of chemotherapy and subsequent treatment-induced stimulation of damage-associated molecular patterns will be dampened by a TME exhibiting increased immunosuppressive signalling pre-treatment, thereby hindering the induction of immunogenic cell death^46,47^. The administration of chemotherapy has also been shown to promote fibrotic responses through existing wound-healing myeloid and fibroblast cells in triple-negative breast cancer, promoting T-cell dysfunction that supports metastatic recurrence^48^.

It is a shortcoming of our study that we have no external validation data. Prospective clinical cohorts with treatment randomisation of the intermediate RS are very sparse. As such, for clinical adoption, it may be necessary to validate these findings in the larger TAILORx trial. A comprehensive validation is required before clinical adoption of the proposed biomarker—and the ramifications of treatment change for a marked population of patients with an intermediate RS—can be weighed and seriously discussed. Without this, optimal thresholds cannot be established, which will be required to precisely understand whether and for whom chemotherapy is truly inferior for the biomarker population.

## Methods

### Ethics statement

Ethical approval for the study was provided by the institutional ethics committees of 11 Cancer Trials Ireland (CTI)-affiliated hospitals, along with the Ethics Committee of Beaumont Hospital, the Royal College of Surgeons in Ireland (Tallagh Hospital; St James’s Hospital; Letterkenny General Hospital: REC 2014 56/2014/03; REC 2016-07 List 25^4^. Beaumont Hospital: REC 13/81, Cork University Hospital; Bon Secours Hospital: ECM 3 (ttt) 07/01/14; ECM 4 (ddd) 03/12/13 & ECM 3 (yyyyy) 21/06/16., Galway University Hospital 17/12. Mater Misericordiae University Hospital; Mater Private Hospital: 1/378/1590., Sligo Regional Hospital; St Vincent’s University Hospital; University Hospital Limerick; Waterford Regional Hospital: n/a). Written informed consent was provided by patients enrolled in the prior TAILORx trial and included consent for the use of data and biological samples for future studies.

### Study design and TMA

The TAILORx Tissue Bank (CTRIAL-IE 12-30, NCT02050750) is an exploratory, translational, non-interventional multicentre biobank sponsored by Cancer Trials Ireland that aims to identify potential biomarkers. Eligibility required prior registration with the TAILORx trial (CTRIAL-IE (ICORG) 06-31, NCT00310180), participation in trial arms and having sufficient tumour material available for immunohistochemical staining. Other than the accrual of female patients who were also accrued to the Eastern Cooperative Oncology Group trial TAILORx, there was no connection between the two studies, and the analysis performed on the patient samples from CTRIAL-IE 12-30 did not impinge in any way on the TAILORx trial. Under the CTRIAL-IE 12-30 study protocol, formalin-fixed paraffin-embedded (FFPE) tissue blocks of the identified eligible patients were retrieved from pathology archives and shipped to the study biobank. RS values were taken as being 0–15 for low, 16–25 for intermediate and 26–100 for high, following from the recommendations of the TAILORx studies^49,50^. Cohort characteristics can be found in Table 1. The primary end-point of this study was invasive disease-free survival (iDFS), as per the STEEP criteria^51^, defined as the first invasive recurrence (distant, ipsilateral, locoregional), second primary invasive cancers, or death from any cause. There had been 108 iDFS events over a median follow-up of 158 months (SD ± 42mo), with 48 events in the Intermediate RS.

Of 577 patients entered for TMA construction, 109 had insufficient tumour content for TMA core sampling. Each core was sampled with a diameter of 1000 μm and in triplicate per patient, leaving *n* = 468 patients and *n* = 1404 TMA total cores for spatial omics analyses. A more detailed consort diagram can be found in Supplementary Fig. 1.

### Datasets

Spatial transcriptomics dataset: due to too few cells or no cores, artefactual staining, sectioning, or imaging and limitations of the GeoMx DSP instrument, a further 58 patients were dropped, leaving *n* = 410 patients with whole-transcriptome spatial transcriptomics data. One core was selected per patient and segmented in the cytokeratin channel for epithelia vs microenvironment.

Spatial proteomics dataset: due to no tumour or too few cells, artefacts present within cores, or duplicated clinical data, 26 patients were dropped, leaving *n* = 440 patients for 5-marker multiplexed immunofluorescence-based spatial proteomics analysis. Three cores were used per patient for proteomics analyses.

Matched spatial proteomic-transcriptomic dataset: there were *n* = 359 patients with matched multiplexed immunofluorescence-based spatial proteomic and spatial transcriptomics data. Proteomic data was subset by TMA core ID’s with matched transcriptomic data. Variables relating to count, percentage and density were calculated within the matched subset when investigating potential transcriptomic pathways effected by proteomic variables.

Whole-slide image dataset: there were *n* = 437 whole-resection specimens with CD8 IHC data available. Orthogonal validation was performed using cytotoxic T-cell density data derived from whole resection specimens with an Intermediate RS (*n* = 151).

### Spatial proteomics

Multiplexed immunofluorescence (mIF) staining was carried out using a sequential OPAL™ tyramide signal amplification (TSA) multiplexing method using a 6-plex detection kit (#NEL871001KT) on a Bond-RXm Automated Research Stainer (Leica Biosystems, Newcastle, UK) on tissues within one month of their sectioning to minimise epitope degradation. Primary antibodies were applied sequentially with heat-induced epitope retrieval times, staining conditions and pH values optimised per antibody and OPAL™ fluorophore combination. The staining order was applied (Supplementary Table 1), and counterstained with spectral DAPI. Briefly, slides were blocked for 10 min with 150 μL of Akoya blocking buffer (#ARD1001EA, Akoya Biosciences, Menlo Park, CA) before incubating with 150 μL antibody at room temperature (RT) for 30 min. Slides were washed before applying 150 μL rabbit or mouse linker (DAKO, EnVision FLEX+ Rabbit: #SM805, EnVision FLEX+ Mouse: #SM804) for 20 min at RT, where used. One hundred and fifty microlitres of TSA-DIG was applied for 10 min, when used. Linkers/TSA-DIG steps were followed by a wash and 150 μL of DAKO horseradish peroxidase for 20 min, wash, and 150 μL of the requisite OPAL™ fluorophore for 30 min, or 20 min for OPAL™ 780. Slides were then thoroughly washed and subjected to epitope retrieval for the subsequent antibody, for 20 min at 95 °C, using either Leica Bond ER solution 1 (pH6) or solution 2 (pH9). Antibody suppliers and catalogue numbers were as follows: Syntec Scientific-CD4 (#104R-25), CD8 (#108M-95), CD68 (#168M-96), CD20 (#120M-86); Bio-Techne – Cytokeratin (#NBP2-29429); Cell Signalling Technology – FOXP3 (#D6O8R).

IHC staining (Supplementary Table 1) was carried out using the DAKO EnVision FLEX kit (#K802321-1, Agilent Technologies, Stockport, UK) with a DAKO PT-Link and Link-48 system. Antigen retrieval was performed at 97 °C for 20 min at pH 9 in EnVision FLEX high pH. Staining was performed in 48-slide batches with 5 min of FLEX peroxidase block, 20 min with CD8 (#IR62361-2, Agilent Technologies, Stockport, UK), and 5 min of FLEX DAB+ sub-chromo for 5 min. Slides were washed with buffer for two cycles before FLEX haematoxylin was applied for 3 min, washed, and slides dehydrated at 60 °C for 1 h. One millilitre drop of Sigma DPX was used for mounting and cover slipped. Tonsil tissue was used within each staining batch as a positive control to ensure intra-batch consistency.

### Spatial transcriptomics

A Nanostring GeoMx Morphology Marker Kit (GeoMx Solid Tumour Morphology Kit #121300310) staining for CD45, PanCK and SYTO13 was applied to FFPE-derived TMA tissues as outlined by the manufacturer, using a Leica Bond RX-m (Leica Biosystems, Newcastle UK). Antigen retrieval was performed in a Leica Bond RxM at 100 °C for 20 min in Tris-EDTA, with staining performed as per the manufacturer’s instructions (Nanostring GeoMx DSP Automated Slide Preparation User Manual). RNA targets were exposed by digestion in 0.1 μg/ml proteinase K (Nanostring RNA Auto Sample Prep Kit #121300316) for 15 min. RNA in situ hybridisation was performed at room temperature for 16 h. The whole transcriptome atlas (WTA) was applied, with tumour vs. microenvironment regions semantically segmented and RNA probes aspirated on the Nanostring Digital Spatial Profiler instrument^52^. One 660 μm region was taken per patient for segmentation, using PanCK to semantically segment two areas of interest: the TME from epithelia. Serial UV illumination of each compartment was used to sequentially collect mRNA probe barcodes from each segmented region. Collected probes were stored at −80 °C immediately after collection to preserve probes for sequencing. Library Preparation was performed at the Genomics Core Facility, Queen’s University of Belfast, Northern Ireland, according to MAN-10153-03 (Version Feb-2023). Resulting Sample Pools underwent AMPure Cleanup with KAPA Pure Beads (07983298001) followed by Quality Control checks with Qubit 1X dsDNA High Sensitivity Assay (Q33231) and Agilent Tapestation D1000 Assay (5067-5582). Two Sequencing Pools were made based on the total ROI area of the Sample Pools. These underwent further QC checks with Qubit 1X dsDNA High Sensitivity Assay (Q33231) and Fragment Analyser HS Fragment Analyser Assay (DNF-474) to determine Molarity. A Sequencing Depth Factor of 100 was chosen (for Whole Transcriptome Atlas/WTA). Each Sequencing Pool was run on the Illumina NovaSeq 6000 Sequencer (S4 200 Cycle v1.5 (20028313) and S2 100 Cycle v1.5 (20028316) carts utilised for the two different Sequencing Pools). The following Read Structure was utilised (read 1–27, index read 1–8, index read 2–8 and read 2–27). Sequenced reads were obtained in FASTQ format and processed through the GeoMx NGS pipeline to generate raw gene counts. Raw counts were imported into R Studio for quality control via Nanostring’s GeoMxWorkflow pipeline (R packages: *GeoMxWorkflow* v1.16.0, *GeomxTools* v3.14.0, *NanoStringNCTools* v1.18.0). Limit of quantitation (LOQ) was defined as the geometric mean of the negative control probes multiplied by the geometric standard deviation. Targets consistently below the LOQ were excluded. For normalisation, upper quartile (Q3) normalisation was applied: briefly, the count in one segment was divided by the 3rd quartile value for that segment, and subsequently multiplied by the geometric mean of the 3rd quartile of all segments.

### Hierarchical clustering

TME and EPI states were derived using patient-level cell densities from 5-marker multiplex immunofluorescence-based spatial proteomics data (*n* = 440). Densities were log-transformed (log1p) and z-scored by marker across patients, and clustering performed on the standardised density matrix using Euclidean distance and Ward’s minimum variance hierarchical clustering (ward.D2, R package *stats* v4.5.2). The number of *k* clusters was evaluated across *k* = 2–10 using resampling-based stability (*B* = 500, 80% of patients per subsample) quantified by the adjusted Rand index (R package *mclust* v6.1.2). For TME and EPI states, stability analysis indicated comparable stability for *k* ≥ 3 clusters. We therefore chose *k* = 4 for TME states and *k* = 3 for EPI states, which provided coherent immune states consistent with immune biology. Heatmaps were plotted using *ComplexHeatmap* (Bioconductor v2.26.0).

### Ligand-receptor analyses

To explore possible signalling programmes across stromal (TME) and epithelial compartments, we used OmniPath^25^ ligand-receptor annotations implemented in R Studio (R package *OmniPathR*, v3.18.2*)*. Unique ligand and receptor genes were extracted from the interaction table. Gene lists were then restricted to those present in our GeoMx expression matrix. For each compartment, expression was analysed as gene-wise z-scores. Differential expression analysis of OmniPath ligand and receptors between immune states was tested using Limma (Bioconductor v3.66.0) with empirical Bayes moderation (eBayes). Contrasts were defined between TME and EPI states. Multiple testing was controlled using the Benjamini–Hochberg FDR. For visualisation, we plotted volcano plots (R package *ggplot2*, v4.0.1) of log fold-changes (Δ*z*; Limma logFC) for the top-ranked genes per contrast, prioritising FDR-significant genes.

### Gene-set enrichment analysis

Hallmark gene sets were retrieved via *msigdbr* (v25.1.1, category 'H') and supplied to *fgsea* (Bioconductor, v1.36.0) as a pathway-gene mapping. Differential expression within stroma (TME) and epithelial (EPI) compartments was modelling using Limma (Bioconductor v3.66.0). Genes were restricted to numeric expression features in the GeoMx matrix and filtered to retain features with non-negligible variance within each compartment. For each contrast, genes were ranked by the Limma-moderated t-statistic and enrichment of Hallmark pathways was assessed using *fgsea* with minSize = 10, maxSize = 500. Pathways were considered significant at FDR ≤ 0.05. Directionality was defined by the sign of the normalised enrichment score (NES > 0 = enriched/up in the state. NES < 0 depleted/down in the state).

### Gene modules and regression

To relate immune densities or spatial proximity to functional or dysfunctional immune activity, we modelled gene expression modules against stromal immune densities, spatial proximities and genomic risk. Predefined gene modules were derived by aggregating the mean expression of curated gene sets, for: antigen presentation (HLA-DRA, HLA-DRB1, CIITA, CD74 and B2M), cytotoxicity (GZMA, GZMB, PRF1 and IFNG), exhaustion (PDCD1, TOX, CXCL13, HAVCR2, TIGIT and LAG3), checkpoint inhibition (PD-L1, PD-L2, CTLA4, LAG3, TIGIT and VSIR), M1-like macrophage (CXCL9, NOS2, TNF, CD80, CD86 and IL1B), M2-like macrophage (SPP1, CD163, TGFB1 and IL10) and extracellular matrix (COL1A1, COL3A1, TIMP1, TIMP2, MMP2 and MMP9). Module scores were z-standardised within the stromal compartment. Linear regression models (R package *stats*, v4.5.2) were fitted separately for each module, to include immune phenotype count and density, pairwise spatial proximity and Oncotype Dx RS as predictors. Densities were log-transformed (log1p) and z-scored. Distances were log-transformed (log1p) and z-scored and negated so that larger values represented greater proximity. For each model, the regression coefficient with 95% CI were estimated (R package *broom*, v1.0.10) and p-values corrected for multiple testing using the Benjamini–Hochberg (BH) method (flagged at discovery FDR < 0.1). In order to quantify unique variance explained by each predictor, semi-partial *R*^2^ values were calculated using bootstrap resampling (R package *boot*, v1.3-32. *B* = 5000 resamples). The Δ-*R*^2^ (variance loss) across resamples quantified the unique contribution of each predictor.

To investigate how macrophage density or cytotoxic T-cell density in the epithelia or stroma was related to immune gene activity, we modelled individual gene z-scores from each curated gene module above, as a function of density (cells/mm^2^). Densities were log-transformed (log1p), and z-score normalised within the stroma (macrophage, cytotoxic T-cell) or epithelia (cytotoxic T-cell). Ordinary least squares linear models (R package *stats*, v4.5.2) were fitted, with cluster robust standard errors (R package *sandwich*, v3.1-1*)*, stratified by TME states (TME-1 through TME-4) or EPI states (EPI-1 through EPI-3). To ensure transcriptomic data were not a reflection of greater cell infiltrates, models were adjusted for all cell densities within the target spatial compartment except the predictor variable (e.g. investigating gene changes with stromal macrophage density across TME states, all stromal densities were adjusted for except macrophage density). Group-specific slopes estimated (R package *emmeans*, v2.0.0*)* to give effect sizes per +1 SD change in the predictor variable. Slopes were back-transformed to enable interpretation, where changes in gene expression z-score could be viewed as intuitive steps in the predictor (e.g. for density: +50 cells/mm^2^). Multiple testing was controlled using the Benjamini–Hochberg method (flagged at discovery FDR < 0.10).

### Stromal-epithelial coupling of immune infiltrates

To quantify how intraepithelial immune infiltration scales with stromal immune infiltration, across both measured phenotypes and TME states, we aggregated cell detections at the TMA core level and modelled iTIL density as a function of sTIL density. For each core, we classified a leukocyte as intraepithelial or stromal by its signed distance from a tumour (PanCK^+^) epithelial mask: negative distances show cells within the mask. Densities were summarised per phenotype and TME state. We fit negative binomial mixed-effect models (R package *glmmTMB*, v1.1.14). Cores were nested within subjects, and interaction terms used for both TME state and phenotype. TME-4 (immune-cold) was excluded due to few count data inflating uncertainty. Global effects were tested using type-II Wald *χ*^2^ tests (R package *car*, v3.1-3). Phenotype- and TME state-specific estimates were extracted using estimated trends (R package *emmeans*, v2.0.0).

### Image capture

Fluorescent staining was captured using the Akoya PhenoImager™ HT Automated Quantitative Pathology Imaging System (Akoya Biosciences, Menlo Park, CA) at ×20 magnification. Fluorescent channels were optimised using a spectral library of breast tissues stained single-plex for each of the fluorophores and subjected to the outlined protocol (antigen retrieval times and pH conditions equal to their order in the staining stack). The spectral library was constructed to minimise both spectral bleed-through and tissue autofluorescence, and to maximise the captured signal:noise ratio of each respective marker during imaging. The final resolution of captured image scans was 0.4992 μm/px. Chromogenic IHC on WSIs were captured using a Leica Aperio AT2 (Leica Biosystems, Newcastle, UK) at ×20 magnification, with a final resolution of 0.5025 μm/px.

### Digital pathology and phenotype scoring

All digital image analysis (DIA) was undertaken in QuPath (v0.5.1)^53^. A publicly available script was utilised to stitch individual TMA core TIFF files to a singular pyramidal.ome.tif image (code available publicly via GitHub: https://gist.github.com/coltegelston/7c5ef58b32dd3a1b6a3f34926f01d6b2). Nuclear detection in the DAPI channel was performed using the StarDist (v0.5.0)^54^ QuPath plugin, or watershed cell detection in the haematoxylin channel (Supplementary Tables 3 and 4). Cell segmentation quality control was performed with a pathologist (C.A.G). Object detection classifiers were trained for individual markers using a Random Forest^55^ classifier within QuPath with default features. A Random Forest pixel-based classifier was trained to distinguish macrophage due to their irregular shape, which made object detection-based methods perform sub-optimally, and used to generate detections with a filtering step by a minimum size of 15 μm^2^ and a maximum 150 μm^2^. These thresholds were chosen as they performed best in separating individual macrophage instances while reducing overcalling from spectral bleed-through, seen with the PanCK (Opal 690) channel. For the separation of the TME compartments into epithelia versus microenvironment, a random forest pixel classifier was also trained with default features in the cytokeratin channel (mIF) or haematoxylin (IHC) and used to generate annotation masks of epithelia and TME. Subsequently, combination classifiers were constructed from single-marker object classifiers to discern all cells, including double-positive scoring such as CD4^+^FOXP3^+^ T-regs or interacting cells, and resolved hierarchically so that detections were binned into either epithelia or microenvironment annotation mask. This method afforded further granularity in analysing whether TILs were intratumoural (within epithelial compartment) or stromal (within microenvironment). Detected and phenotyped objects were exported and compared core-core, per patient, demonstrating strong intrapatient correlation (Supplementary Fig. 2). Artefactual cores (e.g. shearing, staining, blur and core translocation) and those with <100 cells were omitted from analysis, whereas artefact was manually annotated out of the analysis area when these occurred in whole-slide images. In order to generate a singular score per patient, the mean percentage of each cell phenotype was taken across available cores and used as a singular TIL score. Overall TIL scores were calculated using all detected objects within both tumour epithelia and microenvironment masks. Microenvironmental (sTIL) and epithelial (iTIL) scores were calculated similarly, using the detected cells within each segment respectively. Density was calculated using count/mm^2^ of the annotation mask. All immune scores across mIF and IHC can be found in Supplementary Table 2. Spatial analyses were performed using the 'Detection centroid distances 2D' script commands within QuPath. This command computes the 2D Euclidean distance (μm) between the centroid of a cell of a source class to the nearest centroid of a target class. These data were exported and analysed further in R Studio (v2025.09.1), and summarised as the median distance per patient. Examples of QuPath classifier generation for multiplex immunofluorescence-based spatial proteomics and orthogonal validation can be seen in Supplementary Figs. 3 and 4. All Ki67 values were calculated as outlined previously^56^.

### Statistical analyses

Normality of data distribution was assessed using the Kolmogorov–Smirnov test with Lilliefors correction (R package *nortest*, v1.0-4). Differences in values between two categories was assessed using pairwise Mann–Whitney (R package *stats*, v4.5.2). Multiple sample overall difference was assessed using the Kruskal–Wallis test (R package *stats*, v4.5.2), and Dunn’s test with a correction for multiple testing by the Benjamini–Hochberg method (flagged at FDR < 0.05) (R package *FSA*, v0.10.0). Drop-out and missingness analysis were assessed at the patient level within the Intermediate RS, comparing baseline covariates between included/excluded cases for each omics dataset. For categorical variables, Pearson’s *χ*^2^ test was used for frequencies >5, otherwise Fisher’s exact test was used (two-sided) (R package *stats*, v4.5.2). For continuous variables, the Wilcoxon rank-sum (two-sided) was used (R package *stats*, v4.5.2). Within each modality, comparisons were adjusted for multiple comparisons using the Benjamini–Hochberg method (FDR < 0.05).

### Survival analyses

Survival analyses were performed via the Kaplan–Meier method (R package *survival*, v3.8-3), and Likelihood ratio-*χ*^2^ tests and Harrell’s C-index were obtained from nested uni- and multivariable Cox models. All statistical tests were two-sided, with *α* < 0.05 considered statistically significant. Asterisk values of significance are shown as follows: ns *p* > 0.05, *p* < 0.05 (*), 0.01 ≥ *p* > 0.001 (**) and 0.001 ≥ *p* (***). For all models the proportional hazard assumption was verified by cox.zph tests (R package *survival*, v3.8-3. Supplementary Tables 5–8). To assess chemotherapy inferiority/superiority, we fit multivariable Cox models containing an interaction between adjuvant treatment (HT vs. HT + CT) and the continuous immune variable (counts/densities), adjusting for clinical covariates (menopausal status, age, tumour size, histological grade, luminal subtype). Non-linearity was explored with restricted cubic splines (3 knots at Harrell’s defaults of 10th, 50th and 90th percentile) using *rms*, with 95% CIs from model standard errors^57^ (R package *rms*, v8.1-0).

ASDs were computed by standardisation (R package *survival*, v3.8-3). From the fitted Cox model with a continuous treatment × biomarker interaction, we generated model-based survival curves with covariates fixed at typical values (numerics at the cohort median; factors at the modal level) and baseline stromal CD8 density set to prespecified levels (percentiles, median). ASD was calculated at 5, 10 and 15 years, where positive values favour endocrine therapy above chemoendocrine. Uncertainty was quantified with patient-level bootstrap resampling (1000 resamples) with percentile 95% CIs. Stromal CD8 density was modelled on the z-scale for estimation and translated to cells/mm² for interpretation and display.

Restricted mean survival time (RMST) was used to summarise treatment effects over the follow-up period (R package *survival*, v3.8-3). For a timepoint of 15 years, RMST equals the area under the survival curve up to 15-years. Delta-RMST was defined as:

1 $${\Delta RMST}\left(15{yrs}\right)={{RMST}}_{{HT}}-{{RMST}}_{{HT}}+{CT}$$

RMSTs were obtained by integrating the model-based survival functions to 15 years (from *survfit*, R package *survival*, v3.8-3*)* with bootstrap 95% CIs (1000 resamples).

Decision-curve analysis (DCA) was performed with a benefit-based variant^58^. Clinical utility of treatment selection in the randomised arms of the Intermediate RS was evaluated with DCA at the 15-year timepoint (R package *dcurves*, v0.5.1). For each patient, we estimated counterfactual 15-year absolute risks under endocrine therapy alone and chemoendocrine therapy from Cox proportional hazard models. From these, we defined chemotherapy harm at 15-years as the absolute risk increase:

2 $${\Delta Risk}=P({event}|{HT}+{CT})-P({event}|{HT})$$

So, positive values indicated chemotherapy inferiority. Absolute risks were obtained from each Cox model via the baseline cumulative hazard and individual linear predictors. We considered harm thresholds *t* ranging from 0 to 30% (in 1% steps). For each threshold, we evaluated a chemotherapy-sparing policy: withhold chemotherapy if ${\mathrm{\varDelta Risk}}$ ≥ *t*. For a given *t*, net benefit was computed per-patient and reported per 100 patients:

3 $${Net}\,{benefit}=\left({{Risk}}_{{HT}}-{{Risk}}_{{Policy}}\right)-\frac{t}{1-t}\times P\left({chemo}\,{under}\,{policy}({{t}})\right)$$

Where ${{\mathrm{Risk}}}_{{\mathrm{HT}}}$ is the mean 15-year risk if all patients received endocrine therapy only, and ${{\mathrm{Risk}}}_{{\mathrm{Policy}}}$ is the mean 15-year risk under the treatment allocation rule (e.g. menopausal status toward endocrine-only or chemoendocrine, etc.). Net benefit was plotted for the biomarker policy (treatment × sTIL CD8 density), menopausal status policy, and endocrine therapy for all, chemoendocrine for all. We obtained 95% bootstrap percentile intervals by resampling over 1000 replicates, recomputing net benefit over each replicate. Plots show median net benefit and 95% intervals.

### Reporting summary

Further information on research design is available in the Nature Portfolio Reporting Summary linked to this article.

## Supplementary information

Supplementary Information

Reporting Summary

Transparent Peer Review File

## Source data

Source Data

## Data Availability

Raw data for spatial transcriptomic data (GeoMx count matrices, imaging masks) can be found in ArrayExpress (E-MTAB-16811) at the following link (10.6019/e-mtab-16811), provided access has been granted (see below). Proteomics data (count, percentage, density, spatial metrics) and WSI orthogonal validation data (CD8: count, percentage, density) have been deposited in Zenodo (10.5281/zenodo.17288210). The data are available under restricted access due to patient privacy and ethics approval. Access can be obtained upon request by email to the senior author: darranoconnor@rcsi.ie. Requests should be submitted with a research proposal. Academic access will be made available to researchers whose full proposal for their use of the data has been approved by the TAILORx Tissue Bank and Cancer Trials Ireland. Commercial access will require further assessment by the Royal College of Surgeons Technology Transfer Office. We aim to approve requests within 6–8 weeks of their submission. All data are included in the Supplementary Information or available from the authors, as are unique reagents used in this article. The raw numbers for charts and graphs are available in the Source data file whenever possible. Source data are provided with this paper.
