# Supplementary material for: Spatial analyses implicate high stromal tumour-infiltrating CD8+ lymphocytes as a negative predictive marker for chemotherapy in estrogen receptor-positive breast cancer
Source: Nat Commun. 2026 Jun 23;17:4863. doi: 10.1038/s41467-026-73432-2 (PMC13291258; doi:10.1038/s41467-026-73432-2)
Supplement: Supplementary file 1 — Supplementary Information [file 41467_2026_73432_MOESM1_ESM.pdf]

Supplementary Information:

Spatial analyses implicate high stromal tumour-infiltrating $CD8^+$ lymphocytes as a negative predictive marker for chemotherapy in estrogen receptor-positive breast cancer

Authors and Affiliations

Zak Kinsella[1§†], Chowdhury Arif Jahangir[2§], Hannah Nyarkoah Nyarko[1], Daria Kalinska-Lysiak[1§], Claudia Aura Gonzalez[2], Verena Murphy[3], Tony O'Grady[4], Joanna Fay[4], Katherine Sheehan[4], Arman Rahman[2], John P. Crown[5], Catherine M. Kelly[6], Simon McDade[7], Ina Woods[8], Niamh M. Connolly[8], Jochen H.M. Prehn[8*], William M. Gallagher[2*], Darran P. O'Connor[1*].

[1]School of Pharmacy & Biomolecular Sciences, Royal College of Surgeons in Ireland, Dublin 2, Ireland; [2]UCD Conway Institute, University College Dublin, Belfield, Dublin 4, Ireland; [3]Cancer Trials Ireland, Dublin 2, Ireland; [4]Molecular Pathology Laboratory, Beaumont Hospital, Dublin 9, Ireland; [5]St. Vincent's University Hospital, Dublin 4, Ireland; [6]Mater Hospital, Dublin 1, Ireland; [7]School of Medicine, Dentistry and Biomedical Sciences, Patrick G Johnston Centre for Cancer Research, Queens University Belfast, Belfast, United Kingdom; [8]Department of Physiology & Medical Physics, Centre for Systems Medicine, Royal College of Surgeons in Ireland, Dublin 2, Ireland

[§]These authors contributed equally

[*]These authors jointly supervised this work

[†] Corresponding author: Dr Zak Kinsella: zakkinsella@rcsi.com.

- Supplementary Figures -

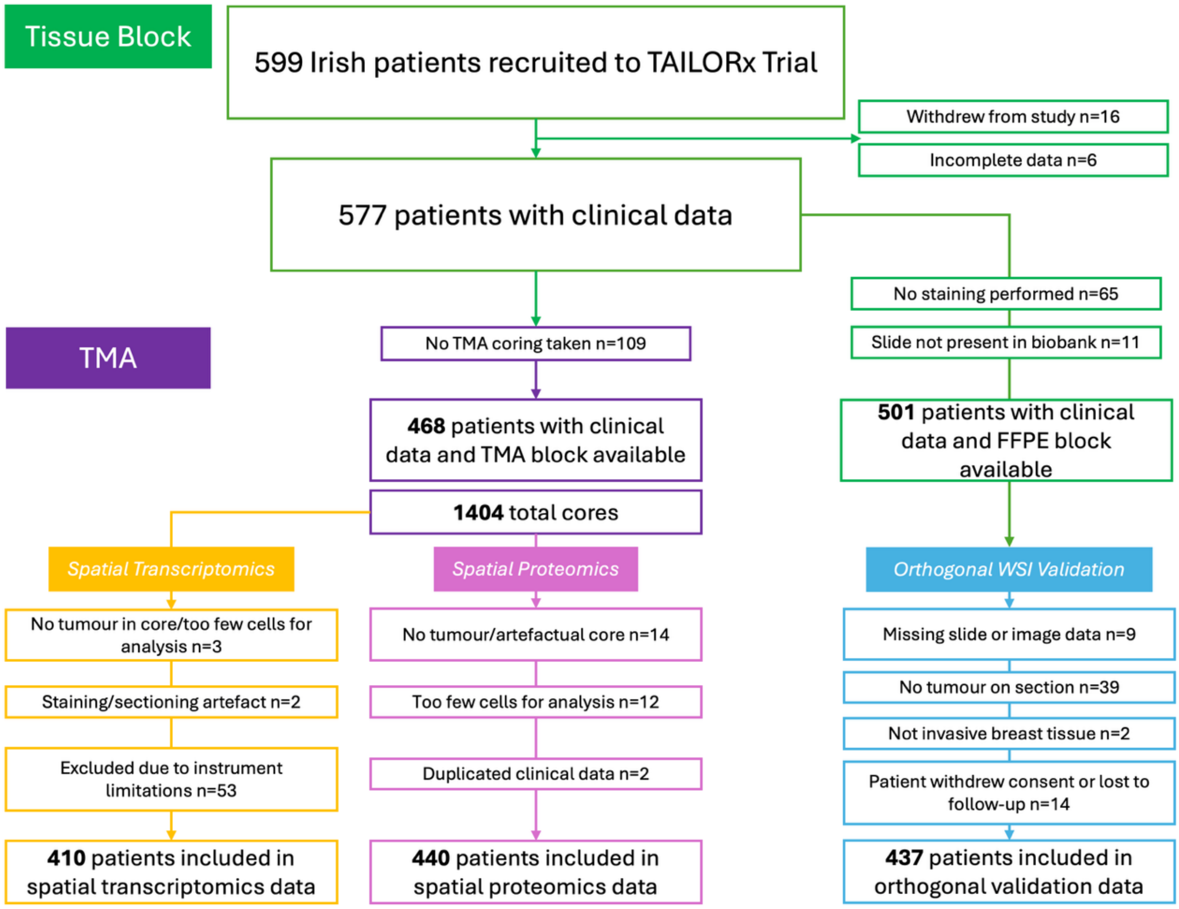

Supplementary Figure 1. *CONSORT diagram of Irish patients previously enrolled on the TAILORx clinical trial, split for spatial transcriptomics, spatial proteomics, and orthogonal WSI validation analyses.* ***N.B.*** *Spatial transcriptomics instrument limitations allude to the restricted aspiration area of the Nanostring GeoMx DSP, as this was too small to accommodate all 8 rows of 12 TMA cores.*

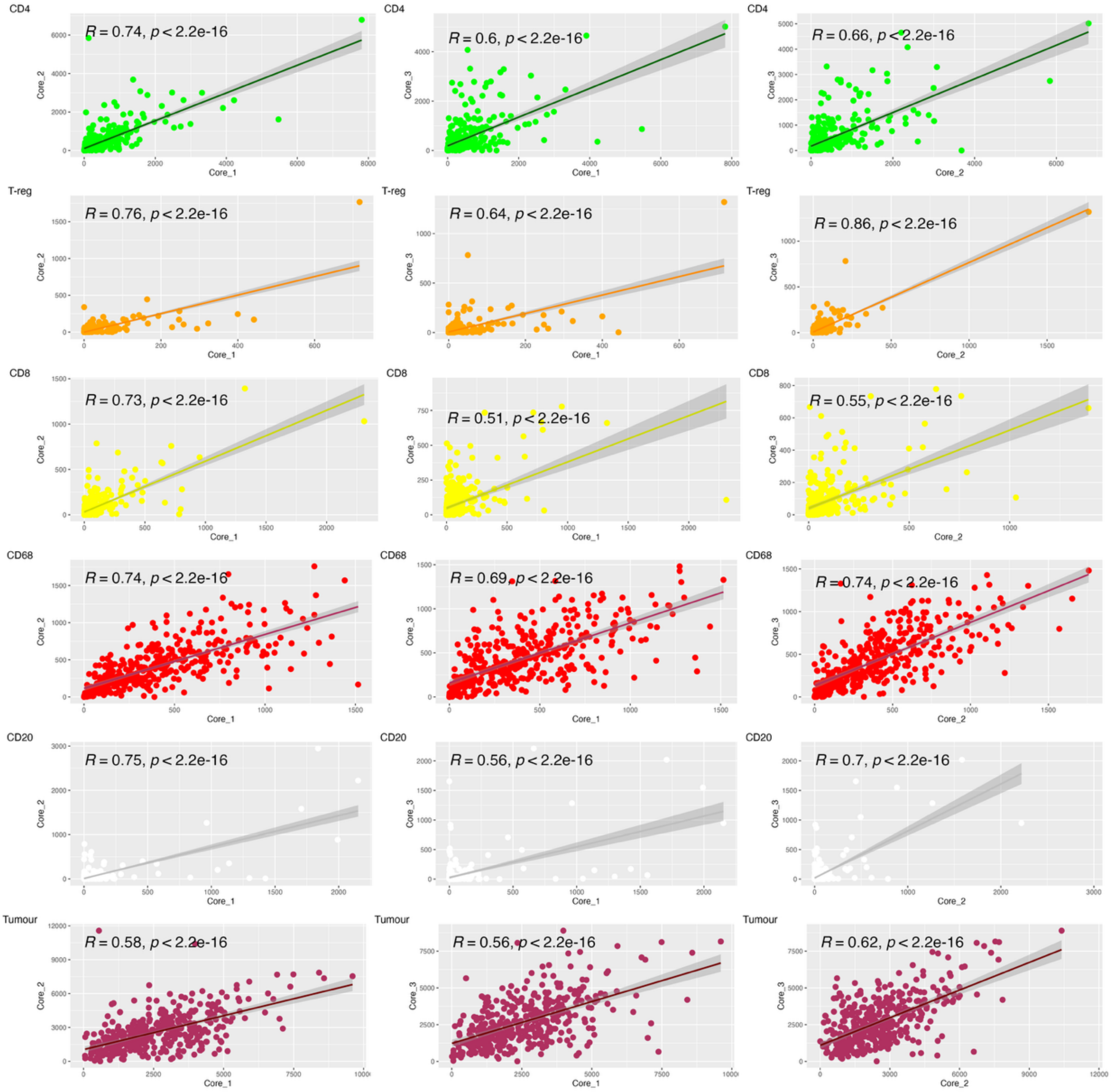

Supplementary Figure 2. *Core-core correlation for objects detected by spatial proteomics workflow. R = Pearsons correlation coefficient.*

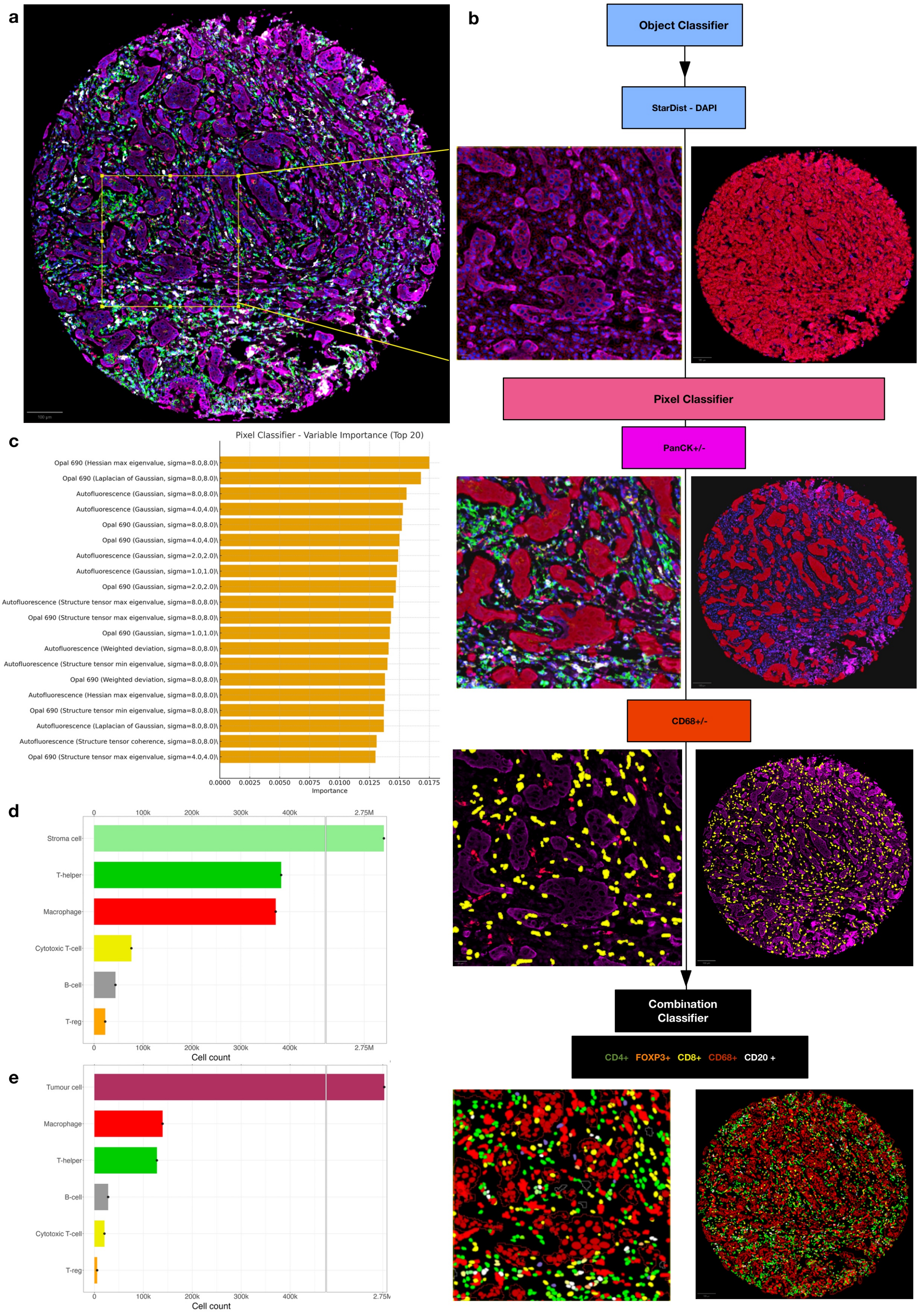

Supplementary Figure 3. *Digital pathology workflow for multiplexed immunofluorescence data.* ***A.*** *Illustrative example of a representative TMA core and region of interest (ROI: yellow).* ***B.*** *Classifier generation workflow, with example outputs at the core level (middle row) and ROI level (bottom row).* ***C.*** *Variable importance for the pixel classifier.* ***D.-E.*** *Barcharts of detected phenotype frequencies within all*

cores. Left plot shows a zoom in to the y-axis. For ***D.*** *Tumour Microenvironment (TME i.e. Stroma).* ***E.*** *Tumour epithelia (i.e. tumour).*

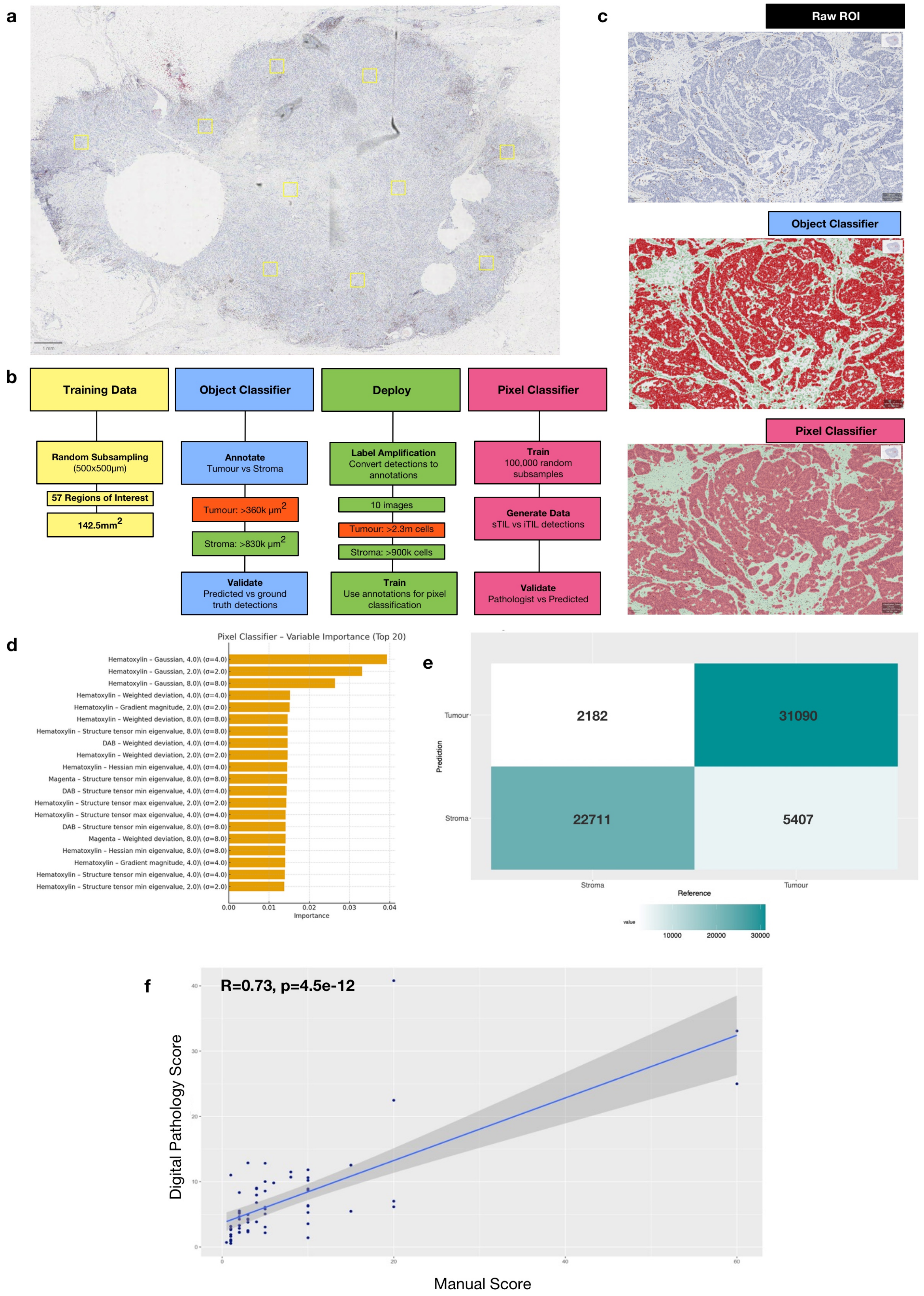

Supplementary Figure 4. *Digital pathology workflow for orthogonal CD8 Immunohistochemistry.* ***A.*** *Illustrative example of a representative whole slide image (WSI) and regions of interest (ROI: yellow).* ***B.*** *Classifier generation workflow. Briefly, an object detection classifier was trained to generate tumour vs stroma detections. These were then used as annotations for generation of a pixel classifier for the same*

classes. ***C.*** *Illustrative examples of object detection and pixel classification in an ROI****.*** ***D.*** *Variable importance for the pixel classifier.* ***E.*** *Confusion matrix for the generated object detection classifier.* ***F.*** *Pearson's correlation of stromal CD8 percentage versus pathologist ground truth in a hold-out validation set of n=68 WSIs.*

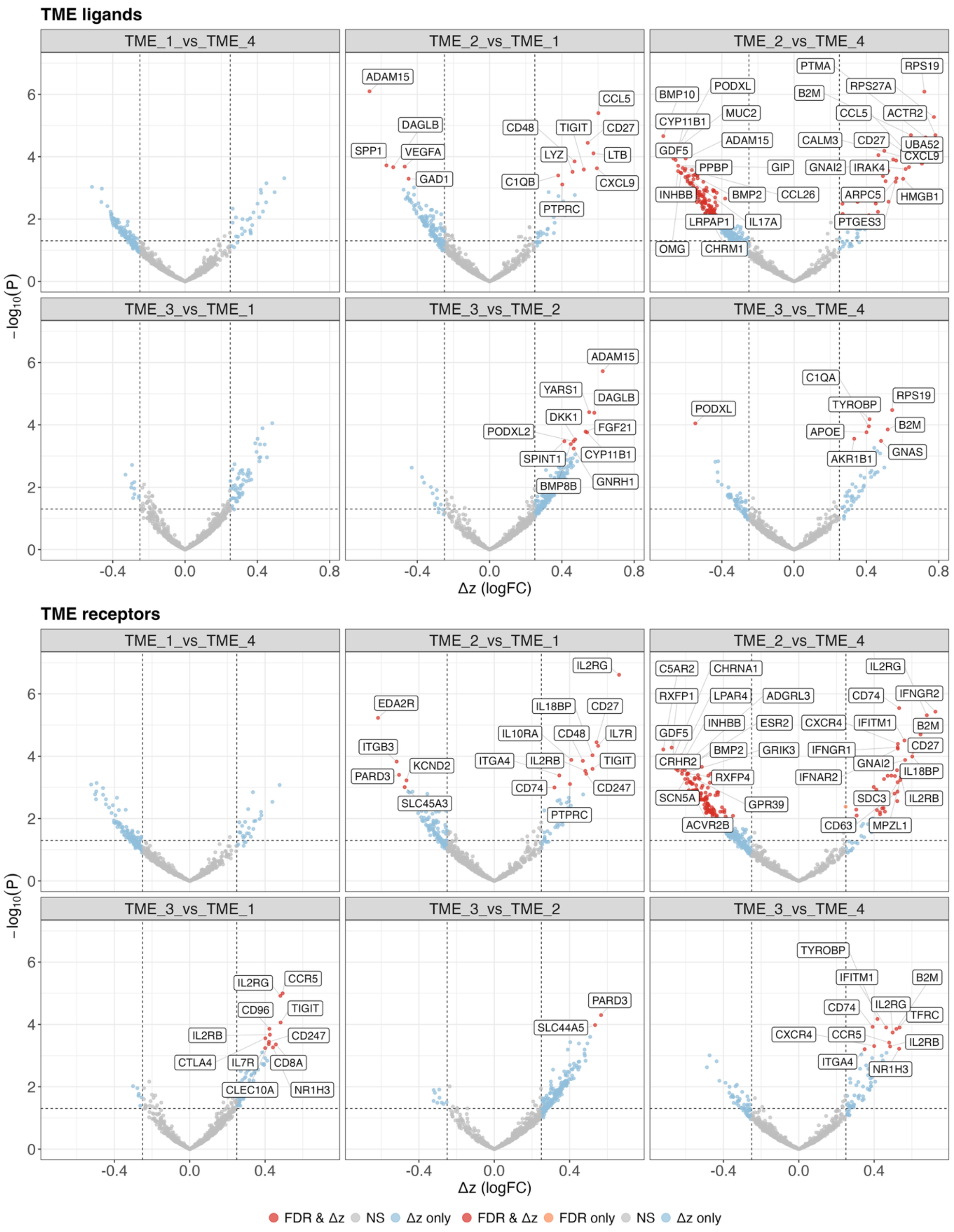

Supplementary Figure 5. *Volcano plots of state-wise differentially expressed Ligand-Receptor genes derived from the OmniPath database. Δ-z is the difference in mean z-scored expression between clusters. Dashed lines denote Δ-z thresholds and p-thresholds derived from two-sided empirical Bayes moderated t-tests from Limma, encoded by blue point colour. Those passing adjustment for multiple comparisons with the Benjamini-Hochberg method are in red (FDR<0.05)*

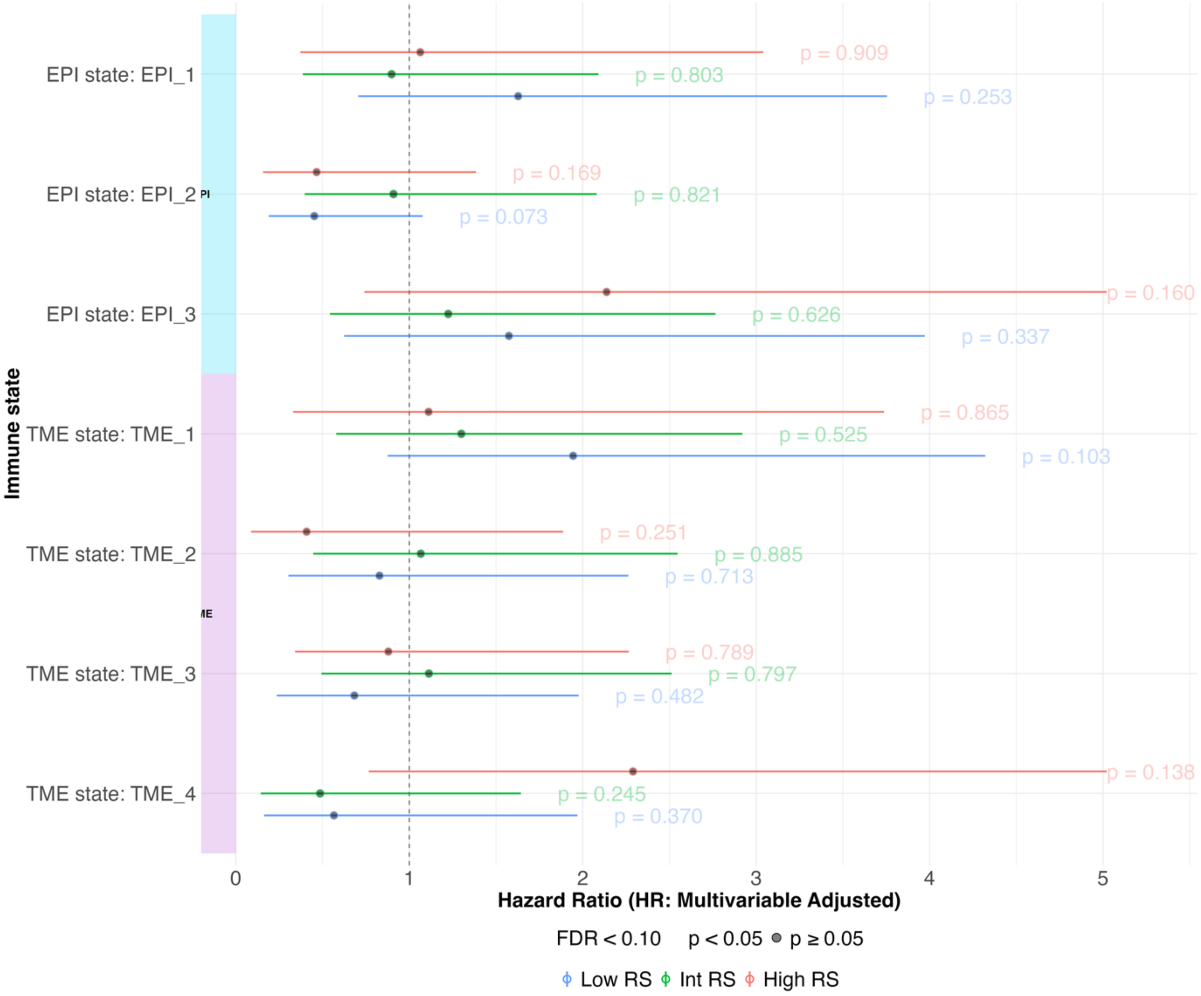

Supplementary Figure 6. *Multivariable Cox regression of TME and EPI states across the Oncotype Dx RS (Low RS n=167, Int RS n=183, High RS n=90). Forest plot points show hazard ratios (HR) and error bars encode 95% confidence intervals. Models are adjusted by the same set of clinical covariates (patient age, menopausal status, histological grade, primary tumour size, luminal subtype). P-values and 95%CI are taken from Wald tests. Point/line opacity encodes false discovery rate (FDR) after Benjamini-Hochberg correction.*

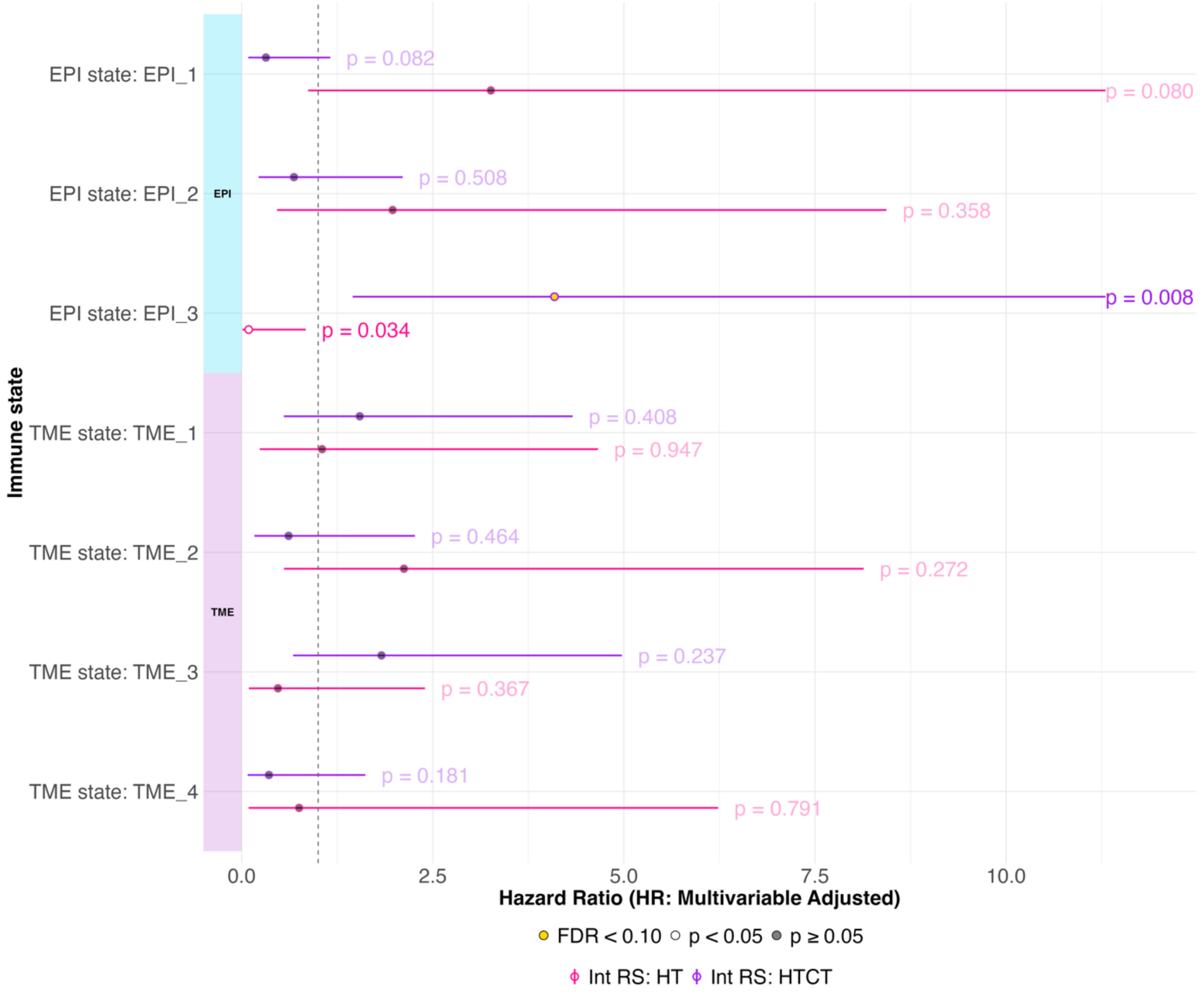

Supplementary Figure 7. *Multivariable Cox regression of TME and EPI states across randomised treatment arms of the Intermediate RS (HT n=123, HT+CT n=118). Forest plots points show hazard ratios (HR) and error bars encode 95% confidence intervals. Models are adjusted by the same set of clinical covariates (patient age, menopausal status, histological grade, primary tumour size, luminal subtype). P-values and 95%CI are taken from Wald tests. Point/line opacity encodes false discovery rate (FDR) after Benjamini-Hochberg correction.*

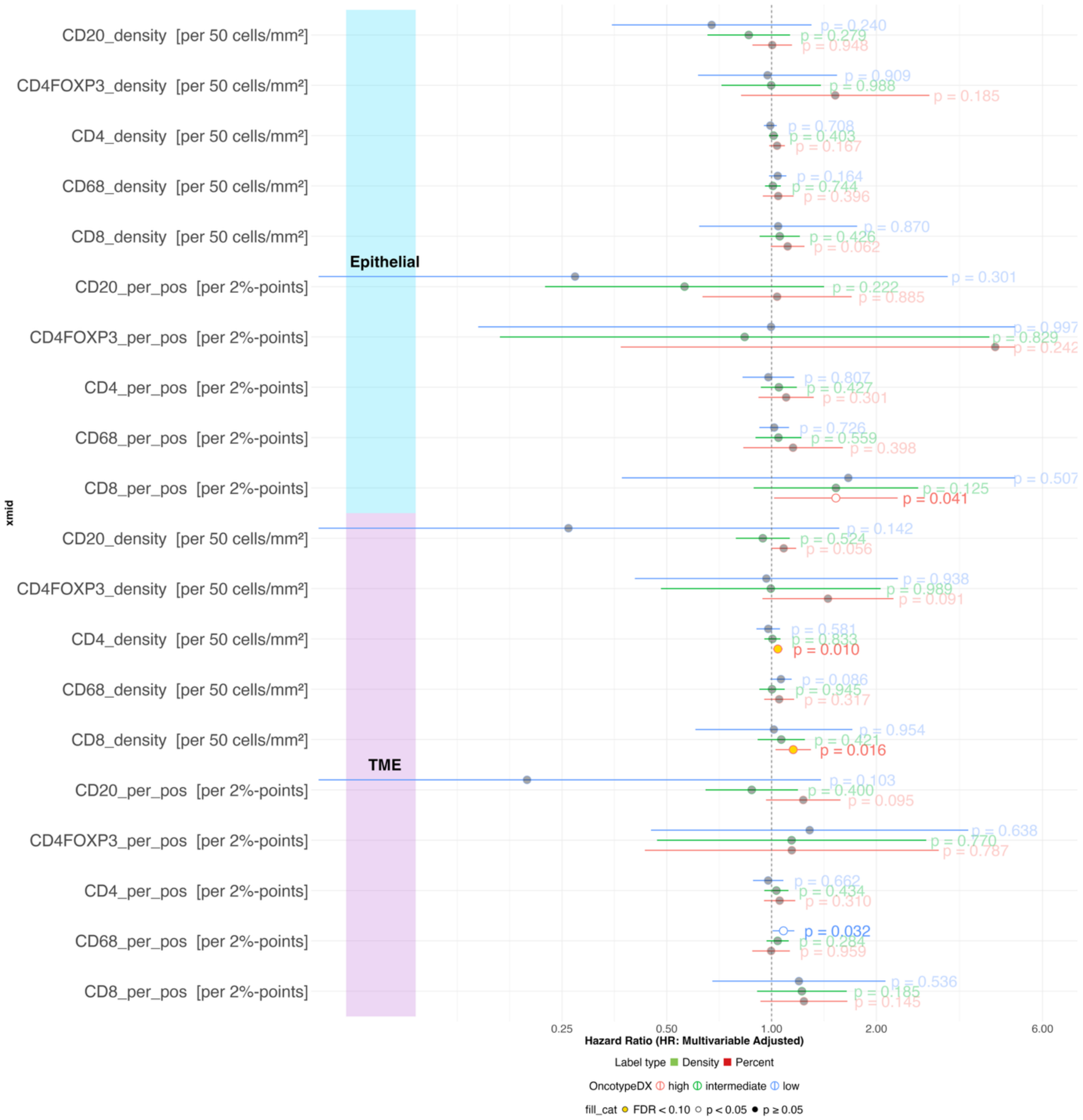

Supplementary Figure 8. *Multivariable Cox regression across the Oncotype Dx RS (Low RS n=167, Int RS n=183, High RS n=90) . Forest plots points show hazard ratios (HR) and error bars encode 95% confidence intervals for the predictor density (green), or percentage (red) shown. Models are adjusted by the same set of clinical covariates (patient age, menopausal status, histological grade, primary tumour size, luminal subtype). P-values and 95%CI are taken from Wald tests. Point/line opacity encodes false discovery rate (FDR) after Benjamini-Hochberg correction.*

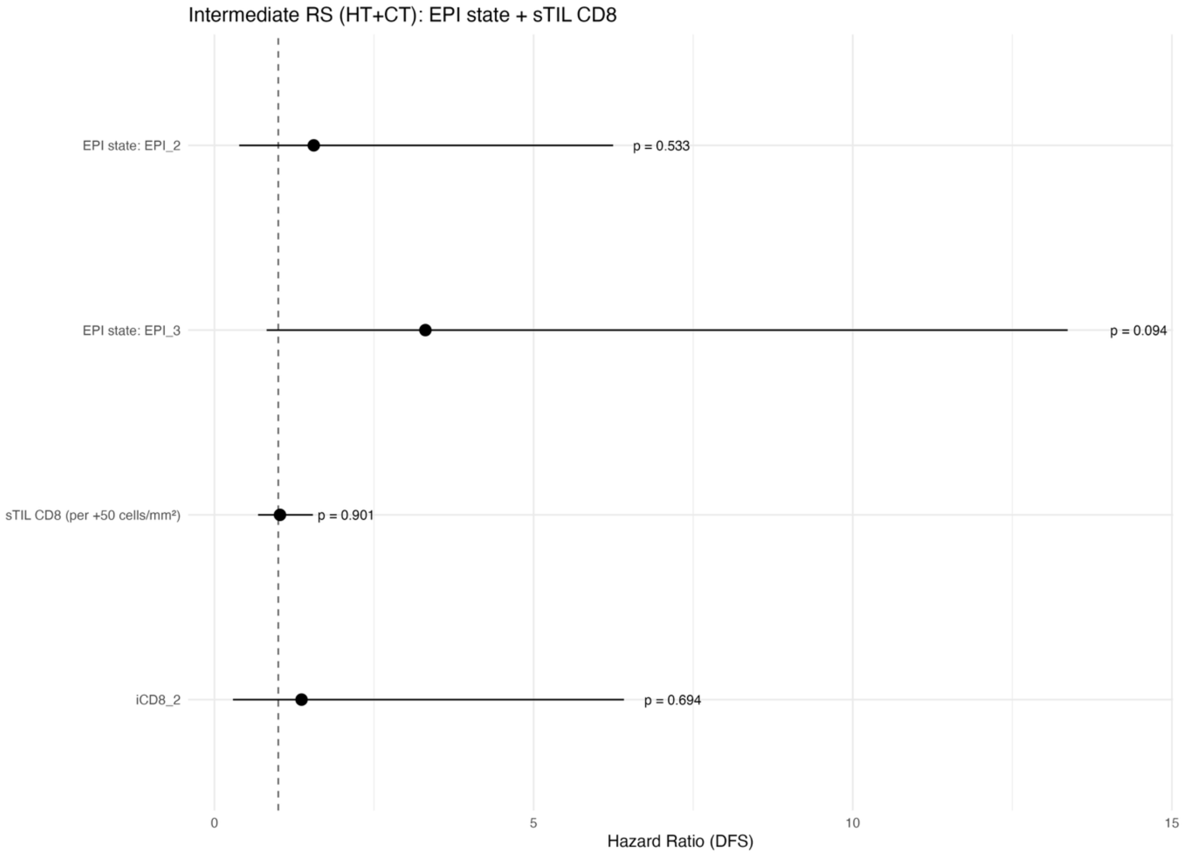

Model	Δ LR-χ^2	p-value
**EPI States + sTIL CD8 density**	1.42	0.233
**EPI States + iTIL CD8%**	1.56	0.211
**EPI States + sTIL CD8 density + iTIL CD8%**	1.58	0.454
**sTIL CD8 density + EPI States**	3.38	0.184
**iTIL CD8% + EPI States**	3.18	0.204
**sTIL CD8 density + iTIL CD8% + EPI States**	3.18	0.203

Supplementary Figure 9. *Multivariable Cox regression analysis of EPI states and stromal CD8 density in the Intermediate RS chemoendocrine arm (n=118). Forest plot points show hazard ratios (HR) and error bars encode 95% CI. Table shows the outcome of omnibus $\Delta LR\text{-}\chi^2$ tests between nested models (left variable in the model) and models with an additional term (right variable in the model).*

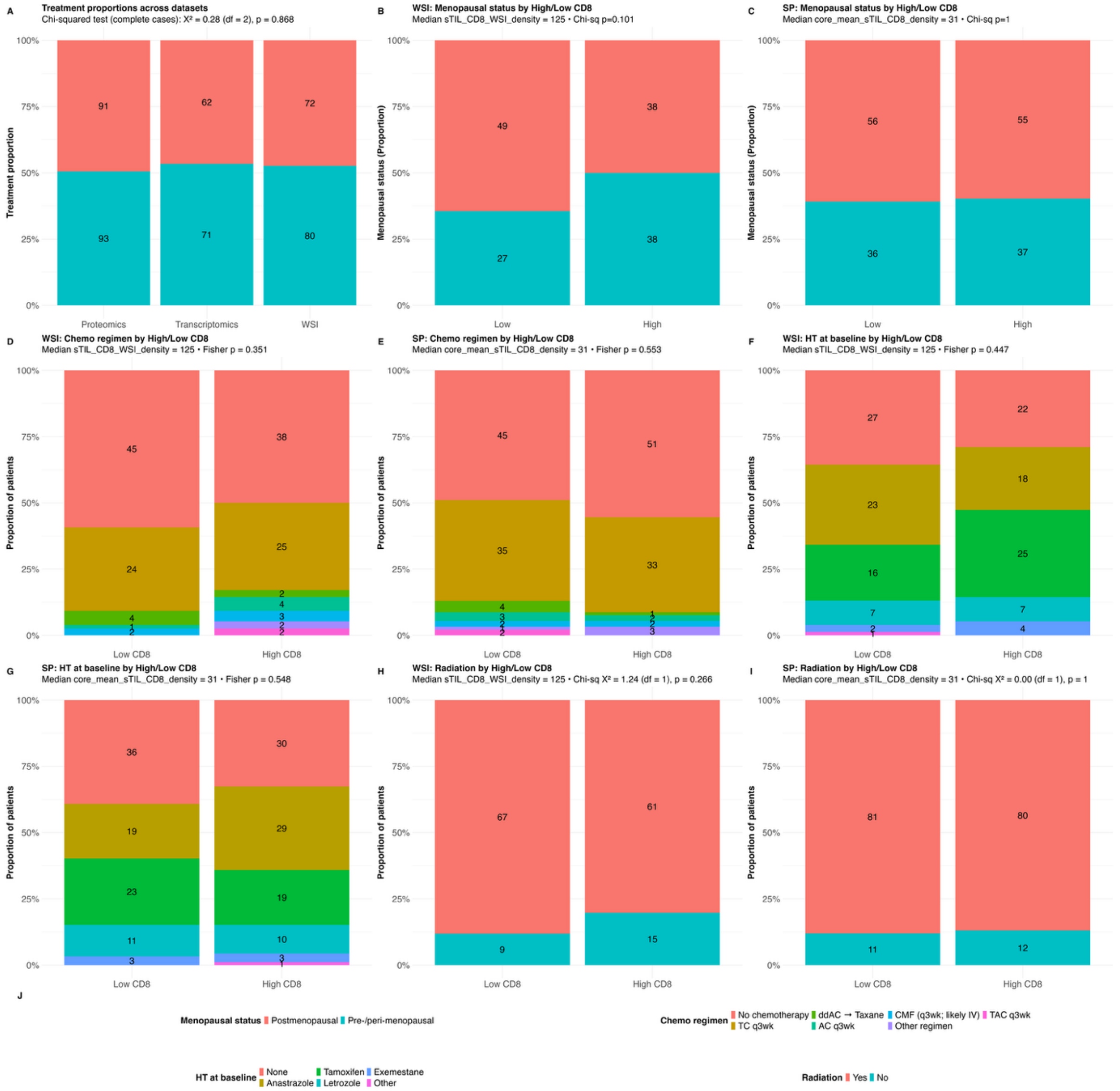

Supplementary Figure 10. *Drop-out and missing comparison using Chi-squared and Fishers-exact tests (categorical, two-sided) or Wilcoxon rank-sum (continuous, two-sided), across datasets for the Intermediate Oncotype Dx RS (RS16-25).* ***A.*** *Received adjuvant treatment across spatial omics datasets.* ***B.-C.*** *Groups of menopausal status across high vs low CD8 density, for* ***B.*** *WSIs,* ***C.*** *Spatial proteomics.* ***D.-E.*** *Groups of received adjuvant chemotherapy regimen across high vs low CD8 density, for* ***D.*** *WSIs,* ***E.*** *Spatial proteomics.* ***F.-G.*** *Groups of received adjuvant hormone therapy regimen across high vs low CD8 density, for* ***F.*** *WSIs,* ***G.*** *Spatial proteomics.* ***H.-I.*** *Groups of received radiotherapy across high vs low CD8 density, for* ***H.*** *WSIs.* ***I.*** *Spatial proteomics.*

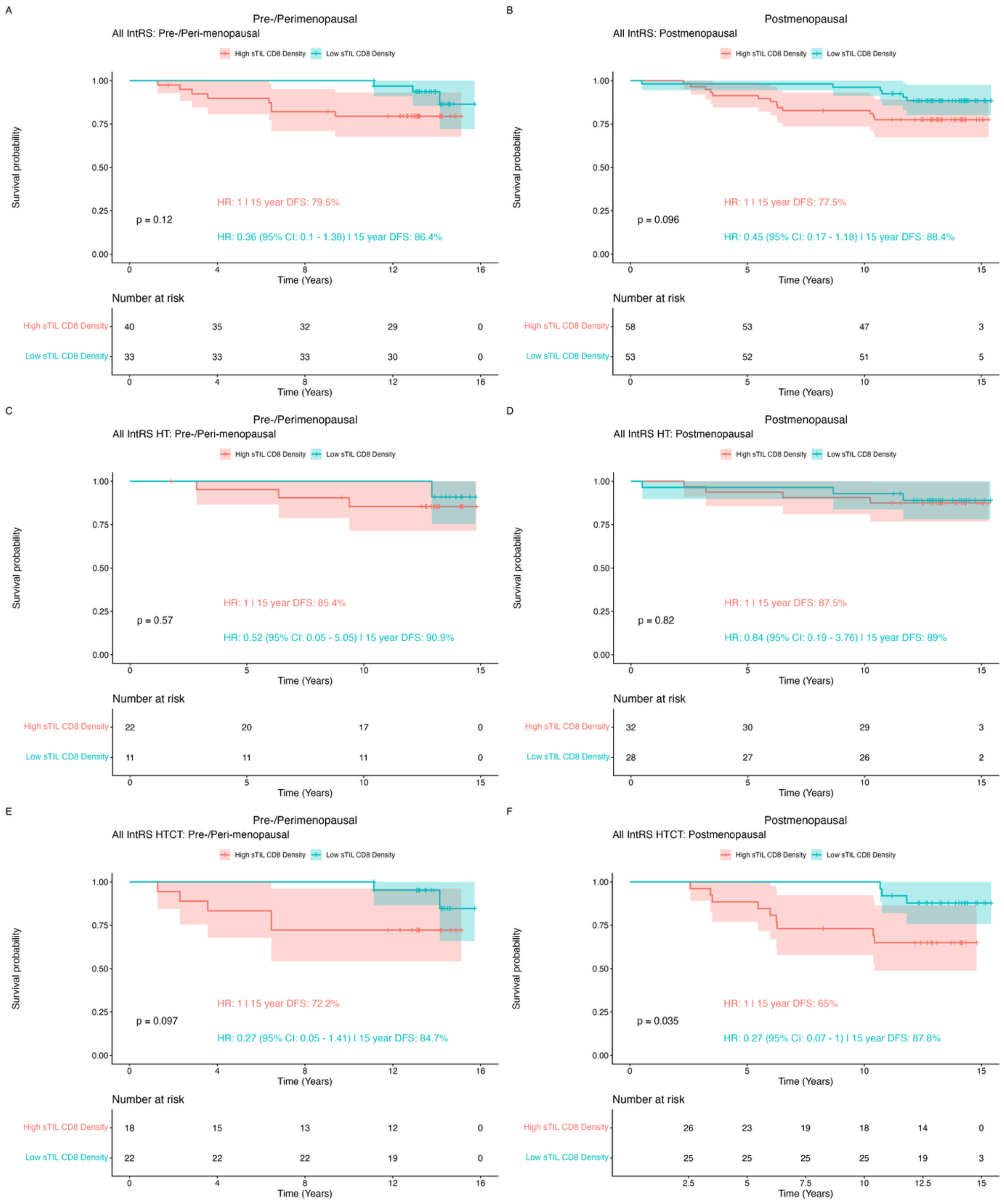

Supplementary Figure 11*. Kaplan-Meier curves of stromal CD8 density (median-dichotomised) across strata of menopausal status in the Intermediate RS.* ***A-B.*** *Intermediate RS overall,* ***C-D.*** *Intermediate RS endocrine arm,* ***E-F.*** *Intermediate RS chemoendocrine arm. HT = endocrine therapy, HTCT = chemoendocrine therapy.*

- Supplementary Tables -

Supplementary Table 1. *Antibodies and Dyes used in 6-plex mIF Spatial Proteomics, Spatial Transcriptomics, and Orthogonal Validation Assessment of Cohort Tissue Microarray and Whole-Resection Samples*

Antibody	Clonality, Species	Clone	Antigen Retrieval	Antibody Conc.	Fluorophore	Fluorophore Conc.	Linker Usage
**CD4**	Monoclonal Rabbit	EP204	pH9	1:140	Opal 520	1:200	Yes
**CD8**	Monoclonal Mouse	C8/144B	pH9	1:450	Opal 570	1:500	Yes
**FOXP3**	Monoclonal Rabbit	D608R	pH6	1:285	Opal 620	1:75	Yes
**CD68**	Monoclonal Mouse	PG-M1	pH6	1:100	Opal 650	1:150	No
**Pan-Cytokeratin**	Monoclonal Mouse	AE1/AE3	pH6	1:500	Opal 690	1:150	No
**CD20**	Monoclonal Mouse	L26	pH6	1:280	Opal 780	1:25	No*
**Pan-Cytokeratin**[1]	Monoclonal Mouse	AE1+AE3	pH9	-	AF 532	-	No
**CD45**[1]	Monoclonal Mouse	2B11+PD7/26	pH9	-	AF 594	-	No
**SYTO13**[1]	-	-	pH9	-	AF 647	-	No
**CD8**[!]	Monoclonal Mouse	C8/144B	pH9	Proprietary	-	-	-

** Opal TSA-DIG:*

1 Antibodies-dyes used for morphology marking, Nanostring GeoMx.

! Antibody used for orthogonal validation on whole-resection specimens.

Supplementary Table 2*. Marker score derived from spatial proteomics data (TMA and WSI), across patient samples overall, and within epithelial (iTIL) and TME (sTIL) segments.*

Median percentage (%)

**Marker**	Overall	Overall IQR (Q1-Q3)	Stroma	Stroma IQR (Q1-Q3)	Epithelia	Epithelia IQR (Q1-Q3)
**CD4⁺**	3.59	1.1 – 8.5	6.67	2.2 – 13.3	1.40	0.4 – 4.4
**CD4⁺FOXP3⁺**	0.05	0 – 0.3	0.10	0 – 0.5	0.009	0 – 0.08
**CD8⁺**	0.71	0.3 – 1.6	1.48	0.5 – 2.9	0.25	0 – 0.7
**CD68⁺**	6.93	3.2 – 10.5	12.70	5.9 – 19.5	3.36	1.2 – 6.3
**CD20⁺**	0.10	0 – 0.4	0.10	0 – 0.5	0.06	0 – 0.3
**CD8^{WSI}**	2.78	1.4-4.4	4.24	2.2-7.6	0.84	0.4-1.8
Median density (cells/mm²)						
**CD4⁺**	130.2	41.1 – 368.3	122.7	37.8 - 310	126.4	33.2 – 379.2
**CD4⁺FOXP3⁺**	1.9	0 – 11.3	1.6	0 – 11.6	0.8	0 – 7.2
**CD8⁺**	28.2	10.1 – 68.8	27.0	9.3 – 75.8	22.3	7.5 – 62.1
**CD68⁺**	301.4	130.4 – 469.4	272.6	115.6 – 439.9	295.5	108.6 – 556.7
**CD20⁺**	3.7	0.8 – 17.0	1.8	0 – 10.9	4.8	0 – 22.8
**CD8^{WSI}**	226.0	117-423.0	125.0	65-246.0	74.3	35.4-163.9

Supplementary Table 3*. Cell detection parameters used for StarDist plugin for detection of DAPI+ cells in multiplexed immunofluorescence images in QuPath.*

Cell detection parameters - mIF	
**Parameter**	**Value**

**Model**	dsb2018_heavy_augment.pb
**Channels**	DAPI
**Normalise Percentiles**	10, 99
**Threshold**	0.45
**Pixel Size**	0.499
**Cell Expansion**	5.0μm
**Measure Shape**	TRUE
**Measure Intensity**	TRUE
**Include Probability**	TRUE
**Contrain to Parent**	FALSE

Supplementary Table 4. *Cell detection parameters used for orthogonal validation of CD8 immunohistochemistry on whole-slide images in QuPath.*

Cell detection parameters - WSI	
**Parameter**	**Value**
**Detection Image**	Optical density sum
**Requested pixel size**	0.15μm
**Background Radius**	4μm<br>(opening by reconstruction TRUE)
**Median Filter Radius**	1μm
**Sigma**	1μm
**Minimum Area**	10μm^2

**Maximum Area**	400μm^2
**Threshold**	0.07
**Max Background Density**	4<br>(split by shape TRUE; Exclude DAB FALSE)
**Cell Expansion**	1.3μm<br>(include cell nucleus FALSE; sooth boundaries TRUE; make measurements TRUE)

Supplementary Table 5. *Stromal immune variable (sTIL) Proportional Hazard (PH) checks across strata of Oncotype Dx RS categories. All variables shown are adjusted for the same set of clinical covariates (age, menopausal status, histological grade, tumour size, luminal subtype). PH assumptions were assessed using scaled Schoenfeld residual tests based on Cox PH fitted separately within each Oncotype Dx group, and adjusted for the indicated covariates. Covariate specific and global chisq tests are shown.*

Covariate	rho	Chi-square	df	*p*	Global Chi-square	Global *p*
			**High RS**			
CD20_density [per 50 cells/mm^2]	NA	NA	NA	NA	NA	NA
CD20_per_pos [per 2%-points]	−0.306	0.068	1	0.794	16.560	0.020
CD4FOXP3_density [per 50 cells/mm^2]	NA	NA	NA	NA	NA	NA
CD4FOXP3_per_pos [per 2%-points]	−0.119	0.000	1	0.992	16.689	0.020
CD4_density [per 50 cells/mm^2]	NA	NA	NA	NA	NA	NA
CD4_per_pos [per 2%-points]	−0.172	0.166	1	0.684	17.061	0.017
CD68_density [per 50 cells/mm^2]	NA	NA	NA	NA	NA	NA
CD68_per_pos [per 2%-points]	−0.462	0.001	1	0.972	18.903	0.008
CD8_density [per 50 cells/mm^2]	NA	NA	NA	NA	NA	NA
CD8_per_pos [per 2%-points]	0.100	0.347	1	0.556	14.506	0.043
			**Intermediate RS**			
CD20_density [per 50 cells/mm^2]	−0.229	0.319	1	0.572	11.692	0.111
CD20_per_pos [per 2%-points]	−0.343	1.020	1	0.313	12.412	0.088
CD4FOXP3_density [per 50 cells/mm^2]	0.017	0.109	1	0.741	11.636	0.113
CD4FOXP3_per_pos [per 2%-points]	0.053	0.147	1	0.702	11.572	0.116
CD4_density [per 50 cells/mm^2]	−0.057	0.022	1	0.881	11.941	0.103
CD4_per_pos [per 2%-points]	0.070	0.274	1	0.600	11.802	0.107
CD68_density [per 50 cells/mm^2]	−0.350	2.458	1	0.117	14.069	0.050
CD68_per_pos [per 2%-points]	−0.162	0.869	1	0.351	11.656	0.112
CD8_density [per 50 cells/mm^2]	−0.132	0.921	1	0.337	13.224	0.067
CD8_per_pos [per 2%-points]	−0.121	0.567	1	0.451	12.588	0.083
			**Low RS**			
CD20_density [per 50 cells/mm^2]	−0.307	1.257	1	0.262	8.042	0.329
CD20_per_pos [per 2%-points]	−0.341	1.183	1	0.277	8.076	0.326

Covariate	rho	Chi-square	df	p	Global Chi-square	Global p
CD4FOXP3_density [per 50 cells/mm^2]	−0.139	0.160	1	0.690	6.132	0.524
CD4FOXP3_per_pos [per 2%-points]	−0.123	0.210	1	0.647	5.875	0.554
CD4_density [per 50 cells/mm^2]	0.184	1.813	1	0.178	7.043	0.424
CD4_per_pos [per 2%-points]	0.240	2.608	1	0.106	7.860	0.345
CD68_density [per 50 cells/mm^2]	−0.251	0.558	1	0.455	7.030	0.426
CD68_per_pos [per 2%-points]	−0.142	0.230	1	0.631	6.256	0.510
CD8_density [per 50 cells/mm^2]	−0.207	1.461	1	0.227	7.306	0.398
CD8_per_pos [per 2%-points]	−0.181	1.353	1	0.245	6.524	0.480

Notes. Scaled Schoenfeld residuals (cox.zph); p values are for PH tests. Models where the PH test was not computable are shown with NA values.

***Supplementary Table 6**. Epithelial immune variable (iTIL) Proportional Hazard (PH) checks across strata of Oncotype Dx RS categories. All variables shown are adjusted for the same set of clinical covariates (age, menopausal status, histological grade, tumour size, luminal subtype). PH assumptions were assessed using scaled Schoenfeld residual tests based on Cox PH fitted separately within each Oncotype Dx group, and adjusted for the indicated covariates. Covariate specific and global chisq tests are shown.*

Covariate	rho	Chi-square	df	p	Global Chi-square	Global p
**High RS**						
CD20_density [per 50 cells/mm^2]	NA	NA	NA	NA	NA	NA
CD20_per_pos [per 2%-points]	−0.223	0.011	1	0.917	16.585	0.020
CD4FOXP3_density [per 50 cells/mm^2]	−0.035	0.018	1	0.895	15.852	0.027
CD4FOXP3_per_pos [per 2%-points]	−0.039	0.021	1	0.886	15.890	0.026
CD4_density [per 50 cells/mm^2]	NA	NA	NA	NA	NA	NA
CD4_per_pos [per 2%-points]	−0.152	0.000	1	0.984	17.135	0.017
CD68_density [per 50 cells/mm^2]	NA	NA	NA	NA	NA	NA
CD68_per_pos [per 2%-points]	−0.442	0.020	1	0.887	19.648	0.006
CD8_density [per 50 cells/mm^2]	NA	NA	NA	NA	NA	NA
CD8_per_pos [per 2%-points]	0.274	1.124	1	0.289	13.658	0.058

Covariate	rho	Chi-square	df	*p*	Global Chi-square	Global *p*
**Intermediate RS**						
CD20_density [per 50 cells/mm^2]	−0.229	0.957	1	0.328	11.917	0.103
CD20_per_pos [per 2%-points]	−0.244	0.996	1	0.318	12.066	0.098
CD4FOXP3_density [per 50 cells/mm^2]	0.027	0.015	1	0.902	11.678	0.112
CD4FOXP3_per_pos [per 2%-points]	0.029	0.006	1	0.936	11.681	0.112
CD4_density [per 50 cells/mm^2]	−0.107	0.008	1	0.927	11.909	0.104
CD4_per_pos [per 2%-points]	−0.047	0.015	1	0.902	11.796	0.107
CD68_density [per 50 cells/mm^2]	−0.086	0.143	1	0.706	11.621	0.114
CD68_per_pos [per 2%-points]	−0.020	0.006	1	0.939	11.623	0.114
CD8_density [per 50 cells/mm^2]	−0.106	0.329	1	0.566	12.533	0.084
CD8_per_pos [per 2%-points]	−0.027	0.068	1	0.794	12.254	0.093
**Low RS**						
CD20_density [per 50 cells/mm^2]	−0.295	0.775	1	0.379	7.231	0.405
CD20_per_pos [per 2%-points]	−0.333	2.376	1	0.123	9.661	0.209
CD4FOXP3_density [per 50 cells/mm^2]	−0.075	0.000	1	0.989	5.831	0.560
CD4FOXP3_per_pos [per 2%-points]	−0.176	0.276	1	0.599	6.287	0.507
CD4_density [per 50 cells/mm^2]	0.225	1.639	1	0.200	6.947	0.434
CD4_per_pos [per 2%-points]	0.133	0.637	1	0.425	6.149	0.522
CD68_density [per 50 cells/mm^2]	−0.226	0.980	1	0.322	6.843	0.445
CD68_per_pos [per 2%-points]	−0.161	0.262	1	0.608	5.774	0.566
CD8_density [per 50 cells/mm^2]	0.035	0.024	1	0.878	5.647	0.582
CD8_per_pos [per 2%-points]	0.042	0.000	1	0.992	5.507	0.598

Notes. Scaled Schoenfeld residuals (cox.zph); *p* values are for PH tests. Models where the PH test was not computable are shown with NA values.

Supplementary Table 7*. Stromal immune variable (sTIL) Proportional Hazard (PH) checks across randomised treatment arms of the Intermediate Oncotype Dx RS. All variables shown are adjusted for the same set of clinical covariates (age, menopausal status, histological grade, tumour size, luminal subtype). PH assumptions were assessed using scaled Schoenfeld residual tests based on Cox PH fitted separately within each Oncotype Dx group, and adjusted for the indicated covariates. Covariate specific and global chisq tests are shown.*

Endocrine

Covariate	rho	Chi-square	df	*p*	Global Chi-square	Global *p*
		**HT**				
CD20_density [per 50 cells/mm^2]	−0.476	3.208	1	0.073	9.121	0.244
CD20_per_pos [per 2%-points]	−0.446	1.428	1	0.232	6.963	0.433
CD4FOXP3_density [per 50 cells/mm^2]	0.023	0.004	1	0.949	6.114	0.527
CD4FOXP3_per_pos [per 2%-points]	0.032	0.042	1	0.838	6.011	0.538
CD4_density [per 50 cells/mm^2]	0.068	0.042	1	0.838	2.951	0.890
CD4_per_pos [per 2%-points]	0.363	0.844	1	0.358	7.305	0.398
CD68_density [per 50 cells/mm^2]	−0.300	1.149	1	0.284	3.895	0.792
CD68_per_pos [per 2%-points]	−0.261	0.608	1	0.436	6.312	0.504
CD8_density [per 50 cells/mm^2]	−0.084	0.328	1	0.567	6.958	0.433
CD8_per_pos [per 2%-points]	−0.169	0.315	1	0.575	6.687	0.462
**Chemoendocrine**						
CD20_density [per 50 cells/mm^2]	−0.072	0.032	1	0.858	9.585	0.213
CD20_per_pos [per 2%-points]	−0.077	0.022	1	0.881	9.621	0.211
CD4FOXP3_density [per 50 cells/mm^2]	0.094	0.305	1	0.581	11.207	0.130
CD4FOXP3_per_pos [per 2%-points]	0.090	0.006	1	0.939	10.771	0.149
CD4_density [per 50 cells/mm^2]	0.056	0.255	1	0.614	12.062	0.099
CD4_per_pos [per 2%-points]	−0.011	0.024	1	0.877	10.870	0.144
CD68_density [per 50 cells/mm^2]	−0.295	2.239	1	0.135	10.988	0.139
CD68_per_pos [per 2%-points]	−0.178	0.565	1	0.452	10.109	0.182
CD8_density [per 50 cells/mm^2]	0.061	0.567	1	0.452	12.162	0.095
CD8_per_pos [per 2%-points]	0.013	0.177	1	0.674	11.874	0.105

Notes. Scaled Schoenfeld residuals (cox.zph); *p* values are for PH tests. Models where the PH test was not computable are shown with NA values.

Supplementary Table 8. *Epithelial immune variable (iTIL) Proportional Hazard (PH) checks across randomised treatment arms of the Intermediate Oncotype Dx RS. All variables shown are adjusted for the same set of clinical covariates (age, menopausal status, histological grade, tumour size, luminal subtype). PH assumptions were assessed using scaled Schoenfeld residual tests based on Cox PH fitted separately within each Oncotype Dx group, and adjusted for the indicated covariates. Covariate specific and global chisq tests are shown.*

Endocrine

Covariate	rho	Chi-square	df	*p*	Global Chi-square	Global *p*
		**HT**				
CD20_density [per 50 cells/mm^2]	−0.230	0.513	1	0.474	6.112	0.527
CD20_per_pos [per 2%-points]	−0.289	0.807	1	0.369	6.137	0.524
CD4FOXP3_density [per 50 cells/mm^2]	0.183	0.528	1	0.468	5.923	0.549
CD4FOXP3_per_pos [per 2%-points]	0.190	0.487	1	0.485	5.966	0.544
CD4_density [per 50 cells/mm^2]	NA	NA	NA	NA	NA	NA
CD4_per_pos [per 2%-points]	0.317	0.528	1	0.467	6.277	0.508
CD68_density [per 50 cells/mm^2]	−0.136	0.640	1	0.424	3.101	0.876
CD68_per_pos [per 2%-points]	−0.040	0.152	1	0.697	5.806	0.563
CD8_density [per 50 cells/mm^2]	0.159	0.108	1	0.743	6.334	0.501
CD8_per_pos [per 2%-points]	0.153	0.026	1	0.873	6.605	0.471
**Chemoendocrine**						
CD20_density [per 50 cells/mm^2]	−0.079	0.297	1	0.586	9.179	0.240
CD20_per_pos [per 2%-points]	−0.073	0.221	1	0.639	9.209	0.238
CD4FOXP3_density [per 50 cells/mm^2]	0.094	0.178	1	0.673	10.626	0.156
CD4FOXP3_per_pos [per 2%-points]	0.106	0.132	1	0.717	10.632	0.156
CD4_density [per 50 cells/mm^2]	−0.067	0.092	1	0.761	13.696	0.057
CD4_per_pos [per 2%-points]	0.010	0.502	1	0.479	14.344	0.045
CD68_density [per 50 cells/mm^2]	0.075	0.094	1	0.759	10.379	0.168
CD68_per_pos [per 2%-points]	0.015	0.005	1	0.942	10.134	0.181
CD8_density [per 50 cells/mm^2]	0.138	1.880	1	0.170	13.735	0.056
CD8_per_pos [per 2%-points]	0.154	2.071	1	0.150	13.897	0.053

Notes. Scaled Schoenfeld residuals (cox.zph); *p* values are for PH tests. Models where the PH test was not computable are shown with NA values.
