# Supplementary material for: Spatial analyses implicate high stromal tumour-infiltrating CD8+ lymphocytes as a negative predictive marker for chemotherapy in estrogen receptor-positive breast cancer
Source: Nat Commun. 2026 Jun 23;17:4863. doi: 10.1038/s41467-026-73432-2 (PMC13291258; doi:10.1038/s41467-026-73432-2)
Supplement: Supplementary file 2 — Reporting Summary [file 41467_2026_73432_MOESM2_ESM.pdf]

nature portfolio

Corresponding author(s): Zak Kinsella, Darran O'Connor

Last updated by author(s): Apr 20, 2026

Reporting Summary

Nature Portfolio wishes to improve the reproducibility of the work that we publish. This form provides structure for consistency and transparency in reporting. For further information on Nature Portfolio policies, see our Editorial Policies and the Editorial Policy Checklist.

Statistics

For all statistical analyses, confirm that the following items are present in the figure legend, table legend, main text, or Methods section.

n/a	Confirmed	
☐	☒	The exact sample size (*n*) for each experimental group/condition, given as a discrete number and unit of measurement
☐	☒	A statement on whether measurements were taken from distinct samples or whether the same sample was measured repeatedly
☐	☒	The statistical test(s) used AND whether they are one- or two-sided<br>*Only common tests should be described solely by name; describe more complex techniques in the Methods section.*
☐	☒	A description of all covariates tested
☐	☒	A description of any assumptions or corrections, such as tests of normality and adjustment for multiple comparisons
☐	☒	A full description of the statistical parameters including central tendency (e.g. means) or other basic estimates (e.g. regression coefficient) AND variation (e.g. standard deviation) or associated estimates of uncertainty (e.g. confidence intervals)
☐	☒	For null hypothesis testing, the test statistic (e.g. *F*, *t*, *r*) with confidence intervals, effect sizes, degrees of freedom and *P* value noted<br>*Give P values as exact values whenever suitable.*
☒	☐	For Bayesian analysis, information on the choice of priors and Markov chain Monte Carlo settings
☒	☐	For hierarchical and complex designs, identification of the appropriate level for tests and full reporting of outcomes
☐	☒	Estimates of effect sizes (e.g. Cohen's *d*, Pearson's *r*), indicating how they were calculated

Our web collection on statistics for biologists contains articles on many of the points above.

Software and code

Policy information about availability of computer code

Data collection	Digital pathology data was collected using QuPath (version 0.5.1), using StarDist (v0.5.0).<br>Multiplexed Immunofluorescence imaging was acquired using an Akoya PhenoImager HT at 20x magnification, using InForm software (version 2.6). Spatial transcriptomics was perfomed on a Nanostring GeoMx Digital Spatial Profiler (software version 3.1.2)
Data analysis	All data was analysed in R Studio. Key packages used and versions were as follows:<br>R Studio: dcurves (v0.5.1), survival (v3.8-3), rms (v8.1-0), stats (v4.5.2), FSA (v0.10.0), nortest (v1.0-4), emmeans (v2.0.0), glmmTMB (v1.1.14), sandwich (v3.1-1), broom (v1.0.10), fgsea (Bioconductor v3.66.0), msigdbr (v25.1.1), ggplot2 (v4.0.1), OmniPathR (v3.18.2), Limma (Bioconductor v3.66.0), ComplexHeatmap (Bioconductor v2.26.0), mclust (v6.1.2), GeoMxWorkflow (v1.16.0), GeomxTools (v3.14.0), NanoStringNCTools (v1.18.0),

For manuscripts utilizing custom algorithms or software that are central to the research but not yet described in published literature, software must be made available to editors and reviewers. We strongly encourage code deposition in a community repository (e.g. GitHub). See the Nature Portfolio guidelines for submitting code & software for further information.

Data

Policy information about availability of data

All manuscripts must include a data availability statement. This statement should provide the following information, where applicable:

- Accession codes, unique identifiers, or web links for publicly available datasets
- A description of any restrictions on data availability
- For clinical datasets or third party data, please ensure that the statement adheres to our policy

De-identified clinical variables and processed tissues that support the findings of this study are available under restricted access from Cancer Trials Ireland, under the TAILORx Tissue Bank (CTRIAL-IE 12-30. NCT02050750), per data use agreements and institutional acceptance. Source data for the figures (ROI/Subject level CSVs) and analysis outputs are available upon request to the corresponding senior author (DOP) at DOI: 10.5281/zenodo.17288210. Raw WSI, TMA images, and DSP image files contain potentially identifying information and are not publicly shareable. Controlled access can be requested from the data custodian subject to ethics approval and an accepted research proposal.

Research involving human participants, their data, or biological material

Policy information about studies with human participants or human data. See also policy information about sex, gender (identity/presentation), and sexual orientation and race, ethnicity and racism.

Reporting on sex and gender	All patients in this cohort were female.
Reporting on race, ethnicity, or other socially relevant groupings	Race/ethnicity variables were not consistently reported in the tissue bank dataset and thus were not used in analyses. No inferences were made based on socially defined groups.
Population characteristics	Inclusion criteria was specified by the original TAILORx trial (NCT00310180): this was predominantly hormone-receptor positive (estrogen receptor and/or progesterone receptor positive disease), HER2-negative, axillary lymph node-negative as assessed by sentinel lymph node biopsy, tumour size 1.1-5.0cm, with Oncotype Dx RS used for categorisation of risk. Further details on exhaustive inclusion criteria for the NCT00310180 trial can be found on the corresponding trial.gov page.<br>Inclusion in the TAILORx Tissue Bank (NCT02050750) required previous registration with ICORG 06-31 TAILORx trial, participation in trial arms, and having adequate tumour tissue available.<br>Summaries of patient characteristics can be found in Table 1 of the main manuscript.
Recruitment	Samples were obtained from the TAILORx Tissue Bank (CTRIAL-IE 12-30) comprising patients previously enrolled/registered to the TAILORx trial. This study performed no prospective intervention. It required previous registration with the TAILORx trial and adequate tumour tissue available.
Ethics oversight	Ethical approval for the study was provided by the institutional ethics committees of 11 Cancer Trials Ireland-affiliated hospitals, along with the Ethics Committee of Beaumont Hospital, the Royal College of Surgeons in Ireland (Tallagh Hospital; St James's Hospital; Letterkenny General Hospital: REC 2014 56/2014/03; REC 2016-07 List 25 (4)., Beaumont Hospital: REC 13/81., Cork University Hospital; Bon Secours Hospital: ECM 3 (ttt) 07/01/14; ECM 4 (ddd) 03/12/13 & ECM 3 (yyyyy) 21/06/16., Galway University Hospital 17/12., Mater Misericordiae University Hospital; Mater Private Hospital: 1/378/1590., Sligo Regional Hospital; St Vincent's Univeristy Hospital; University Hospital Limerick; Waterford Regional Hospital: n/a). Written informed consent provided by patients enrolled in the prior TAILORx trial included consent for the use of data and biological samples for future studies, provided the study was ethically approved. Under the CTRIAL-IE 12-30 study protocol, formalin-fixed paraffin-embedded (FFPE) tissue blocks of the identified eligible patients were retrieved from pathology archives and shipped to the study biobank.

Note that full information on the approval of the study protocol must also be provided in the manuscript.

Field-specific reporting

Please select the one below that is the best fit for your research. If you are not sure, read the appropriate sections before making your selection.

☒ Life sciences ☐ Behavioural & social sciences ☐ Ecological, evolutionary & environmental sciences

For a reference copy of the document with all sections, see nature.com/documents/nr-reporting-summary-flat.pdf

Life sciences study design

All studies must disclose on these points even when the disclosure is negative.

Sample size	We used all available cases meeting QC from the tissue bank: n=577 subjects overall, modality-specific subsets GeoMX DSP n=410, mIF n=442, CD8-IHC n=437. Precision and internal validity were assessed with 95% CIs and bootstrap resampling.
Data exclusions	Pre-specified QC excluded failed staining, ROIs with insufficient tissue or poor segmentation, extreme artefacts (shearing, folding, bubbling, etc.). Exact counts per modality are detailed above and in Supplementary Materials Fig.S1 CONSORT/flow diagrams.
Replication	Technical replication was achieved by sampling triplicate 1mm TMA cores per patient. TMA scoring was aggregated across multiple cores at the subject and ROI level (e.g. patient sTIL score is the mean sTIL score of cores available after QC).<br>Robustness of results was evaluated with bootstrap resampling and alternate model policies (see methods). To mitigate sampling bias and for

	orthogonal validation of the translatable biomarker, we also performed whole-slide image IHC on the same cohort.
Randomization	Original clinical treatment assignment of the TAILORx trial specified prospective randomisation of patients with an Intermediate RS (RS 11-25) 1:1 to receive either endocrine therapy alone or combination chemoendocrine therapy.
Blinding	Image analysis and transcriptomics analyses were conducted using pre-specified pipelines and blinded to clinical outcomes. Statistical analyses were performed on de-identified datasets with treatment and outcome concealed during feature extraction (e.g. densities, distances, counts for immune phenotyping).

Reporting for specific materials, systems and methods

We require information from authors about some types of materials, experimental systems and methods used in many studies. Here, indicate whether each material, system or method listed is relevant to your study. If you are not sure if a list item applies to your research, read the appropriate section before selecting a response.

Materials & experimental systems

n/a	Involved in the study
☐	☒ Antibodies
☒	☐ Eukaryotic cell lines
☒	☐ Palaeontology and archaeology
☒	☐ Animals and other organisms
☐	☒ Clinical data
☒	☐ Dual use research of concern
☒	☐ Plants

Methods

n/a	Involved in the study
☒	☐ ChIP-seq
☒	☐ Flow cytometry
☒	☐ MRI-based neuroimaging

Antibodies

Antibodies used	Multiplexed Immunofluorescence: CD4 (Clone: EP204. RRID:AB_1516770. Syntec Scientific #104R-25: https://www.cellmarque.com/antibodies/CM/2186/CD4_EP204). CD8 (Clone C8/144B. RRID:AB_1158206. Syntec Scientific #108M-95: https://www.cellmarque.com/antibodies/CM/35/CD8_C8-144B). FOXP3 (Clone: D608R, RRID:AB_2797979. Cell Signalling Technology #D6O8R: https://www.cellsignal.com/products/primary-antibodies/foxp3-d6o8r-rabbit-monoclonal-antibody-bsa-and-azide-free/72338?srsltid=AfmBOopMvwzA03m3Rf5TtMc7lDkIQREmcqSvKJZaoXqqLwbEwrNiZ6EQ). CD68 (Clone: PG-M1. RRID:AB_1158188. Syntec Scientific #168M-96: https://www.cellmarque.com/antibodies/CM/52/CD68_Kp-1). Pan-cytokeratin (Clone: AE1/AE3. RRID:AB_3319254. BioTechne #NBP2-29429: https://www.bio-techne.com/p/antibodies/cytokeratin-pan-antibody-ae-1-ae-3_nbp2-29429). CD20 (Clone: L26. RRID:AB_1158148. Syntec Scientific #120M-86: https://www.cellmarque.com/antibodies/CM/38/CD20_L26).<br>Spatial Transcriptomics ROI Segmentation/GeoMX DSP: CD45 (Clone 2B11+PD7/26. RRID: AB_3287678). Pan-cytokeratin (Clone AE1+AE3. RRID: AB_2924722) - Nanostring Solid Tumour Morphology Kit (#121300310).<br>Whole-slide immunohistochemistry: CD8 (Clone C8/144B. RRID: AB_2892113. Agilent Technologies #IR62361-2: https://www.agilent.com/en/product/immunohistochemistry/antibodies-controls/primary-antibodies/cd8-%28autostainer-link-48%29-76353?srsltid=AfmBOoqUg2AkZJjeFnwfaMEj3xmqK63MooicNKv6mI7SqyNXaMWCJD3Q).<br>Antibody concentrations were as follows: CD4 1:140; CD8 1:450; FOXP3 1:285, CD68 1:100; Pan-cytokeratin 1:500; CD20 1:280.
Validation	Each primary antibody was validated via manufacturer data and expected tissue localization. We included positive control tissues (tonsil and liver) on each staining run/batch for both multiplex IF and IHC. Exact experimental conditions for optimising antibodies for our study can be found in Methods, subject 'Spatial Proteomics' and in Supplementary Information, Table 1.

Clinical data

Policy information about clinical studies
All manuscripts should comply with the ICMJE guidelines for publication of clinical research and a completed CONSORT checklist must be included with all submissions.

Clinical trial registration	TAILORx Tissue Bank NCT02050750 (CTRIAL-IE 12-30).
Study protocol	A retrospective analysis of tissue bank material from the prospective, randomised controlled study TAILORx. Female ptients had to have been previously registered with the ICORG 06-31 TAILORx trial and participated in trial arms. Patients required adeqeuate tumour tissue available.
Data collection	Clinical variables were taken from trial records and pathology reports from participating hospitals across the Republic of Ireland from December 2013 until December 2023.
Outcomes	(Primary) Disease relapse, endocrine and/or chemotherapy resistance prognosis. A biobank, TMAs and full face sections, allow for the identification of candidate biomarkers prognostic for disease relapse and predictive for endocrine and/or chemotherapy resistance.<br>(Secondary) Signatures/biomarkers. Validation of emerging signature biomarkers.<br>All time frames occurred for the duration of follow-up.

Plants

Seed stocks

Report on the source of all seed stocks or other plant material used. If applicable, state the seed stock centre and catalogue number. If plant specimens were collected from the field, describe the collection location, date and sampling procedures.

Novel plant genotypes

Describe the methods by which all novel plant genotypes were produced. This includes those generated by transgenic approaches, gene editing, chemical/radiation-based mutagenesis and hybridization. For transgenic lines, describe the transformation method, the number of independent lines analyzed and the generation upon which experiments were performed. For gene-edited lines, describe the editor used, the endogenous sequence targeted for editing, the targeting guide RNA sequence (if applicable) and how the editor was applied.

Authentication

Describe any authentication procedures for each seed stock used or novel genotype generated. Describe any experiments used to assess the effect of a mutation and, where applicable, how potential secondary effects (e.g. second site T-DNA insertions, mosiacism, off-target gene editing) were examined.
