# Supplementary material for: Spatial analyses implicate high stromal tumour-infiltrating CD8+ lymphocytes as a negative predictive marker for chemotherapy in estrogen receptor-positive breast cancer
Source: Nat Commun. 2026 Jun 23;17:4863. doi: 10.1038/s41467-026-73432-2 (PMC13291258; doi:10.1038/s41467-026-73432-2)
Supplement: Supplementary file 3 — Transparent Peer Review File [file 41467_2026_73432_MOESM3_ESM.pdf]

nature portfolio

Peer Review File

Spatial-analyses implicate high stromal tumour-infiltrating CD8+ lymphocytes as a negative predictive marker for chemotherapy in estrogen receptor-positive breast cancer

Corresponding Author: Dr Zak Kinsella

This file contains all reviewer reports in order by version, followed by all author rebuttals in order by version.

Version 0:

Reviewer comments:

Reviewer #1

(Remarks to the Author)
This study derived multi-omic spatial data on a large cohort collected within the TAILORx study, a clinical trial which has contributed to tailor the use of chemotherapy in luminal early breast cancer. They showed how TME features, in particular T cell quantification can further refine the identification of patients that should or should not receive chemotherapy in combination with endocrine treatment, a finding that, if further validated has high translational potential. However, the manuscript has a few shortcomings, particularly in the first part, that limit the readability and an easy identification of the main messages. The authors have obtained a quite rich dataset that could be further exploited to derive relevant information.

Major considerations
1. The hypothesis that macrophage/T-helper hubs both facilitate and restrain cytotoxic activity is largely correlative. The work would benefit from a clearer mechanistic narrative possibly supported by spatial transcriptomic co-localization analyses or ligand–receptor inference (e.g., NicheNet, CellPhoneDB).
2. I would suggest starting with providing an overview of the cell populations found in the cohort (i.e. heatmap-like visualization) which could lead to the identification of specific patterns or distinct TME types by unsupervised analysis.
3. Figure 2 - To claim a link between immune infiltration and risk, authors should systematically compare each cell type percentage and/or density across RS groups, and not indirectly, based on the type of treatment received. In general, comparisons between HT and HT+chemo should be replaces with RS defined groups (as in figure 3 but without macrophage-based stratification). These are baseline profiles not affected by the received treatment, while it is of interest to link TME features with tumour features captured by the RS score.
4. Macrophage-based stratification can make sense but I am not convinced should have such a central role. It is quite known that tumour immune infiltration is always involving all main cell types, although quantitative (and spatial) differences could emerge. The authors could aim at a more comprehensive portrait of TME composition across patients.
5. Figure quality is in general poor. Font is too small and sometimes there is no proportion between the amount of info and the space occupied (e.g 2f or 3d vs 2g-i or 3e-f). For axes reporting percentage or density, please consider using a log scale to improve readability. For consistency, keep the color and name of treatments uniform across figures (e.g.: fig 4a and fig 4c, different names and colors).
6. Page 10, lines 288 and 295: wrong referenced figure panel, should be 3D instead of 4D.
7. A formal test of interaction is mentioned (ΔLR χ^2), but effect sizes and confidence intervals should be presented directly in the text and main figures for transparency.
8. Translationally, the main finding is the link between CD8 T cell and poor outcome in patients interm-RS receiving CT. From the other analysis the authors indicate that such higher infiltration is linked to a process of recruitment but suppression. However, such TME is somehow pro-tumoral when CT is administered. Taking advantage of the rich data generated, can the authors indicate possible mechanisms or relevant signals? Can multivariate models, including multiple cell types or spatial features (or TME types accounting for its full composition) perform any better than simple CD8 T cell quantity/density?
9. In figure 4, might be useful to show whether the described effect is present in both pre- and post-menopausal patients
10. When transcriptomic data is used, the expression of several markers could indicate their up/down regulation or changes in the prevalence of cell types expressing them. What's the author's interpretation or did they adjust for this?
11. Most distances (e.g. Figure 3 b-c) are in the range of one cell size or less, is this correct? How did the authors control for

the segmentation process and quality? More details could be provided in the methods and with representative images
12. Since data from multiple experiment/omics are used, it should be better clarified in the text which data are used/described in each analysis
13. Please rephrase the results description in the abstract, it is unclear and hard to follow
14. Version of software and packages used should be indicated in the methods
15. External validation is currently missing. Although the orthogonal validation using whole-tissue resections is methodologically strong, it remains an internal validation. The authors should discuss limitations of internal validation and the need for independent cohorts for clinical translation.
16. The rationale for FDR<0.1 instead of 0.05 should be justified, especially for survival and treatment–biomarker interaction analyses that directly inform clinical interpretation.
17. Data Availability: for transparency, raw spatial data (e.g., GeoMx count matrices and image masks) should be deposited in GEO or Zenodo, as expected by Nature journals.
18. The term "chemotherapy inferiority" should be used cautiously. It would be preferable to state that chemotherapy "did not confer benefit" or was "associated with poorer outcome" in high-CD8+ patients.
19. Please define abbreviations of sTIL and iTIL at first mention
20. Specify how the cutoff for "high" versus "low" for macrophage and CD8 density was chosen (median, tertile, ROC-based?)

Reviewer #2

(Remarks to the Author)
In their manuscript "Spatial-Immune Multi-omics Refines Prognostication in Early-Stage Estrogen Receptor-Positive Breast Cancer" Kinsella et al present a large spatial transcriptomics/proteomics (Nanostring GeoMx) centered study to improve on ER+HER2- breast cancer patient treatment stratification. In particular they focus on the intermediate RS group where treatment decisions are currently not optimally supported. Crucially, Kinsella et al describe a biomarker (stromal cytotoxic T cell density) derived from the spatial data, that is significantly associated with intermediate RS patient's response to chemotherapy. While independent validation is necessary, the presented evidence across a large discovery cohort of ~400 patients and another ~500 validation patients is compelling and an important step in using spatial transcriptomics/proteomics for clinical purposes to the benefit of patients. However, despite the impressive design and obvious potential of the study there is room for improvement especially in the communication and presentation of the results (see comments below).

1. Unintuitive manuscript structure: Seemingly the most relevant result (biomarker to stratify the intermediate group) is mentioned last and seems buried in a lot of exploratory analysis. More intuitive would be to lead with the crucial biomarker finding and then follow with "targeted" exploratory analysis to investigate and explain the biomedical reasons/plausibility of the biomarker finding.
2. Generally, the manuscript could be written in a more concise manner helping the reader more to "see the point" which is currently too often buried in mere reporting. On the other hand, conclusions sometimes seem to come out of the blue. E.g. Line 188: "These data suggest that higher risk diseases may globally shift macrophage toward a more suppressive state in which incumbent cytotoxic T-cells are not primed for tumour killing by antigen display, therefore potentially increasing T-cell exhaustion.", when the two paragraphs before seemed to assess all risk groups and seemed to report mostly about T-Macrophage distances and macrophage phenotypes (without interpretation).
3. Better define crucial terms and concepts. In particular iTIL, sTIL, and "stromal". Since these presumably refer to crucial spatial distinctions which define the reported biomarkers, it is necessary to properly define them upfront and not leave the reader to deduce from prior knowledge and context.
4. Causal inference sometimes seems not well supported e.g. line 176 "suggesting that macrophage-dominated environments can facilitate cytotoxic T-cell entry to epithelia in lower risk tumours; a pattern attenuated in higher risk disease." – why not the other way around (cytotoxic T cells or their effect attract macrophages) or a third independent factor causes both? Or line 206: "This analysis revealed that the epithelial segment markedly upregulated genes in the antigen presentation module (HLA-DRA, HLA-DRB1, CIITA)." Why not interpret as epithelia with higher antigen presentation attract more T cells?
5. Cytotoxic T cell density seems to be the crucial biomarker why is spatial necessary to detect that (related to point 3). And if not necessary, could the stratification also be done on bulk RNAseq with cell type proportion inference (as estimate of density)? This would open up additional large cohorts like TCGA.
6. Improve figures: most figures have too small text even though there would be space for larger text. E.g. Fig 2g,h,i the heatmap part could be much smaller making space for larger text. But also other figures suffer from similar problems. In the compiled PDF most text in the figure is not legible – it was necessary to manually go back to the individual figures.
7. Suggestion to additionally "validate" the biomarker finding through an inverse analysis: Compare sTIL high vs low in chemo + and chemo - respectively and check if sTIL high have worse survival in chemo + but same in chemo –.
8. Line 256 : "The High RS had significantly shorter distances of B-cells (median 16.7μm), T-helper cells (median 16.2μm), and cytotoxic T-cells (median 15.9μm) from macrophage." – compared to what? The other risk groups? Something seems to be missing.
9. Paragraph at line 299 seems out of order based on the figures cited (Figure 3 panels cited after figure 4 panels).
10. Line 326: p=0.017, FDR<0.01 FDR cannot be smaller than p-value – likely typo maybe stats problem?
11. Line 356 "For patients with an Intermediate RS, the association of cytotoxic T-cell density with outcome was likely confounded by the randomisation of administered treatment." Very confusing statement. Why is it relevant in this context and why "likely confounded" – randomization should prevent "confounding". Was it not done properly? In this case it seems there is either a confounding factor or there is not.

Reviewer #3

(Remarks to the Author)
General comments

This paper examines ER^+/$HER2^-$ early breast cancer using spatial transcriptomics (GeoMx DSP), multiplex IF, and IHC in a large TAILORx cohort (n=577; 410 with ST; 442 with spatial proteomics) to identify immune–spatial features associated with recurrence risk and chemotherapy benefit. The manuscript is very difficult to read and contains several contradictions. The authors should temper their claims, as most findings relate to the TME in the primary tumor rather than distant micrometastatic sites, which are the targets of adjuvant endocrine therapy and chemotherapy. Furthermore, all results rely on a single dataset with no external validation (e.g. applying deconvolution on publicly available bulk data).

“High sTILs CD68” - Macrophages are not lymphocytes.

Show all dots on boxplots. Add p-values to boxplots.

Display actual FDRs and use consistent cut-offs. FDR<.1 is too lenient. p-values should be shown with 1 or 2 significant digits, not 3 (e.g. not p=0.00237 as in the text) nor 0 (e.g. p=0.000 in fig 4a).

M&Ms are a bit generic at times (e.g. "Differences in values between two categories was assessed using pairwise Mann-Whitney or Student’s t-test" - why 2 methods? I don't think any of the two was actually applied).

Fonts on figures are way too small. Figures are very hard to read, which is made worse by the fact that some panels are bitmaps (not vector graphics). Some panels are almost impossible to read (e.g. S3c), even fully zoomed. Panel S4e is an extreme example - extremely small numbers in big boxes. Why this hatred towards the readers?

Comments on specific analyses

For the figures on distances (e.g. 2B) the distribution of individual cells is displayed. The experimental unit however is the sample, so statistics cannot be performed on such data, and to the authors credit it is indeed not performed. This is replaced by an effect size consideration, which I think is not good enough.
The distances can easily be summarized for each sample (e.g. the median, the % below a certain cut-off, whatever). Proper statistics can then be performed using that value.
I don't understand negative distances for macrophages (2c).
In general you would expect distance to be smaller with increasing amount of the cell types compared. This is not discussed.

Panels 2c-2e are not discussed in the text.

Forest plots are hard to read. P-values are at times cut on the right of the panel (e.g. Macrophages -> CD8 in 2f).

Fig 2f, "Facilitation of sTILs CD68 to iTILs CD68" is a really bad x-axis label.
The model is impossible to understand without checking the details in the M&Ms, where it is hard to find (in the middle of a 2.5 pages-long "statistical analysis" section).

Fig 2g - not clear if expression is in tumor or stroma.
Done as a regression analysis, but would be more natural to use correlations. Correlations would allow to use non-parametric testing as well as to actually compare the correlations across treatment arms. Also, scatterplots should be shown for all pairs in supplementary and probably the most interesting ones in the main figure.

Fig 3a - the lower line of the x label is actually the y labels. This is very confusing.
The insert (density subplot) overlaps with some dots and might hide others.

Fig 3d - it should be described how the module scores were computed (mean of the genes?).
It is stated that the model controls for immune densities, however immune densities (e.g. CD8 density) are shown - this is explained in the M&Ms but not in text or legend and is confusing.
Also, my understanding is that the regression uses a distance computed by sample. How was this distance computed? And why was it not used in the other analyses?
I don't understand the R2 analysis. All error bars overlap anyway.

Fig 3e - x label is "treatment", I assume it should be "Oncotype DX"

Fig 3f - use log x-axis. P-values are cut on the right (not displayed). There is no way that the p-values reported have FDR < 10% as stated (60 comparisons, most significant p-values are ~ 1% and there are not many of those).

Fig 4a - x axis should be in log.
My understanding is that this analysis was done on the randomized arms because of "Investigating immune variables across randomised arms (Fig.4A)" in the text, however there is nothing about this in the figure legend.
It is a surprising that the error bars are so much larger in the HT arm. The reason for this seems to be that patients with

HT+CT have much worse prognosis than patients with HT only (fig 4c). Was this really randomized?
I find the result from the interaction test surprising. Usually the p-value from the interaction can be roughly estimated by comparing the CIs in the two groups. For CD8 density those CIs overlap - the point estimate for CT+HT is actually in the CI of HT. However, the interaction test is given as significant (p=.007). Was that done on the same data?
Also, the interaction test should be corrected for multiple testing, as clearly those were hand picked as the most likely to be significant.

Fig 5 - I think that claiming that chemotherapy has a negative net benefit is pretty wild (fig 5g).
Patients were not randomized, so we can expect patients receiving HT+CT to have a worse disease. So in general CT+HT should be worse than HT, not because treatment is bad but because of patient selection.
Lots of cytotoxic Tcells -> higher Ki67 (on average). So those with low density will be much more likely to be lumA, for who CT is not necessarily given but can help. Which may be what is seen here.

Reviewer #4

(Remarks to the Author)
I co-reviewed this manuscript with one of the reviewers who provided the listed reports. This is part of the Nature Communications initiative to facilitate training in peer review and to provide appropriate recognition for Early Career Researchers who co-review manuscripts.

Version 1:

Reviewer comments:

Reviewer #1

(Remarks to the Author)
The manuscript has substantially improved following revision. The authors have adequately addressed the concerns and suggestions raised during the initial review.

Reviewer #2

(Remarks to the Author)
My comments have largely been addressed and it is clear that substantial work has been done in the revision, generally strengthening the manuscript. However, I would urge the authors to carefully go through text and figures again to make both more reader friendly. Even though there has been some improvement often text is still too tiny (e.g. many figure legends in Fig 4). It should be easily fixable by editing manually in inkscape for example.
In the text, especially the new passages there seem to be some “careless mistakes”. Eg. “Using spatial proteomic data (n=440), we discovered that no TME (stromal tumour-infiltrating lymphocyte: sTIL) phenotype density was significantly different across the RS except for sTIL T-regulatory cells […] “(stromal tumour-infiltrating lymphocyte: sTIL)” seems to be at the wrong spot or if it is at the right spot it is very confusing the way it is written.

Reviewer #3

(Remarks to the Author)
The authors addressed several of my and other reviewers' comments and significantly improved their manuscript. I do not have additional comments.

Reviewer #4

(Remarks to the Author)
I co-reviewed this manuscript with one of the reviewers who provided the listed reports. This is part of the Nature Communications initiative to facilitate training in peer review and to provide appropriate recognition for Early Career Researchers who co-review manuscripts.

Open Access This Peer Review File is licensed under a Creative Commons Attribution 4.0 International License, which permits use, sharing, adaptation, distribution and reproduction in any medium or format, as long as you give appropriate credit to the original author(s) and the source, provide a link to the Creative Commons license, and indicate if changes were made.
In cases where reviewers are anonymous, credit should be given to 'Anonymous Referee' and the source.
The images or other third party material in this Peer Review File are included in the article's Creative Commons license, unless indicated otherwise in a credit line to the material. If material is not included in the article's Creative Commons license and your intended use is not permitted by statutory regulation or exceeds the permitted use, you will need to obtain permission directly from the copyright holder.
To view a copy of this license, visit https://creativecommons.org/licenses/by/4.0/

Author Rebuttal Comments:
Spatial-Immune Multi-omics Refines Prognostication in Women with Early-Stage Estrogen Receptor-Positive Breast Cancer

REVIEWER COMMENTS

N.B. During re-analysis for revision, we identified duplicate records in the merged clinical-proteomic dataset. We have since removed the outliers (n=2. Updated proteomic data n=440) and all analyses using proteomic data were re-run on the deduplicated dataset. Our conclusions remain unchanged, effect sizes were similar, and all figures and supplementary materials have been updated to reflect the change.

Reviewer #1 [breast cancer](Remarks to the Author):

This study derived multi-omic spatial data on a large cohort collected within the TAILORx study, a clinical trial which has contributed to tailor the use of chemotherapy in luminal early breast cancer. They showed how TME features, in particular T cell quantification can further refine the identification of patients that should or should not receive chemotherapy in combination with endocrine treatment, a finding that, if further validated has high translational potential. However, the manuscript has a few shortcomings, particularly in the first part, that limit the readability and an easy identification of the main messages. The authors have obtained a quite rich dataset that could be further exploited to derive relevant information.
Reply: We would like to thank the reviewer for reading our manuscript and providing the opportunity to amend and accommodate their comments to improve our submission.

Major considerations
1. The hypothesis that macrophage/T-helper hubs both facilitate and restrain cytotoxic activity is largely correlative. The work would benefit from a clearer mechanistic narrative possibly supported by spatial transcriptomic co-localization analyses or ligand–receptor inference (e.g., NicheNet, CellPhoneDB).

Reply: We agree that the work would benefit from a clearer mechanistic narrative with ligand-receptor inference and so have performed an additional hypothesis-generating analysis using OmniPath and the database of ligands and receptors catalogued in that work. While we did explore the using of NicheNet and CellPhoneDB, we found that these

tools required cell type-resolved expression data found commonly with single-cell data. As such, we were cautious to introduce this to our spatially resolved bulk data, to avoid overinterpretation of directionality/causality. We therefore performed a differential expression analysis of OmniPath-curated ligand-receptor genes by TME/EPI states, finding patterns consistent with our aforementioned recruitment and checkpoint signalling.

Updates to the manuscript incorporating ligand-receptor analysis can be found in pages 9-11, under the subheading "Immune-hot Epithelia Co-Occur with Immunoregulatory Stroma". We have also updated the materials and methods: "Ligand-Receptor Analyses", page 34.

2. I would suggest starting with providing an overview of the cell populations found in the cohort (i.e. heatmap-like visualization) which could lead to the identification of specific patterns or distinct TME types by unsupervised analysis.

Reply: We have updated the manuscript to begin by incorporating these suggestions. The results section is now introduced with an overview of cell populations across the Oncotype Dx RS and followed by patterns discovered using unsupervised clustering. These data can be found on page 6, under the title "Outlining the Tumour-Immune Microenvironment of the Oncotype Dx RS".

We also take these TME/EPI types further throughout our revised manuscript, including an analysis across states, in the results subsection on page 9 "Immune-hot Epithelia Co-Occur with Immunoregulatory Stroma". And update our final figure/analysis to incorporate the TME and EPI states, on page 21 "Spatial and Transcriptomic Signatures Link Immune States to Exclusion, Checkpoint Activity, and T-cell Exhaustion".

3. Figure 2 - To claim a link between immune infiltration and risk, authors should systematically compare each cell type percentage and/or density across RS groups, and not indirectly, based on the type of treatment received. In general, comparisons between HT and HT+chemo should be replaces with RS defined groups (as in figure 3 but without macrophage-based stratification). These are

baseline profiles not affected by the received treatment, while it is of interest to link TME features with tumour features captured by the RS score.

Reply: We agree with this comment and have included this systematic comparison across both stroma/TME and epithelia, by the ODx RS, in an updated Figure 2B and 2C (*Page 8*).

4. Macrophage-based stratification can make sense but I am not convinced should have such a central role. It is quite known that tumour immune infiltration is always involving all main cell types, although quantitative (and spatial) differences could emerge. The authors could aim at a more comprehensive portrait of TME composition across patients.

Reply: We have pared back macrophage-based stratification across the manuscript to favour the comprehensive patterns found during the above unsupervised clustering analysis. In later figures (e.g. updated Figure 6), while we found macrophage density to remain an important variable for defining TME transcriptomic activity, we temper our approach by not foregrounding a macrophage-based stratification. Instead, we favour stratification by unsupervised clusters, and investigate the transcriptomic correlates of increasing macrophage density across these states. Changes made can be found in results sections 'Outlining the Tumour-Immune Microenvironments of the Oncotype Dx RS' (page 6), 'Immune-Hot Epithelia Co-Occur with Immunoregulatory Stroma' (page 9), and 'Spatial and Transcriptomic Signatures Link Immune States to Exclusion, Checkpoint Activity, and T-cell Exhaustion' (page 21).

5. Figure quality is in general poor. Font is too small and sometimes there is no proportion between the amount of info and the space occupied (e.g 2f or 3d vs 2g-i or 3e-f). For axes reporting percentage or density, please consider using a log scale to improve readability. For consistency, keep the color and name of treatments uniform across figures (e.g.: fig 4a and fig 4c, different names and colors).

Reply: We agree and apologise that figure legends were too small and, in some cases, illegible. Therefore, we have made changes across all figures to improve legibility. Axes reporting density (e.g. updated Fig.2B and 2C, page 8) have been changed to use log scales. Colours used in figures (e.g. updated Fig.4C and 4D page 16 , Fig.5C page 18) have been updated to be uniform for consistency.

6. Page 10, lines 288 and 295: wrong referenced figure panel, should be 3D instead of 4D.

Reply: Thank you for pointing this out. This has been altered due to changes made in manuscript structure and should now be fixed.

7. A formal test of interaction is mentioned (ΔLR χ^2), but effect sizes and confidence intervals should be presented directly in the text and main figures for transparency.

Reply: Effect size and confidence intervals are now shown in both main figures and text body when reporting ΔLR χ^2. While the likelihood ratio test does not itself produce effect sizes and confidence intervals, we have taken the hazard ratio and 95%CI from the Wald test from the formal test of interaction and report both for transparency (e.g. Figure 4, page 17. Figure 5, page 20. and all associated text).

8. Translationally, the main finding is the link between CD8 T cell and poor outcome in patients interm-RS receiving CT. From the other analysis the authors indicate that such higher infiltration is linked to a process of recruitment but suppression. However, such TME is somehow pro-tumoral when CT is administered. Taking advantage of the rich data generated, can the authors indicate possible mechanisms or relevant signals? Can multivariate models, including multiple cell types or spatial features (or TME types accounting for its full composition) perform any better than simple CD8 T cell quantity/density?

Reply: We thank the reviewer for their comments and agree with their concerns regarding independent prognostic information and relevant mechanisms. We have therefore updated the manuscript to include multivariable models to include TME and EPI type states (page 11 text, and Supplementary FigsS6, S7). We have also updated the previous Figure 2, where these states were first introduced, to include GSEA that may highlight pathways and mechanisms indicating relevant signals versus cold tumours (page 8, Fig.2G and 2H), and differential expression analysis across TME and EPI states in updated Figure 3 (page 12, Fig.3C and 3D). We take this further in a revised results section "Immune-Hot Epithelia Co-Occur with Immunoregulatory Stroma' (page 9), and

‘Spatial and Transcriptomic Signatures Link Immune States to Exclusion, Checkpoint Activity, and T-cell Exhaustion” (page 21-24) and corresponding Figure 6 (page 25). Together, these data indicate that the poorer outcome of patients on chemotherapy may be due to a dysfunctional, chronically stimulated, remodelling-oriented stroma. These environments appear to exclude an immune response, as the epithelia upregulates chemotactic signals and antigen presentation machinery yet spatial analysis show the bulk of immune infiltrates accrue in the peri-tumoural space. Moreover, in these states, we find that as CD8 density increases in the epithelia, there is a significant upregulation of cytotoxicity genes paired with CTLA4 and CXCL13, indicating potentially dysfunctional activity that may be ineffective in tumour clearance. This mirrors recent single-cell work in luminal breast cancer recently (Tietscher *et al. Nature Comms. 2023.* https://doi.org/10.1038/s41467-022-35238-w). We position our claims as exploratory and suggestive, and highlight the need for further exploration in larger cohorts with deeper immune profiling.

Of note, similar hallmarks to those outlined in our GSEA analysis have been recognised in a group of responders to neoadjuvant immune checkpoint blockade and chemotherapy in a very recent publication of an ER+HER2- pilot study (Waks *et al. Nature Comms. 2025.* https://doi.org/10.1038/s41467-025-66667-y). We now update our discussion to include this recent publication.

Regarding survival analysis, we found that a relatively immune-hot epithelia (EPI-3) is indeed a prognostic indicator in the IntRS receiving HT+CT. We have included in supplement a forest plot showing multivariable Cox regression and nested survival analyses of EPI-3, sTIL CD8 density, and iTIL CD8% (Supplementary Fig.S9), highlighting no independent prognostic information provided by sTIL CD8 density or iTIL CD8% to EPI states and vice-versa. We have mentioned these data in results on page 11 and page 14.

9. In figure 4, might be useful to show whether the described effect is present in both pre- and post-menopausal patients

Reply: We agree and have examined whether the association of sTIL CD8 density with poorer outcome is present in both pre/perimenopausal and postmenopausal Intermediate RS patients overall, and across randomised treatment arms. We now

include Kaplan-Meier curves stratified by menopausal status (Supplementary Fig.S11). The direction of effect is consistent across strata. Higher sTIL CD8 density is associated with poorer iDFS. This reaches statistical significance in the postmenopausal stratum (log-rank p=0.035) and shows the same direction but does not reach significance in the pre-/peri-menopausal stratum (log-rank p=0.097), consistent with reduced power after stratification. Cox estimates were, however, comparable in magnitude, with wider confidence intervals in the pre-/peri-menopausal group (Low vs High: Pre-/peri-menopausal HR = 0.27, 95%CI 0.05-1.41; Postmenopausal: HR = 0.27, 95%CI 0.07-1.0).

We have also updated the body text to highlight this information, on page 15.

10. When transcriptomic data is used, the expression of several markers could indicate their up/down regulation or changes in the prevalence of cell types expressing them. What's the author's interpretation or did they adjust for this?

Reply: We agree that in compartment-resolved transcriptomic data, changes in several markers can reflect either altered gene regulation within cells or shifts in the abundance of cell types expressing those genes. To mitigate the latter, in all models linking immune gene module activity to cytotoxic T-cell/macrophage density, we adjusted for all other cell densities within the same compartment (sTIL for TME, iTIL for epithelia), so that our estimated associations reflect gene activity conditional on the cellular composition rather than there simply being more immune cells. We have updated the methods to more explicitly state this, on page 34-35, in the subheading "Gene Modules and Regression", and in our legend for Figure 6, on page 25.

11. Most distances (e.g. Figure 3 b-c) are in the range of one cell size or less, is this correct? How did the authors control for the segmentation process and quality? More details could be provided in the methods and with representative images

Reply: We have updated our distance analysis (now Figure 6B-D, page 25) to instead show these data across TME-EPI states. In this change, we now include an analysis to show the coupling between sTIL and iTILs (Fig.6C) and respective example images of 250x250μm ROIs from cores annotated by each state (Fig.6D). Regarding segmentation

quality, we utilised a peer-reviewed method (StarDist) that has been optimised for cell segmentation of fluorescent images and performed visual quality control with an in-house research pathologist (Author list: C.A.G). We have updated the methods to provide more clarity around this methodology: “Nuclear detection in the DAPI channel was performed using the StarDist (46) QuPath plugin, or watershed cell detection in the haematoxylin channel (Supplementary Table S4-S5). Cell segmentation quality control was performed with a pathologist (C.A.G).”

Distances are nearest-neighbour 2D Euclidean values between cell centroids (μm). We found that immune cells tended to cluster together around the peri-tumoral space, leading to small distances. This is reflected in the updated example images in Figure 6D (page 25). At the patient level (Figure 6B), signed distances from a tumour mask remain within ~0-25μm, indicating consistency across patients and that this finding is not dominated by few ROIs with dense cellularity. We have updated the methods to reflect this (page 36, subheading “Digital Pathology and Phenotype Scoring”): “Spatial analyses were performed using “Detection centroid distances 2D” script commands within QuPath. This command computes the 2D Euclidean distance (μm) between the centroid of a cell of a source class to the nearest centroid of a target class. These data were exported and analysed further in R Studio, and summarised as the median distance per patient.”

12. Since data from multiple experiment/omics are used, it should be better clarified in the text which data are used/described in each analysis

Reply: We have updated the manuscript to include brief sentences within each results subheading explaining which data are used in the proceeding analyses. We also update the materials and methods with a dedicated subheading to outline the subsets used throughout the paper (*Datasets*, page 30-31).

13. Please rephrase the results description in the abstract, it is unclear and hard to follow

Reply: We have updated our abstract to reflect data derived from TME and EPI states and refine the wording to be easier to follow.

"Despite a strong 5-10 year prognosis, in ER^+HER2^- breast cancer relapses are common beyond 10 years. Genomic assays employed to determine recurrence risk at diagnosis are still ambiguous for a significant proportion of patients with Intermediate risk (i.e. Oncotype Dx Recurrence Score (RS) 16-25). Using spatial immune multi-omics, we show distinct immune states across stroma and epithelia, ranging from immune-hot (30% of stroma, 28% of epithelia) to truly immune-cold (20% of stroma, 28% of epithelia), that are not reliably coupled. Inflamed stroma appear remodelling-oriented, expressing exhaustion-related and inhibitory/checkpoint transcripts (CTLA4, TIGIT, CD96), whereas inflamed epithelia are associated with antigen presentation, checkpoints (CTLA4), and exhaustion genes (CXCL13). In the randomised Intermediate RS, a treatment-biomarker interaction indicated poorer outcome on chemotherapy at higher stromal $CD8^+$ density (ΔLR-χ^2: 6.79, p=0.009, q=0.018), which was validated orthogonally on whole-resection specimens of Intermediate RS patients (ΔLR-χ^2: 8.90, p=0.003). Using the candidate biomarker suggested a potential treatment change for up to 50% of Intermediate RS patients."

14. Version of software and packages used should be indicated in the methods

Reply: We have updated all methods and materials to reflect version numbers of software and packages used.

15. External validation is currently missing. Although the orthogonal validation using whole-tissue resections is methodologically strong, it remains an internal validation. The authors should discuss limitations of internal validation and the need for independent cohorts for clinical translation.

Reply: We agree that external validation is missing and that this is a crucial point for clinical translatability. We are currently working on getting access to cohorts with similar randomisation of the Intermediate RS, though such cohorts are rare and difficult to access externally. We have stressed this need in our results sections (e.g. page 15), "Thus, these results should be interpreted with caution and require external validation in a larger cohort of chemotherapy-randomised patients with an Intermediate RS before optimum thresholds can be generalised to practice."

And discussed limitations on page 29: “It is a shortcoming of our study that we have no external validation data. Prospective clinical cohorts with treatment randomisation of the Intermediate RS are very sparse. As such, for clinical adoption, it may be necessary to validate these findings in the larger TAILORx trial. A comprehensive validation is required before clinical adoption of the proposed biomarker – and the ramifications of treatment change for a marked population of patients with an Intermediate RS - can be weighed and seriously discussed. Without this, optimal thresholds cannot be established, which will be required to precisely understand whether and for whom chemotherapy is truly inferior for the biomarker population.”

16. The rationale for FDR<0.1 instead of 0.05 should be justified, especially for survival and treatment–biomarker interaction analyses that directly inform clinical interpretation.

Reply: We used FDR < 0.10 as primarily a discovery/exploratory threshold, to nominate immune and spatial features in a setting with correlated tests and limited survival events. A more conservative FDR of 0.05 may increase false negatives in this setting. To compensate for this, in our full clinical interaction analyses we show full effect sizes and 95%CI, report interaction p-values, and provide sensitivity analyses so that the magnitude and robustness of the effect is transparent. We furthermore frame our findings as a promising indication and observational, stressing the requirement for a comprehensive external validation in the larger TAILORx trial before clinical adoption can be discussed.

17. Data Availability: for transparency, raw spatial data (e.g., GeoMx count matrices and image masks) should be deposited in GEO or Zenodo, as expected by Nature journals.

Reply: our working Zenodo repository can be found at the below DOI, and we have updated this with GeoMx count matrices and image masks.

https://doi.org/10.5281/zenodo.17288210

18. The term “chemotherapy inferiority” should be used cautiously. It would be

preferable to state that chemotherapy "did not confer benefit" or was "associated with poorer outcome" in high-CD8+ patients.

Reply: We have tempered our language on this recommendation, across all parts of the manuscript.

19. Please define abbreviations of sTIL and iTIL at first mention

Reply: We have updated the manuscript to accommodate this change.

20. Specify how the cutoff for "high" versus "low" for macrophage and CD8 density was chosen (median, tertile, ROC-based?)

Reply: For survival analysis with Kaplan-Meier plots, high versus low cutoff was defined by median density. We have made this more obvious in the body text. For Cox modelling, we used continuous percentages and/or densities, and have also updated the text to reflect this. E.g:

Page 13: "We next investigated whether any individual immune variable could recapitulate the finding of poorer outcome on chemotherapy seen in EPI-3. We therefore modelled continuous immune counts and densities across the Oncotype Dx RS."
Page 14: "Using a median cutoff as an illustration for survival analysis, high sTIL $CD8^+$ density (>median, ~30 cells/mm^2) had significantly poorer survival than low density in the chemoendocrine arm."

Page 14: "We subsequently performed an inverse analysis (Fig.3C). We divided the Intermediate RS into high vs low by median iTIL CD8% (Fig.3C) and sTIL $CD8^+$ density (Fig.3D), and investigated the 15yr iDFS difference by received treatment."

Reviewer #2 [spatial data](Remarks to the Author):

In their manuscript "Spatial-Immune Multi-omics Refines Prognostication in Early-Stage Estrogen Receptor-Positive Breast Cancer" Kinsella et al present a large spatial transcriptomics/proteomics (Nanostring GeoMx) centered study to improve on ER+HER2- breast cancer patient treatment stratification. In particular they focus on the intermediate RS group where treatment decisions are currently not optimally supported. Crucially, Kinsella et al describe a biomarker (stromal cytotoxic T cell density) derived from the spatial data, that is significantly

associated with intermediate RS patient's response to chemotherapy. While independent validation is necessary, the presented evidence across a large discovery cohort of ~400 patients and another ~500 validation patients is compelling and an important step in using spatial transcriptomics/proteomics for clinical purposes to the benefit of patients. However, despite the impressive design and obvious potential of the study there is room for improvement especially in the communication and presentation of the results (see comments below).
Reply: We would like to thank the reviewer for reading our manuscript and providing the opportunity to amend and accommodate their comments to improve our submission.

1. Unintuitive manuscript structure: Seemingly the most relevant result (biomarker to stratify the intermediate group) is mentioned last and seems buried in a lot of exploratory analysis. More intuitive would be to lead with the crucial biomarker finding and then follow with "targeted" exploratory analysis to investigate and explain the biomedical reasons/plausibility of the biomarker finding.

Reply: We agree with this suggestion and so have updated the manuscript in several ways to accommodate. We firstly have introduced two new figures (Figure 2, Figure 3) at the beginning of the results section that outlines the overarching immune environment, performs hierarchical clustering to find TME and epithelia states/patterns, and produces analyses across states to highlight potential mechanisms associated with each state. We have moved the biomarker finding to Figure 4 and 5, and conclude with a deeper analysis that explains the plausibility of the finding. We find this structure to be more intuitive.

2. Generally, the manuscript could be written in a more concise manner helping the reader more to "see the point" which is currently too often buried in mere reporting. On the other hand, conclusions sometimes seem to come out of the blue. E.g. Line 188: "These data suggest that higher risk diseases may globally shift macrophage toward a more suppressive state in which incumbent cytotoxic T-cells are not primed for tumour killing by antigen display, therefore potentially increasing T-cell exhaustion.", when the two paragraphs before seemed to assess all risk groups and seemed to report mostly about T-Macrophage distances and macrophage phenotypes (without interpretation).

Reply: On reordering the manuscript and including new figures, we have taken the time to sharpen our writing style which we hope is now more concise, avoiding conclusions that arise without prior justification.

3. Better define crucial terms and concepts. In particular iTIL, sTIL, and "stromal". Since these presumably refer to crucial spatial distinctions which define the reported biomarkers, it is necessary to properly define them upfront and not leave the reader to deduce from prior knowledge and context.

Reply: iTIL and sTIL have been defined on first use and we have stuck with these terms throughout the manuscript to prevent confusion regarding crucial concepts. Since macrophage are not lymphocytes, we refrain from using xTIL and instead describe them as 'stromal' or 'epithelial'.

4. Causal inference sometimes seems not well supported e.g. line 176 "suggesting that macrophage-dominated environments can facilitate cytotoxic T-cell entry to epithelia in lower risk tumours; a pattern attenuated in higher risk disease." – why not the other way around (cytotoxic T cells or their effect attract macrophages) or a third independent factor causes both? Or line 206: "This analysis revealed that the epithelial segment markedly upregulated genes in the antigen presentation module (HLA-DRA, HLA-DRB1, CIITA)." Why not interpret as epithelia with higher antigen presentation attract more T cells?

Reply: We have updated the manuscript to tone down causal claims and reduce the directionality of our language to prevent overinterpretation, where appropriate.

5. Cytotoxic T cell density seems to be the crucial biomarker why is spatial necessary to detect that (related to point 3). And if not necessary, could the stratification also be done on bulk RNAseq with cell type proportion inference (as estimate of density)? This would open up additional large cohorts like TCGA.

Reply: Spatial allows for the separation of analysis compartments beyond what is feasible with a bulk density measurement, providing granularity on where the accruing density is primarily located. Spatial profiling is necessary here because the predictive signal we detected is compartment specific. Stromal (sTIL) CD8 density and epithelial (iTIL) CD8% are not interchangeable and treating them as a single "bulk CD8" measure would mix biologically distinct contexts. In our data, stromal CD8 density captures peri-tumoural immune accumulation (examples in updated Fig.6B-D. and the TME/EPI

immune states), and this spatial context is what yields a more robust stratification than a compartment-agnostic proxy. Bulk RNA-seq (even with deconvolution) can estimate overall CD8 abundance, but it cannot recover where the infiltrate resides (stromal vs epithelial) nor the associated spatial immune architecture that underpins the biomarker.

Secondly, we performed an exploratory analysis using CD8A in RNAseq data of TCGA, which did not reproduce a prognostic interaction. This was to be expected, as the premise of our biomarker finding hinges on the genomic context (i.e. the Intermediate RS) and the randomisation of the Intermediate RS to adjuvant therapies. While we explored using the Bioconductor package *genefu*, there is no randomisation of treatment in TCGA patients. Randomisation was necessary to discover an interaction of CD8 with treatment, and from this a predictive biomarker of chemotherapy inferiority.

6. Improve figures: most figures have too small text even though there would be space for larger text. E.g. Fig 2g,h,i the heatmap part could be much smaller making space for larger text. But also other figures suffer from similar problems. In the compiled PDF most text in the figure is not legible – it was necessary to manually go back to the individual figures.

Reply: We agree with the reviewer that our figures were too small and difficult to read. We have reformatted all figures with larger font sizes and find them to be much more legible in the compiled PDF.

7. Suggestion to additionally "validate" the biomarker finding through an inverse analysis: Compare sTIL high vs low in chemo + and chemo - respectively and check if sTIL high have worse survival in chemo + but same in chemo –.

Reply: We agree and have updated the manuscript to be more explicit about our using an inverse analysis. This can now be found on page 14:
"We subsequently performed an inverse analysis (Fig.4C-D.). We divided the Intermediate RS into high vs low by median iTIL CD8% (Fig.4C) and sTIL $CD8^+$ density (Fig.4D), and investigated the 15yr iDFS difference by received treatment."

8. Line 256 : "The High RS had significantly shorter distances of B-cells (median 16.7µm), T-helper cells (median 16.2µm), and cytotoxic T-cells (median 15.9µm)

from macrophage." – compared to what? The other risk groups? Something seems to be missing.

Reply: As we have updated the manuscript to focus on unsupervised clusters, the distance analysis has now changed from comparing across Oncotype Dx RS categories to TME and EPI states (Fig.6B-C.). Furthermore, we have updated the manuscript to be more explicit when mentioning these data to which group the others are being compared.

9. Paragraph at line 299 seems out of order based on the figures cited (Figure 3 panels cited after figure 4 panels).

Reply: This has been corrected. Thank you for pointing this out.

10. Line 326: $p=0.017$, FDR<0.01 ▯ FDR cannot be smaller than p-value – likely typo maybe stats problem?

Reply: This typo has been corrected. Thank you for pointing this out.

11. Line 356 "For patients with an Intermediate RS, the association of cytotoxic T-cell density with outcome was likely confounded by the randomisation of administered treatment." Very confusing statement. Why is it relevant in this context and why "likely confounded" – randomization should prevent "confounding". Was it not done properly? In this case it seems there is either a confounding factor or there is not.

Reply: We agree that this was confusing and the reviewer is correct to be concerned about our wording. At first pass, we meant to highlight that an association of a biomarker with treatment may be missed when analysing the Intermediate RS as a whole rather than across randomised arms. Therefore, we have updated this to be more precise and mitigate confusion, on page 13:

"However, for patients with an Intermediate RS, the underlying association of immune density with outcome could be modified and disguised by the type of treatment regimen administered. We therefore investigated by randomised treatment arm (Fig.4)."

Reviewer #3 [breast cancer](Remarks to the Author):

General comments

This paper examines $ER^{+}/HER2^{-}$ early breast cancer using spatial transcriptomics (GeoMx DSP), multiplex IF, and IHC in a large TAILORx cohort (n=577; 410 with ST; 442 with spatial proteomics) to identify immune–spatial features associated with recurrence risk and chemotherapy benefit. The manuscript is very difficult to read and contains several contradictions. The authors should temper their claims, as most findings relate to the TME in the primary tumor rather than distant micrometastatic sites, which are the targets of adjuvant endocrine therapy and chemotherapy. Furthermore, all results rely on a single dataset with no external validation (e.g. applying deconvolution on publicly available bulk data).

Reply: We would like to thank the reviewer for their comments on our manuscript and for allowing the time to strengthen our analysis.

Regarding external validation, we agree that this is a serious limitation of our analysis and have framed our findings as requiring extensive validation before clinical implementation can be seriously discussed. Furthermore, we would like to highlight that the difficulty in validation hinges on access to a cohort with randomisation of adjuvant treatment in patients with an Intermediate RS and, secondly, that a spatial derivation of scores is necessary. While we agree that deconvolution of bulk RNAseq is a valuable tool, there is no information on the spatial component. Secondly, public datasets such as TCGA are not randomised trials, which was a necessary part of our analysis to discover the predictive biomarker.

"High sTILs CD68" - Macrophages are not lymphocytes.

Reply: We now at all points refer to stromal and epithelial macrophage rather than 'sTIL' and 'iTIL'.

Show all dots on boxplots. Add p-values to boxplots.

Reply: We have updated figures to accommodate this change to the p-values.

Display actual FDRs and use consistent cut-offs. FDR<.1 is too lenient. p-values

should be shown with 1 or 2 significant digits, not 3 (e.g. not p=0.00237 as in the text) nor 0 (e.g. p=0.000 in fig 4a).

Reply: We now use 2 significant digits in the text, where appropriate, and have updated figures to prevent text from being cut. We continue to use an FDR<0.10 though frame our analysis as exploratory. We believe an FDR<0.05 is too conservative for an exploratory analysis and update the manuscript to explicitly state our analysis is exploratory in nature, requiring validation in a larger trial.

M&Ms are a bit generic at times (e.g. "Differences in values between two categories was assessed using pairwise Mann-Whitney or Student's t-test" - why 2 methods? I don't think any of the two was actually applied).
Reply: We have updated the materials and methods to be more explicit about which tests were used where and introduce more specific subheadings for each section.

Fonts on figures are way too small. Figures are very hard to read, which is made worse by the fact that some panels are bitmaps (not vector graphics). Some panels are almost impossible to read (e.g. S3c), even fully zoomed. Panel S4e is an extreme example - extremely small numbers in big boxes. Why this hatred towards the readers?

Reply: We agree that the figure font was at times too small to read. We therefore have updated all figures and associated text to be more legible in the embedded manuscript.

Comments on specific analyses

For the figures on distances (e.g. 2B) the distribution of individual cells is displayed. The experimental unit however is the sample, so statistics cannot be performed on such data, and to the authors credit it is indeed not performed. This is replaced by an effect size consideration, which I think is not good enough.
The distances can easily be summarized for each sample (e.g. the median, the % below a certain cut-off, whatever). Proper statistics can then be performed using that value.
I don't understand negative distances for macrophages (2c).
In general you would expect distance to be smaller with increasing amount of the cell types compared. This is not discussed.

Panels 2c-2e are not discussed in the text.

Forest plots are hard to read. P-values are at times cut on the right of the panel (e.g. Macrophages -> CD8 in 2f).

Fig 2f, "Facilitation of sTILs CD68 to iTILs CD68" is a really bad x-axis label. The model is impossible to understand without checking the details in the M&Ms, where it is hard to find (in the middle of a 2.5 pages-long "statistical analysis" section).

Reply: We have updated Figures 2 and 3 (pages 8 and 12, respectively) to accommodate suggestions from other reviewers. As such, the above points have been addressed.

Regarding distances, we have moved the distance analysis to Fig.6B-D (page 25) and update the figure to reflect patient-level data with the proper statistics applied (Kruskal-Wallis with two-sided Dunn's tests). We now stratify by TME types derived earlier in the updated manuscript. We have also included a new panel (Fig.6C) that shows the log-log relationship between sTIL and iTIL densities (~1 shows that iTIL rises proportionally with sTIL, <1 shows sub-proportional scaling, and >1 super-proportional scaling), stratified by TME state. We believe this to be a more intuitive representation of the information disclosed in the previous Figure 2f. For clarity in interpretation, we also include a dedicated subheading in our methods section, on page 36, "Stromal-Epithelial Coupling of Immune Infiltrates".

Fig 2g - not clear if expression is in tumor or stroma. Done as a regression analysis, but would be more natural to use correlations. Correlations would allow to use non-parametric testing as well as to actually compare the correlations across treatment arms. Also, scatterplots should be shown for all pairs in supplementary and probably the most interesting ones in the main figure.

Reply: We have moved this analysis to updated Figure 6 and update the plots to show more clearly whether the expression is in the tumour/epithelia or stroma/TME compartment. Secondly, as we update this analysis to reflect changes across TME/EPI states, we no longer use strata of received adjuvant treatment. Lastly, we retained a regression analysis as our primary aim was to quantify the conditional association between immune cell density and gene expression, while adjusting for other cell type

densities within the same analysis compartment, thereby separating changes in expression from changes in cellularity/composition. This provides an interpretable effect size that is not obtainable from unadjusted correlation analyses.

Fig 3a - the lower line of the x label is actually the y labels. This is very confusing. The insert (density subplot) overlaps with some dots and might hide others.

Reply: This has been removed from the figures with the aforementioned updates to previous figures.

Fig 3d - it should be described how the module scores were computed (mean of the genes?).
It is stated that the model controls for immune densities, however immune densities (e.g. CD8 density) are shown - this is explained in the M&Ms but not in text or legend and is confusing.
Also, my understanding is that the regression uses a distance computed by sample. How was this distance computed? And why was it not used in the other analyses?
I don't understand the R2 analysis. All error bars overlap anyway.

Reply: We agree that the details of this analysis needed to be clearer. Therefore, we now explicitly define gene-module scoring in the figure legend (Figure 6, page 25: "*Gene modules are defined as the mean expression of constituent genes in the TME/stroma: Cytotoxic = GZMA, GZMB, PRF1, IFNG. Checkpoints = TIGIT, LAG3, CTLA4, CD274, PDCD1, PDCD1LG2, VSIR. Exhaustion = TOX, CXCL13, HAVCR2)* and materials and methods. We further clarify that in the regression analysis we adjust for all immune cell densities within the spatial compartment except the predictor being tested (i.e. leave one out adjustment). For example, when testing the association between CD8 density and the stromal module score, the model includes all stromal densities as covariates except for stromal CD8 density, so that the coefficient represents an association that is not simply driven by changes in the cellularity of other phenotypes in the respective compartment. We have updated the figure legend to make this explicit (Figure 6, page 25: *"Models include stromal immune cell densities and proximity metrics,*

with TME/EPI state and Oncotype Dx RS as covariates, and were adjusted for variables except the predictor of interest.).”

For densities, nearest-neighbour distances were computed in QuPath as 2D Euclidean distances between cell centroid coordinates and are summarised as the median distance per patient. This was used in the regression modules. These were not used in all analyses because they were defined to provide mechanistic insight to transcriptomics hypotheses.

Finally, the R^2 analysis reports incremental (i.e. partial) R^2. This is the change in explained variance when adding the given predictor to a covariate-adjusted base model by bootstrapped resampling (5,000 replicates). Error bars indicate the bootstrapped 95%CI. We agree that the R^2 differences are modest and temper our emphasis accordingly. We have opted to retain this information as barcharts in figures, as this data is a descriptive complement to the effect size estimates.

Fig 3e - x label is "treatment", I assume it should be "Oncotype DX"
Reply: This has been removed from the figures with the updates to previous figures. This analysis is now in Figure 6 (page 25) and reflects gene expression changes across TME/EPI states introduced in the updated Figure 2 (page 8).

Fig 3f - use log x-axis. P-values are cut on the right (not displayed). There is no way that the p-values reported have FDR < 10% as stated (60 comparisons, most significant p-values are ~ 1% and there are not many of those).

Reply: This has been removed from the figures with the updates and moved to the Supplement (Fig.S8), where we now also use a log x-axis and the annotated p-values are no longer truncated. We have also updated the forest plot of the Intermediate RS in Figure 4A to use a log x-axis (page 17).

Regarding correction for multiple comparisons, we control the FDR using Benjamini-Hochberg within each Oncotype Dx RS group, and separately for epithelia vs TME features. We treated these as distinct hypothesis families *a priori* as: 1) each ODx

category is a distinct clinical stratum with different biology, baseline risks, and treatment implications, 2) epithelial and stromal/TME features were derived from separate segmentation masks (similarly with spatial transcriptomic features) and were analysed as compartment-specific biological questions.

Fig 4a - x axis should be in log.
My understanding is that this analysis was done on the randomized arms because of "Investigating immune variables across randomised arms (Fig.4A)" in the text, however there is nothing about this in the figure legend.
It is a surprising that the error bars are so much larger in the HT arm. The reason for this seems to be that patients with HT+CT have much worse prognosis than patients with HT only (fig 4c). Was this really randomized?
I find the result from the interaction test surprising. Usually the p-value from the interaction can be roughly estimated by comparing the CIs in the two groups. For CD8 density those CIs overlap - the point estimate for CT+HT is actually in the CI of HT. However, the interaction test is given as significant (p=.007). Was that done on the same data?
Also, the interaction test should be corrected for multiple testing, as clearly those were hand picked as the most likely to be significant.

Reply: We thank the reviewer for their comments. We have updated the figure legend to reflect randomisation (Figure 4, page 17). The Intermediate RS was indeed randomised to adjuvant treatment, and we now clarify more explicitly that this analysis uses the randomised treatment categories of the Intermediate RS in the text body and figures. Secondly, to address the concerns around baseline imbalances in the Oncotype Dx RS across randomised arms, we compared the distributions of discrete Oncotype Dx RS scores in these strata. We found no significant difference in ODx RS across treatment categories of the Intermediate RS (two-sided Kolmogorov-Smirnov test (D=0.087, p=0.755), consistent with successful randomisation.

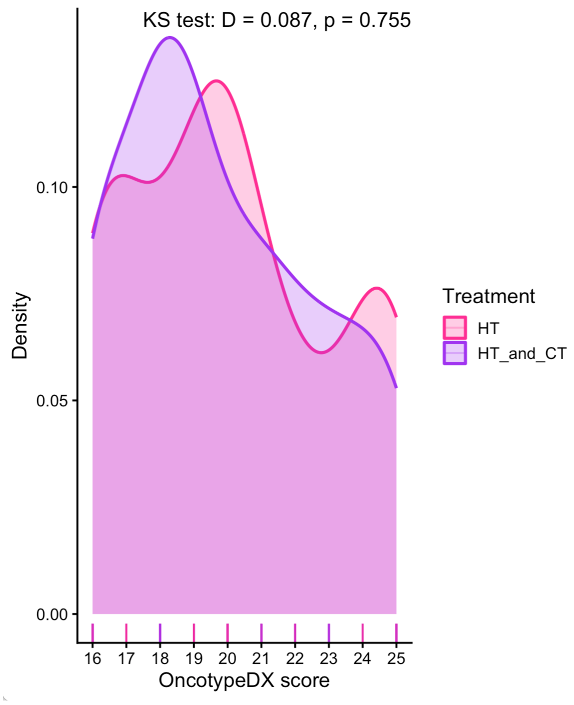

Figure 4A shows hazard ratios and 95%CI from multivariable Cox models adjusted for the same set of prespecified clinical covariates. Differences in confidence interval width between arms reflect differences in precision driven primarily by the number of events in each arm. Regarding the interaction test, the reported p-value is derived from a formal likelihood ratio test (ΔLR-χ^2) comparing Cox models with and without a treatment-biomarker interaction term, with the biomarker modelled continuously and adjusted for the prespecified covariates. This tests whether the biomarker effect differs by treatment arm, which is not equivalent to visually comparing the overlap in subgroup-specific confidence intervals. The overlap of CIs between randomised arms is not a reliable test of interaction. The interaction test and plotted estimates were computed on the same dataset, and we now include FDR correction in both the annotated text in plots (Fig.4E-F), figure legends, and results text.

Fig 5 - I think that claiming that chemotherapy has a negative net benefit is pretty wild (fig 5g).
Patients were not randomized, so we can expect patients receiving HT+CT to have a worse disease. So in general CT+HT should be worse than HT, not because treatment is bad but because of patient selection.

Lots of cytotoxic Tcells -> higher Ki67 (on average). So those with low density will be much more likely to be lumA, for who CT is not necessarily given but can help. Which may be what is seen here.

Reply: We thank the reviewer for their comments and for raising this important point. To be clear, the validation analysis uses whole-slide images of resection specimens of the randomised Intermediate RS only. We have updated the text on page 18 to be explicit that our analysis uses this data.

Furthermore, we agree that decision curve analysis (DCA) does not imply that chemotherapy is intrinsically harmful. Rather, it evaluates the net utility of recommending chemotherapy at different risk thresholds. We have therefore tempered our wording to describe these findings as suggestive of reduced clinical utility of chemotherapy at higher stromal CD8 densities and further emphasise across the manuscript the need for external validation. Secondly, to address the possibility that the biomarker effect/observed interaction is driven by more Ki67/Luminal B-like biology, we have adjusted the models shown for Ki67. The biomarker-treatment interaction remained significant in both discovery (ΔLR-χ^2: 6.79, p=0.009, q=0.018) and orthogonal validation datasets (ΔLR-χ^2: 8.90, p=0.003). These results indicate that the association is not explained only by Ki67 enrichment.

Reviewer #4 (Remarks to the Author):

I co-reviewed this manuscript with one of the reviewers who provided the listed reports. This is part of the Nature Communications initiative to facilitate training in peer review and to provide appropriate recognition for Early Career Researchers who co-review manuscripts.

Author Rebuttal Comments:
Spatial-analyses implicate high stromal tumour-infiltrating $CD8^+$ lymphocytes as a negative predictive marker for chemotherapy in estrogen receptor-positive breast cancer

REVIEWERS' COMMENTS

Reviewer #1 (Remarks to the Author):

The manuscript has substantially improved following revision. The authors have adequately addressed the concerns and suggestions raised during the initial review.

Reply: We thank the reviewer for their time and assistance in review and for providing direction that has strengthened our manuscript.

Reviewer #2 (Remarks to the Author):

My comments have largely been addressed and it is clear that substantial work has been done in the revision, generally strengthening the manuscript. However, I would urge the authors to carefully go through text and figures again to make both more reader friendly. Even though there has been some improvement often text is still too tiny (e.g. many figure legends in Fig 4). It should be easily fixable by editing manually in inkscape for example.
In the text, especially the new passages there seem to be some “careless mistakes”. Eg. “Using spatial proteomic data (n=440), we discovered that no TME (stromal tumour-infiltrating lymphocyte: sTIL) phenotype density was significantly different across the RS except for sTIL T-regulatory cells [...] “(stromal tumour-infiltrating lymphocyte: sTIL)” seems to be at the wrong spot or if it is at the right spot it is very confusing the way it is written.

Reply: We thank the reviewer for their time and assistance in review and for providing direction that has strengthened our manuscript. We address their remaining points, below:

We have updated the text on page 4-5 to reduce confusion when interpreting the first mention of sTIL and iTIL.

We have gone through our manuscript and related figures to reduce visual clutter and aid in interpretation. E.g. Figure 4, we have consolidated figure legends and keys and

increase the text size to make the figure more easily interpretable.

Reviewer #3 (Remarks to the Author):

The authors addressed several of my and other reviewers' comments and significantly improved their manuscript. I do not have additional comments.

Reply: We thank the reviewer for their time and assistance in review and for providing direction that has strengthened our manuscript.

Reviewer #4 (Remarks to the Author):

I co-reviewed this manuscript with one of the reviewers who provided the listed reports. This is part of the Nature Communications initiative to facilitate training in peer review and to provide appropriate recognition for Early Career Researchers who co-review manuscripts.

Reply: We thank the reviewer for their time and assistance in review and for providing direction that has strengthened our manuscript.
